# Supplementary material for: Perception of motion salience shapes the emergence of collective motions
Source: Nat Commun. 2024 Jun 5;15:4779. doi: 10.1038/s41467-024-49151-x (PMC11153630; doi:10.1038/s41467-024-49151-x)
Supplement: Supplementary file 1 — Supplementary Information [file 41467_2024_49151_MOESM1_ESM.pdf]

Perception of Motion Salience Shapes the Emergence of Collective Motions

Supplementary Notes 2

1. Data collection and processing of three original flocking datasets 2
2. Using leader-follower relation matrix to classify sub-communities in the circling flocks 5
3. The flocking metrics analyzed in three datasets 5
4. Calculation of the nestedness of leader-follower relation matrix 7
5. Perception of motion salience reflects variations in heading, speed, and acceleration over a period of flock 10
6. Spatial structure of leading position 11
7. Correlation analysis of MS-Speed and LF-Speed 12
8. The effectiveness of leadership in AMS interaction 14
9. Experimental set-up of swarm robotics 15

Supplementary Tables 20

Supplementary Figures 22

Supplementary References 76

Supplementary Notes

1. Data collection and processing of three original flocking datasets

1.1 Workflow of data processing for the original flocking datasets

In this work, we apply our modeling framework for three bird flocking datasets, named mobbing[1], circling[2] and transit[1] flocks. Three original flocking datasets consisted of many periods of 3D tracks, which were reconstructed from video recordings using arrays of high-speed cameras[3]. In the original flocking datasets released from academic papers[1,2], 3D tracks of flocks were stored by text format with the movement information about absolute (x, y, z)-position and corresponding (v_x, v_y, v_z)-velocity for individuals at time stamp t.

To unify different text formats of 3D tracks from three datasets in this work, Supplementary Figure 1 shows the workflow of data processing of the original flocking datasets. For example, for a given text file storing the entire information of flocking trajectories from a period of video recording, we repeat the workflow for each frame (equals the minimal time interval of the video recording) to yield the frame matrix (Supplementary Figures 2-4). Here the frame matrix records the effective information of all individuals appeared in a flock. Finally, for each dataset, the workflow generates many periods of flocks to be analyzed in our modeling framework (Supplementary Figures 5-9). See Supplementary Table 2 for the descriptions of terms or notions used in the data processing.

(i) **Generate the unified tracks_filt matrix to store the flocking trajectory information.** Inspired from the storage structure of 3D tracks proposed by jackdaw flocks[1], we convert the raw text format of 3D trajectories to a unified tracks_filt matrix as below (hereinafter we use Matlab syntax):

```
tracks_filt(:,1) = individual id,
tracks_filt(:,2:4) = individual position in Cartesian coordination,
tracks_filt(:,5) = time stamp,
tracks_filt(:,6:8) = individual velocity,
tracks_filt(:,9:11) = individual acceleration.
```

Note that the time stamp corresponds to a frame in the video recording, and the difference between two adjacent time stamps indicates the minimal time interval of the video recording (also called the video's frame rate).

(ii) **Generate the individual present matrix.** For a text file recording 3D flocking trajectory, we generate the individual present matrix where the rows indicate the individuals and the columns represent the time stamps (equals the frame in a video recording). For example, in the individual present matrix, the number of rows is the unique number of `tracks_filt(:,1)` to represent how many individuals appear in the flock; the number of columns equals the unique number of `tracks_filt(:,5)` to represent how long the flock lasts. Therefore, if bird-i is present at time t, the element (i, t) in the individual present matrix stores the row number of `tracks_filt` matrix to map the movement information of bird-i at time t. Otherwise, the element (i, t) in the

individual present matrix is `null`. Note that there could be many null-elements in the individual present matrix. See Supplementary Figure 1b for the individual present matrix of a track named “mobbing-01”.

(iii) **Run the selection and trimming procedure for a frame of individual present matrix.** If one observes the individual present matrix generated in step (ii), there could exist many blanks. It is because that: the time of original video recordings lasts a long period (i.e., from several minutes to half hour in three datasets); and the individuals perhaps moves beyond the observation range. Therefore, the original tracks are not suitable to be analyzed by our framework, and need to be trimmed into many short periods of continuous tracks. For example, if we pick up a frame (equals a column) from the individual present matrix (i.e., red line indicates “$\text{Frame time} = 70.1000s$” in Supplementary Figure 1b), some adjacent frames could yield a period of continuous flocking trajectories from the whole video recording. Here the period of continuous trajectories is referred to as frame matrix. Therefore, to yield the short periods of continuous tracks from the original track, we run the selection and trimming procedure as following (Supplementary Figure 1c).

First, generate a null frame matrix.

Second, for a new frame, the selection procedure should simultaneously meet two criteria: the number of individuals is larger than 10, and this frame appears 25% new individuals compared with that of previous frame matrix. If yes, go to the next trimming procedure; if no, the next step terminates and restarts the selection procedure for next frame.

Third, continue to run the trimming procedure. The diagram of trimming procedure is shown in Supplementary Figure 1c.

(1) Extract the present N individuals in this frame from the individual present matrix as a `sub_matrix` and relabel the bird index from 1 to N.

(2) Discard some columns in `sub_matrix` if

`sum(sub_matrix~=0,1) < size(sub_matrix,1)/2`.

This step makes sure that there are at least half of N individuals at every time stamp in the `sub_matrix`.

(3) Discard some rows in `sub_matrix` if

`sum(sub_matrix~=0,2) < mean(sum(sub_matrix~=0,2))`

and relabel the bird index in new `sub_matrix` from 1 to N. It guarantees that each bird appears in the `sub_matrix` at least half of flocking duration.

(4) Discard some columns in `sub_matrix` if

`sum(sub_matrix~=0,1) ≠ size(sub_matrix,1)`

and relabel the bird index in new `sub_matrix` from 1 to N. It ensures that each bird is present in the `sub_matrix` from beginning to end.

At the end, we record the final `sub_matrix` as frame matrix for the given frame time. The frame matrix is the sub matrix of individual present matrix. Supplementary Figure 1c shows these four steps are applied to generate the frame matrix corresponding to the flock of Fig.1a in the main text.

(iv) **Repeat the selection and trimming procedure frame by frame on the individual present matrix.** After repeating the above procedure frame by frame, the whole track could generate many frame matrices to perform our modeling framework. For example, Supplementary Figures 2-4 show all the frame matrices (highlighted by red boxes) trimmed from 12, 5 and 3 video recordings of mobbing[1], transit[1] and circling flocks[2], respectively.

Finally, using the workflow of data processing, we totally generate 140 tracks (the number equals the number of frame matrix) of mobbing flocks (Supplementary Figure 5), 94 tracks of transit flocks (Supplementary Figure 6), and 232 tracks of circling flocks (Supplementary Figures 7-9) from the original flocking datasets.

1.2 Mobbing flocks trimmed by our workflow of data processing

The original mobbing dataset consisted of 12 video recordings of 3D flocking trajectories. Supplementary Figure 2 shows the individual present matrices of 12 video recordings of mobbing dataset and highlights the frame matrices generated by the above workflow using red boxes. We totally get 140 short periods of continuous tracks (equals to 140 frame matrices) from the original mobbing dataset to be analyzed by our modeling framework. Supplementary Figure 5 displays 3D trajectories of 140 mobbing tracks. Mobbing, also called collective anti-predator events, shows the highly maneuverable movements with time evolving.

1.3 Transit flocks trimmed by our workflow of data processing

The original transit dataset consisted of 6 video recordings of 3D flocking trajectories. Due to the same recording between transit-03 and transit-04, we just analyze 5 video recordings. Supplementary Figure 3 shows the individual present matrices of 5 video recordings of transit dataset and highlights the frame matrices generated by the above workflow using red boxes. We totally get 94 short periods of continuous tracks from the original transit dataset to be analyzed by our modeling framework. Supplementary Figure 6 displays 3D trajectories of 94 transit tracks. Different with mobbing and circling flocks, the transit flocks show the highly ordered and smooth motion pattern.

1.4 Circling flocks trimmed by our workflow of data processing

The original circling flocking dataset consisted of 3 video recordings of 3D flocking trajectories. Supplementary Figure 4 shows the individual present matrices of 3 video recordings of circling dataset and highlights the frame matrices generated by the above workflow using red boxes. We totally get 232 short periods of continuous tracks from the original circling dataset (see Supplementary Figures 7-9 for 3D trajectories of 232 circling tracks). Note that 232 short periods of continuous tracks could not be directly analyzed by our modeling framework since the circling flocks contain many sub-communities. Therefore, they require a further step to classify the sub-communities and then to be analyzed by our framework. See Supplementary Note 2 for how to classify the sub-communities in the circling flocks.

2. Using leader-follower relation matrix to classify sub-communities in the circling flocks

As the original circling flocks record hundreds of swifts which are mixed together to behave the circling pattern near a roost site, Supplementary Figure 10a displays that the swifts form a few sub-communities due to different locations of individuals in the whole flock. It is consistent with the observation of sub-communities in Supplementary Movie 2. (Note that Supplementary Movies 1-4 reconstruct the flock by 3D game engine called Unity[4].) For example, Supplementary Figure 10c shows the trajectories of those sub-communities. We find that the individuals in a sub-community are close to each other at the beginning of this video recording, and then they hover as a sub-group. Across different sub-communities shown in Supplementary Figure 10c, each sub-community is worth analyzing MS and LF instead of that of the whole flock. Therefore, the question is how to classify sub-communities of the 232 circling flocks shown in Supplementary Figures 7-9.

Interestingly, the LF relations could classify the whole circling flock into sub-communities, because different individual pairs with similar LF relations could demonstrate that those pairs have the similar leading or lag time of LF relations. For instance, after detecting the modularity of LF matrix derived from the flock shown in Supplementary Figure 10a, each module corresponds to a box in Supplementary Figure 10b and a 3D box in Supplementary Figure 10c. As both the row and column of LF relation matrix are the same, the intersection set of row and column of each module represents the individuals belonging to this module. Each 3D box in Supplementary Figure 10c shows the corresponding 3D trajectories of a module (also called sub-community). If the intersection set is null, this module is empty, i.e., empty boxes in Supplementary Figure 10c. Besides, we find that the value of τ_{ij}^{LF} for those empty modules in Supplementary Figure 10b are almost either the largest (yellow) or the least (dark blue).

In this work, we use the BiMat[5] (a Matlab code package to calculate the modularity and nestedness of complex networks) to calculate the modularity of LF relation matrix. Finally, the 232 circling flocks are classified into 1483 non-empty modules to be analyzed by our modeling framework.

3. The flocking metrics analyzed in three datasets

3.1 Four flocking metrics

In this work, we totally generate 140, 94, and 232 short periods of continuous tracks from mobbing, transit, and circling datasets, respectively. The 140 and 94 flocks from mobbing and transit could be directly analyzed by our modeling framework. Due to the sub-communities in circling flocks, we first apply the modularity analysis for these 232 flocks one by one, and finally yielded 1483 circling flocks to feed our modeling framework. Note that in the main text and hereinafter, the terms of "mobbing, circling and transit datasets (or flocks)" represent the 140, 94 and 1483 flocks. Supplementary Figure 11 shows the flock size and recording time of all flocks analyzed in this

work of three flocking datasets. We use four metrics, e.g., group order, trajectory curvature, group density and instability of neighbor to systematically analyze the collective motion characteristic.

- The average order over the recording time is calculated by $\sum_{i=1}^{N} \frac{\hat{\mathbf{v}}_i(t)}{N} \in [0,1]$ to reflect the group polarization. The higher average order, the higher consensus of collective motions. We find that the group polarization in descending rank is transit, circling and mobbing, which is consistent with the observation of flocking trajectories. Especially for transit datasets, the median of average order is very close to 1, and the minimal value is larger than 0.83.
- The trajectory curvature aims to quantify the flocking maneuverability in the forms of, $\kappa_i(t) = \frac{|\mathbf{x}_i(t)' \times \mathbf{x}_i(t)''|}{|\mathbf{x}_i(t)'|^3}$, where $\mathbf{x}_i(t)$ is position of individual-i at time t in 3D Cartesian coordinate, and the prime denotes differentiation with respect to time t. The flocking curvatures shown in Fig.2j is first to average the temporal curvatures of each individual over time as $\kappa_i = \langle \kappa_i(t) \rangle$, and then to average κ_i over all individuals in the flock.
- Group density is calculated by $6N/(\pi\langle d_i \rangle^3)$, where N is the group size, d_i is he metric distance from bird i to its most distant neighbor, and $< >$ denotes an average over all birds in the group.
- Instability of neighbor $Q_M(t)$ is estimated by measuring how much the set of nearest M neighbours of a focal individual $M_i(t)$ changes over time[6]:

$$Q_M(t) = 1 - \frac{1}{t}\frac{1}{N}\sum_{t_0=1}^{t-1}\sum_{i=1}^{N}\frac{|M_i(t_0) \cap M_i(t)|}{M}$$

  where N is the number of flock members, $M_i(t_0) \cap M_i(t)$ is the intersection of the closest neighbors of individual i at time t_0 and t. We average over all individuals in the flock and all initial time points t_0 during $[t_0, t]$. Based on the above equation, the instability of neighbor ranges from 0 (if all neighbors are the same) to 1 (if all neighbors have changed over the given time period).

Supplementary Figure 12 shows four temporal metrics for the mobbing and transit flock shown in Fig.1 as examples. Overall, these metrics successfully quantify the highly maneuverable or smooth motions.

Then we extend four metrics to three datasets. The ascending order of average order and the descending order of average trajectory curvature is mobbing, circling and transit datasets (see Supplementary Figure 13a,b). The results are consistent with the observations of flocking trajectories: mobbing and circling flocks display highly maneuverable motion, i.e., collective sharp turn to drive away predator or collective circling near a roost site, while transit flocks are much smoother and highly ordered to move towards the winter roosts. Interestingly, we find that the group density does not show any difference across three datasets, regardless of whether the flocks exhibit collective turns or flying smoothly (Supplementary Figure 13c). However, for the instability of neighbors[6], the transit is significantly lower than that of mobbing and circling flocks (Supplementary Figure 13d). The result suggests that the neighbor structure in transit flocks is

much more stable than the behaviors of collective turns. Since the shape of flock changes frequently during collective turns, individuals may need to turn to avoid collisions more often. Oppositely, those foraging individuals know where they are going and they are already in an ordered flock.

3.2 The relations between different flocking measurement across three datasets

We test the relations between different flocking measurement across three datasets. The relation between trajectory curvature and group order accords with our common sense and the observed collective behaviors across three datasets: the higher group order, the less trajectory curvature (Supplementary Figure 14). As instability of neighbors measures the changes of neighbor structure over time, we focus on analysis of the relations between instability of neighbors and other flocking metrics that describes motion characteristic (Supplementary Figure 15). It shows that:

(i) In circling and transit dataset, the instability of neighbors decreases with the increment of group order (negative trend in Supplementary Figure 15a), but the relation is not clear in mobbing flocks.

(ii) Instability of neighbors grows with trajectory curvature (positive trend in Supplementary Figure 15b) in mobbing and circling datasets, but it is not clear in transit flocks.

(iii) Three datasets all emerge that instability of neighbors increases with the increment of group density (positive trend in Supplementary Figure 15c), that is, the flock is denser, the neighbors of a focal individual changes more frequently.

Based on the above empirical analysis, we could draw the conclusion that:

(i) irrespective of the collective motion's maneuverability or smoothness, an increase in group density leads to frequent changes in neighbors. This phenomenon could be attributed to the necessity for individuals to actively avoid collisions in dense environments.

(ii) flocks characterized by highly maneuverable motion exhibit a distinct tendency that the bigger trajectory curvature, the more instability of neighbors for a focal individual. Interestingly, this trend does not manifest in transit flocks.

4. Calculation of the nestedness of leader-follower relation matrix

4.1 The introduction of NODF to calculate the nestedness

Nestedness describes the extent to which interactions form ordered subsets of each other. In this work, we use NODF (Nestedness metric based on Overlap and Decreasing Fill)[7] to quantify the nestedness of LF networks. Note that we ignore the weight of LF relation matrix to quantify the nestedness, that is, setting the elements $\tau_{ij}^{\mathrm{LF}} < 0$ in the LF matrix as 1.

The NODF normalizes the matrix size, and thus allows matrices of different sizes to be compared. The nestedness range is $0 \le \mathrm{NODF} \le 1$, where 0 indicates a matrix of blocks and 1 corresponds to a perfectly nested structure. NODF is based on two properties: decreasing fill and

paired overlap. NODF measures the nestedness across rows by assigning a value N_{ij}^{row} to each pair (i,j) of rows in the interaction matrix:

$$N_{ij}^{\mathrm{row}} = \begin{cases} 0 & if\ k_i \leq k_j \\ \dfrac{n_{ij}}{\min(k_i, k_j)} & otherwise' \end{cases}$$

where k_i is the number of ones in i-th row, k_j is the number of ones in j-th row, and n_{ij} is the number of shared interactions between i-th and j-th rows (so-called paired overlap). Note that the positive contributions to NODF require pairs of columns satisfying the decreasing fill property, that is, when $k_i > k_j$, $N_{ij}^{\mathrm{row}} > 0$. A similar term N_{ij}^{col} is used to compute column contributions. The total nestedness is the sum of contributions from m columns and n rows:

$$\mathrm{NODF} = \frac{\sum_{ij} N_{ij}^{\mathrm{row}} + \sum_{ij} N_{ij}^{\mathrm{col}}}{\frac{m(m-1)}{2} + \frac{n(n-1)}{2}}.$$

In this work, we use the BiMat[5] to calculate the nestedness of LF matrix based on NODF method.

4.2 The introduction of Spectral Radius (along with a variant called normalized Spectral Radius) to calculate the nestedness

The Spectral Radius metric[8] is a nestedness measure that relies on the spectral properties of double nested graphs. This metric utilized a theorem that states that within the connected bipartite graphs with $n + m$ nodes and E edges, the graph (represented by the form of an adjacency matrix) that yields the highest spectral radius is indicative of a perfectly nested structure. Notably, Ref.[8] demonstrated that graphs with higher levels of nestedness tend to have larger spectral radius, although this relationship is not strictly monotonous. Interestingly, this metric overcomes certain limitations present in previous metrics. Since it involves diagonalizing a symmetric matrix, it is not affected by the ordering of the matrix and avoids any ambiguities associated with determining the maximally packed form of the bipartite matrix encountered in other nestedness metrics. Of course, the import drawback of Spectral Radius metric is not normalized. Ref.[9] reported a method to get the normalized Spectral Radius (denoted as n-Spectral radius). That is, if we denote λ as the spectral radius of a real network and $\lambda_{\max}$ as the spectral radius of a perfectly nested graph with the same size and fill, the normalized index n-Spectral radius can be calculated as $\lambda/\lambda_{\max}$.

The LF networks of flocks exhibit a wide variability in terms of group size and edge density, and these two variables could have distinct effects on the Spectral Radius and NODF metrics respectively. Supplementary Figure 17 shows three nestedness metrics (NODF, Spectral radius and n-Spectral radius) as a function of group size, density of edges in LF networks for the Mobbing dataset. We find that there is a strong linear correlation between NODF and edge density (Supplementary Figure 17b), between Spectral radius and group size (Supplementary Figure 17c). To clearly demonstrate these relations, we show four examples of LF networks labelled as I, II, III, IV in Supplementary Figure 17. In the cases of example-I, II and III, as the edge density is similar (x-axis of Supplementary Figure 17b), we observe that their corresponding NODF values are also comparable (y-axis of Supplementary Figure 17b). However, the Spectral Radius values (y-axis of Supplementary Figure 17c) differ significantly due to variations in their group sizes (x-axis of

Supplementary Figure 17c). Indeed, because example-III and IV have similar group sizes, their Spectral Radius values also exhibit a close resemblance. Note that there is a substantial disparity in the nestedness between these two examples. In Supplementary Figure 17g, we compare the nestedness metrics of NODF and Spectral Radius to illustrate the impact of group size on these two metrics. To visually emphasize this impact, the point size in Supplementary Figure 17g linearly scales with the corresponding group size. Interestingly, By utilizing the normalized Spectral Radius, the influence of group size on the Spectral Radius metric is eliminated (Supplementary Figure 17e,f). This allows for a more focused assessment of the nestedness without being confounded by variations in group size. Obviously, the normalized Spectral Radius effectively eliminates the influence of group size on the metric. Therefore, we use normalized Spectral Radius as comparison with NODF.

4.3 Comparisons of nestedness against 4 null models with various degree constraints of LF networks

Since the nestedness is strongly related to the network structure, the null models with various degree constraint of LF networks could play the crucial role in accurately inferring and interpreting the structural patterns in nestedness analysis. Here we introduce 4 null models with various degree constraint of LF networks.

(i) Null-1 Equiprobable: the connectance (proportion of existing interactions) of newly generated network is preserved. However, the number of interactions in which each node participates is not specifically controlled in this context. It is the same with null mode-(i) in Ref.[8].

(ii) Null-2 Average: In the newly generated network, both the connectance and the expected number of interactions for each node are preserved. It is the same with null mode-(ii) in Ref.[8].

(iii) Null-3 Rows Average: In the newly generated network, both the connectance and the expected number of interactions of column nodes are preserved.

(iv) Null-4 Fixed: In the newly generated network, both the connectance and the degree distribution of each node (not just their expected number of interactions) are preserved. It is the same with null mode-(iii) in Ref.[8].

Supplementary Figure 18a and Supplementary Figure 19a show the original LF network and 4 null modes for example-I,II,III,IV. Since Null-4 preserves the overall structure and degree distribution of the original network to a great extent, Null-4 is only able to generate networks that are identical to the original network in the case of perfectly nested LF networks (example-I in Supplementary Figure 18a). For near-perfectly nested networks, Null-4 still produces networks that exhibit minimal changes compared to the original network (example-I,II,III in Supplementary Figure 18a).

If using NODF (Supplementary Figure 18b-e), we find that average NODF of Null-4 (100 independent randomizations) is extremely close to real NODF in the entire range of [0,1] (Supplementary Figure 18e). For normalized Spectral Radius, average value of Null-4 is also extremely comparable to that of real LF network (Supplementary Figure 19e). Two results

calculated by different nestedness metrics are consistent. For the other 3 null models, their average NODF of only match real NODF in the range [0,0.2]; and as the real NODF increases, there is a growing difference between the average NODF and the real NODF (Supplementary Figure 18b-d). This indicates that the null-4 model with fixed degree distribution effectively preserves the nestedness patterns present in the original network.

Moreover, Supplementary Figure 18f-i (or Supplementary Figure 19f-i) show the z-score of real NODF (or real normalized Spectral Radius) value within the distribution of random NODF (or random normalized Spectral Radius) values generated from 4 null models. We find that except Null-4, more nested LF networks (representing higher NODF or normalized Spectral Radius) exhibit higher z-scores with respect to their randomized counterparts.

5. Perception of motion salience reflects variations in heading, speed, and acceleration over a period of flock

Although MS equation does not directly incorporate variations of velocity and speed, we find that it effectively incorporates these variations by considering the relative position changes between the starting and ending timestamps. This is due to the fact that the relative position changes between two timestamps could indicate the variations in heading, speed, and acceleration over a period of flock. Here we report two kinds of correlation analysis to validate our statements.

(i) correlation analysis between MS and average velocity consensus (AVC) of each pair of birds within a time period $[t-\tau, t]$. AVC, denoted as $\phi_{ij}(t,\tau) = \langle \mathbf{v}_i(t) \cdot \mathbf{v}_j(t) \rangle_\tau$, directly describes the consensus of two birds' velocities over $[t-\tau, t]$. A higher ϕ_{ij} value indicates a greater velocity consensus within a bird pair. Then we compute the Spearman correlation coefficient of two vectors consisting of $M_{ij}(t,\tau)$ and $\phi_{ij}(t,\tau)$ of all bird pairs. For a given flock, we expand the correlation analysis between MS and AVC for various combinations of t and τ, as illustrated in Supplementary Figure 23b. We find that, in the flock depicted in Supplementary Figure 23a, the Spearman correlation between MS and AVC across different combinations of t and τ predominantly exhibited negative values. Similarly, the correlation analysis between MS and AVC in 3 bird-flocking datasets consistently demonstrates a prevalence of negative correlation (Supplementary Figure 23d-f). The negative correlation between MS and AVC aligns with our intuition, as increased motion salience within a bird pair would likely result in decreased velocity consensus.

(ii) correlation analysis between MS and distance of temporal speed (DS) of each bird pair within a time period $[t-\tau, t]$. Here the DS quantifies the distance of two vectors from two birds' temporal speed within the interval $[t-\tau, t]$, i.e., $\mathcal{D}_{ij}(t,\tau) = \text{Distance}\left(\text{vec}(v_i(t)), \text{vec}(v_j(t))\right)$, as shown in Supplementary Figure 23a(ii). If two birds closely align within a time interval $[t-\tau, t]$, their temporal speeds, particularly the pattern of speed variation are expected to be similar, resulting in a reduced DS value, e.g., see inset of Supplementary Figure 23a(ii). Therefore, we calculate the Spearman correlation

of two vectors consisting of $M_{ij}(t,\tau)$ and $\mathcal{D}_{ij}(t,\tau)$ of all bird pairs. For simplicity, the DS value $\mathcal{D}_{ij}(t,\tau)$ is calculated by the Euclidean distance. For a given flock, we expand the correlation analysis between MS and DS for various combinations of t and τ, as illustrated in Supplementary Figure 23c. We find that, different from the correlation between MS and AVC as shown in Supplementary Figure 23b, the correlation between MS and DS predominantly exhibits positive values. We then perform the correlation analysis for all the three bird-flocking datasets, finding similar results (see Supplementary Figure 23g-i). These results suggest that a higher MS value for a bird pair corresponds to a greater difference in temporal speed. Of course, this positive correlation also accords with our common sense, because larger relative motion changes in a bird pair are associated with greater changes in speed patterns.

6. Spatial structure of leading position

The spatial structure of leading position aims to investigate the relations between leading tier and fraction of neighbors present in the front view.

First, we focus on how to calculate the fraction of neighbors present in the front view through the projection of relative position with spherical coordination. For example, at time t, the fraction of neighbors appears in the front view of the focal individual-i is calculated by 3 steps:

(i) Construct a relative 3D Cartesian coordination from the view of the focal individual-i where the origin is the center of individual-i and the direction of x-axis follows $\mathbf{v}_i(t)$ (Supplementary Figure 24b).

(ii) Convert the absolute position of neighbor-j to the position relative to the focal individual-i by the representation of spherical coordination. The absolute position of $\mathbf{x}_j(t)$ could be converted to $(r_{ij}^t, \theta_{ij}^t, \phi_{ij}^t)$ in the relative 3D Cartesian coordination constructed in step (i). Between the focal individual-i and neighbor-j at time t, r_{ij}^t is distance, $\theta_{ij}^t \in [-\pi/2, \pi/2]$ is the zenith angle, and $\phi_{ij}^t \in [-\pi, \pi]$ is the azimuth angle (Supplementary Figure 24b).

(iii)Projection of relative position of neighbor-j on $\theta - \phi$ plane based on $(r_{ij}^t, \theta_{ij}^t, \phi_{ij}^t)$. In Supplementary Figure 24c, $\theta - \phi$ plane is divided into 8 relative directions from the view of the focal individual-i. Note that we only care about the front view, that is, only focused on the range of ϕ_{ij} no matter what θ_{ij} is.

Therefore, at time t, $\mathbf{x}_j(t)$ equals a point in $\theta - \phi$ plane (Supplementary Figure 24c). For a flock, collecting the whole time, the trajectories could be converted to curves in $\theta - \phi$ plane. For example, Supplementary Figure 24d,e show the continuous relative positions projecting to $\theta - \phi$ plane from "Bird-6 sees Bird-1" and "Bird-1 sees Bird-6". For a flock shown in Fig.1a, Supplementary Figure 24f shows the neighbors' relative positions projecting on $\theta - \phi$ plane from the view of the focal bird.

Second, for a given range of front view, such as π in Supplementary Figure 24f, we calculate

$$\text{fraction of neighbors present in front view} = \frac{\#\text{ of points on } \theta-\phi \text{ plane within } \phi\in[-\pi/2,\pi/2]}{\#\text{ of all points on } \theta-\phi \text{ plane}} \in [0,1].$$

Supplementary Figure 24f displays the histogram of neighbors present in the azimuth view as a function of ϕ from $-\pi$ to π. Interestingly, we find that, consider the whole time of the flock, there are less neighbors appeared in the front view for the individual with higher L_i, that is, the leaders always locate at the front in the flock. For example, bird-1 with the highest leading tier ($L_1 = 1$) only has 0% neighbors accumulatively present in the front view of $[-\pi/2, \pi/2]$ over the whole time of the flock, while the fraction is 80% for bird-6 with the lowest leading tier ($L_6 = 0$). This phenomenon of leaders always being at the front of this flock accords with the observation in Supplementary Movie 4.

Finally, we extend the analysis of spatial structure of leading position to three flocking datasets. Supplementary Figure 25 demonstrates that the mobbing and circling flocks show the falling trend between leading tier and fraction of neighbors in front view, while the trend in transit flocks is flat no matter what the range of front view is π or $2\pi/3$. The results demonstrate that the flocks with maneuverable motions (e.g., mobbing and circling) require individuals in the higher leading tier to be located at the front of flock. The transit flocks do not show any preference between leading tier and neighbors' relative position. These phenomena accord with the findings in mobbing and circling flocks that the domination of positive correlation between LF and MS occurred in the situation of forward-oriented preference of biological perception ($\alpha > 0$).

7. Correlation analysis of MS-Speed and LF-Speed

Given the well-known finding that faster individuals tend to assume leadership positions in animal groups[10], two natural concerns arise regarding the correlation of MS-Speed and comparison of correlation between LF-MS and LF-Speed. Here the "Speed" of each individual are three types: average speed, average radial speed and average angular speed.

7.1 Statistics of bird's average speed, radial and angular speed of three flocking datasets

To investigate the relations between MS and Speed, we first count each bird's average speed, average radial speed and average angular speed. Note that each bird's average speed, radial and angular speed is calculated over the entire duration of a flock. For mobbing, circling and transit datasets, Supplementary Figure 28 shows three speeds of 3103, 75683, and 2597 birds, respectively. We find that the average speed and radial speed of transit dataset is significantly larger than that of mobbing and circling datasets, and they are quite similar to each other for mobbing and circling datasets. The average angular speed of flocks, in descending order, is observed to be highest in mobbing flocks, followed by circling flocks, and finally transit flocks. This order aligns with the observed motion patterns exhibited by these types of flocks.

7.2 Correlation between MS and three kinds of speed

In addition to the correlation analysis between MS and LF shown in Fig.3a, we also conduct a correlation analysis of two vectors from MS and three types of speed for a given period $[t - \tau, t]$ within a flock (see workflow in Supplementary Figure 29a-c). Consider the entire flock, we calculate the Spearman correlation (ρ) for various combinations of (t, τ) to assess their level of

correlation (Supplementary Figure 29d). Next we extend the correlation analysis to three datasets and yielded the distribution of $\rho_{\mathrm{MS-Speed}}(t,\tau)$, $\rho_{\mathrm{MS-Radial}}(t,\tau)$ and $\rho_{\mathrm{MS-Angular}}(t,\tau)$ (Supplementary Figure 29e,f). In order to facilitate a clear comparison of the correlation levels between MS-LF (as shown in Fig.3 of the main text) and three types of MS-Speed, Supplementary Figure 30 presents boxplots of $\rho_{\mathrm{MS-LF}}(t,\tau)$, $\rho_{\mathrm{MS-Speed}}(t,\tau)$, $\rho_{\mathrm{MS-Radial}}(t,\tau)$ and $\rho_{\mathrm{MS-Angular}}(t,\tau)$. These boxplots provide a visual representation of the correlation strengths for the different combinations of (t,τ) values. Interestingly, regardless of three flocking datasets and α values in MS, the Spearman correlation for the three types of MS-Speed does not demonstrate a significant positive or negative dominance (Supplementary Figure 30). This suggests that the correlations between these variables are not consistently biased towards either positive or negative values across different scenarios. In particular, when $\alpha = 1$, $\rho_{\mathrm{MS-LF}}$ exhibits predominantly positive values in mobbing and transit flocks. However, this phenomenon could not be observed in the correlation of the three types of MS-Speed, as depicted in Supplementary Figure 30b1 and b2.

7.3 Comparison of correlation of LF-MS and LF-Speed

Regarding the comparison of correlation between LF-MS and LF-Speed, we use a mobbing flock (shown in Fig.1a) to show the workflow of comparison:

(i) First, performing the Spearman correlation to yield $\rho_{\mathrm{LF-MS}}(t,\tau)$ and $\rho_{\mathrm{LF-Speed}}(t,\tau)$, $\rho_{\mathrm{LF-Radial}}(t,\tau)$, $\rho_{\mathrm{LF-Angular}}(t,\tau)$ for different combinations of (t,τ). In Supplementary Figure 31a-c, each panel contains 231 points corresponding to 231 combinations of (t,τ) within the mobbing flock.

(ii) Second, scatter-plotting of $\rho_{\mathrm{LF-MS}}(t,\tau)$ and $\rho_{\mathrm{LF-Speed}}(t,\tau)$ (or $\rho_{\mathrm{LF-Radial}}(t,\tau)$ or $\rho_{\mathrm{LF-Angular}}(t,\tau)$), and calculating the difference of ρ as $\Delta\rho(t,\tau) = \rho_{\mathrm{LF-MS}}(t,\tau) - \rho_{\mathrm{LF-Speed}}(t,\tau)$, $\rho_{\mathrm{LF-MS}}(t,\tau) - \rho_{\mathrm{LF-Radial}}(t,\tau)$, $\rho_{\mathrm{LF-MS}}(t,\tau) - \rho_{\mathrm{LF-Angular}}(t,\tau)$. The scatter plot of ρ and heatmap of $\Delta\rho$ for the mobbing flock are shown in Supplementary Figure 31a-c and Supplementary Figure 31d-f. The gradient color ranging from red to white to blue in Supplementary Figure 31d-f is indicative of the value of $\Delta\rho \in [-2,2]$. To clearly demonstrate $\Delta\rho$ in different combinations of (t,τ), Supplementary Figure 31g shows the flocking trajectory from $[t-\tau,t]$ with the gradient background color scaling with $\Delta\rho = \rho_{\mathrm{LF-MS}}(t,\tau) - \rho_{\mathrm{LF-Speed}}(t,\tau)$. We observe that the $\Delta\rho(t,\tau)$ values indicated by the red background color consistently correspond to the process of collective turn.

(iii) Finally, classifying $\Delta\rho$ from all the combinations of (t,τ). For example, in Supplementary Figure 31h-j**,** the 231 points are categorized into 3 parts: when $|\Delta\rho| < 0.2$, it signifies that the two correlations are very similar; $\Delta\rho$ exceeding (or falling below) a specific threshold indicates that the $\rho_{\mathrm{LF-MS}}\ (t,\tau)$ is noticeably greater than (or less than) the LF-Speed correlation. Besides, y-axis of Supplementary Figure 31h-j records the corresponding average angular and nestedness. Interestingly, for $\Delta\rho(t,\tau) = \rho_{\mathrm{LF-MS}}(t,\tau) - \rho_{\mathrm{LF-Speed}}(t,\tau)$ and $\rho_{\mathrm{LF-MS}}(t,\tau) - \rho_{\mathrm{LF-Angular}}(t,\tau)$ shown in

Supplementary Figure 31h,j, the nestedness of those sub-flocks with $\Delta\rho > 0.5$ (the red background color in Supplementary Figure 31g) is the highest, while the nestedness of sub-flocks with $\Delta\rho < -0.5$ (the blue background color in Supplementary Figure 31g) is the lowest. This trend is the same with average angular speed.

In the mobbing flock, we observe a clear pattern where the sub-flocks with $\rho_{\mathrm{LF-MS}}(t,\tau) - \rho_{\mathrm{LF-Speed}}(t,\tau) > 0.5$ (or $\rho_{\mathrm{LF-MS}}(t,\tau) - \rho_{\mathrm{LF-Angular}}(t,\tau) > 0.5$) consistently exhibit the highest nestedness, while those sub-flocks with $\Delta\rho < -0.5$ display the lowest nestedness. We hypothesize that this phenomenon may be related to the collective motion patterns. Consequently, we replicate the same analysis process to a transit flock as illustrated in Fig.1c. In Supplementary Figure 32, we find three kinds of $\Delta\rho$ is significantly lower than that of mobbing flock, because the color intensity in Supplementary Figure 32d-f is noticeably lighter compared to that in Supplementary Figure 31d-f**.** And the nestedness value of $\Delta\rho > 0.5$ and $\Delta\rho < -0.5$ consistently remains at a relatively low level (Supplementary Figure 32h-j). Based on these two examples with distinct motion patterns, we hypothesize that this phenomenon of different $\Delta\rho$ corresponding to different flocking properties may be associated with the collective motion patterns.

Furthermore, we extend the workflow to mobbing, circling and transit datasets (Supplementary Figure 33). In mobbing and circling dataset, the sub-flocks with $\Delta\rho > 1$ exhibit the highest nestedness, while those sub-flocks with $\Delta\rho < -1$ display the lowest nestedness. The nestedness of sub-flocks with $|\Delta\rho| < 0.2$ are positioned in the middle. Note that in Supplementary Figure 33 the trend is the same for three kinds of $\Delta\rho$. For the transit dataset, we could not observe the clear pattern between $\Delta\rho > 1$ and $\Delta\rho < -1$. The validation with three large datasets confirms our hypothesis. The flock (or sub-flock) with maneuverable motions (such as mobbing and circling flocks), characterized by higher angular speed and nestedness, exhibits a notably stronger positive correlation between LF and MS when compared to the correlation between LF and Speed. Conversely, the correlation between LF and Speed is significantly greater than that between LF and MS for the flock with lower angular speed and nestedness (e.g., the transit flocks in this work). In summary, LF-MS may be more effective in describing the flocks with maneuverable motions, whereas LF-Speed is better suited for characterizing flocks with smooth motions. The difference between MS and Speed in this analysis could mainly be attributed to the scope of their respective definitions. From the definition, MS directly or indirectly takes into account the variations in position, heading, speed and acceleration over a period of flock. Three kinds of speed serve as scalar representation of collective motions. It is evident that speed, when compared to MS, is not a suitable description of the collective motion modes, especially for extremely agile flocks.

8. The effectiveness of leadership in AMS interaction

To further investigate the effectiveness of leadership in the flocking models, we incorporate a tunable parameter to the simulation results of Fig.5: the number of individuals leading the group. Supplementary Figure 39 shows the curves of average curvature of flocking trajectory and nestedness with the increment of the number of individuals leading the group.

The results from Supplementary Figure 39a indicates that:

- three interactions used in swarm models show the increment of average curvature of flocking tracks as the leading individuals in the group increases.
- with an increasing number of individuals leading the group, the average curvature of flocking trajectories for ATHD and Average approaches that of AMS.

If we recognize the average curvature as a kind of performance for the simulation setting of Fig.4, the augmentation of leading individuals enhances group performance; however, despite this improvement, AMS remains the most effective, while the performance of Average and ATHD only exhibit a tendency towards convergence with AMS.

For the results of nestedness, Supplementary Figure 39b indicates that:

- when the number of leading individuals in a group becomes excessive, it results in a reduction in the nestedness of LF networks. This observation aligns with our intuitive understanding that an abundance of leading individuals within a group diminishes the unambiguity of hierarchical relationships in the LF networks.
- Interestingly, in Average and ATHD, the increment of leading individuals initially results in an increase in the nestedness of LF networks, followed by a subsequent decrease. This suggests that in the case of these two interactions, a smaller addition of leaders could potentially enhance the hierarchical structure of LF networks.
- Compared with another two interactions, even with a small number of leading individuals, AMS could maintain a high level of nestedness and a clearer hierarchical structure of LF network.

Overall, the findings shown in Supplementary Figure 39 indicate that AMS not only facilitates the effective leadership with a smaller number of informed individuals but also plays a crucial role in the emergence of hierarchical structures in LF networks.

9. Experimental set-up of swarm robotics

In Fig.5g,h and Supplementary Figure 38**,** the simulation results show that regardless of the absence ($\alpha = 0$) or presence ($\alpha = 1$) of blind area, there are consistently positive correlations dominating the relation between MS and LF. However, in the real bird flocks, the positive correlations depend on $\alpha > 0$. This difference in simulations and empirical data analysis could be attributed to that: the flight status, locomotor ability, and flight dynamics of birds impose constraints that prevent them from achieving a perception free of blind spots. As a result, birds exhibit a preference for forward-oriented perception, and Ref.[10] reported a tendency for leaders to fly at the front of the flock. Swarm simulations and miniature two-wheel differential mobile robots utilized in swarm robotics applications often have minimal or no movement restrictions, providing increased flexibility and freedom of motion. Thus, we ignore to consider the blind area of perception and set $\alpha = 0$ in the collective following and collective evacuation experiments.

9.1 Swarm model of collective following

The collective following experiments aim to simulate the ability of animal flocks to flexibly change the global motion direction due to the observed external stimuli (e.g., food, obstacles, or predators) while maintaining group cohesion. Supposing that there exists an informed agent to move towards the multiple destinations successively, and the rest try to keep group cohesion through the interaction rule to align with each other (Supplementary Figure 47).

To generate the wide range of motion changes for the informed robot, we set 10 targets with living, sleeping, or arrived states independently as following:

(i) at the beginning of experiments, 10 targets are randomly distributed in the experiment arena;

(ii) each non-arrived target has a probability of being seen by the informed robot. If a random number generated from $U(0,1)$ is less than 0.2, the living state and a corresponding survival value that an integer is generated from $U[1,20]$ are assigned to the target;

(iii) the informed agent only moves to the nearest living target;

(iv) after a step passed, the survival value of each living target reduces by 1. Until the survival value decreases to 0, the living target transfers to the sleeping state;

(v) the sleeping target will repeat step (ii) at each step until it becomes alive;

(vi) if the distance between informed agent and the nearest target is less than 80mm (about equals body length of our robot), the *arrived* state is assigned to the target permanently.

Due to the randomness and frequent switch between living and sleeping state in the multiple targets, we could generate a wide range of change rates in headings of the informed robot to test the performance of collective following.

Except the movement of the informed robot is governed by the above rules, the rest of swarm follow the model as following:

$$\mathbf{v}_i(t + \Delta t) = k_{\mathrm{al}}\hat{\mathbf{v}}_{\mathrm{al},i} + (1 - k_{\mathrm{al}})\hat{\mathbf{v}}^{a}_{\mathrm{rep},i}. \qquad . \qquad [5]$$

Here $\hat{\mathbf{v}}_{\mathrm{al},i}$, the alignment part, indicates the alignment to maintain group cohesion in response to stimuli from the informed robot. k_{al} is the alignment coefficient. The alignment exerted on agent-i is the weighted average of neighbor-j's velocity,

$$\mathbf{v}_{\mathrm{al},i} = \hat{\mathbf{v}}_i + \sum_{j \in \mathcal{S}_{\mathrm{al},i}} w_{ij}\hat{\mathbf{v}}_j \quad \text{if } d_{ij} < r_{\mathrm{al}} \qquad [6]$$

w_{ij}, calculated by the perception of MS, denotes the MS-based adaptive interaction. If $w_{ij} = 1$, it reduces to the average interaction.

$\hat{\mathbf{v}}^{a}_{\mathrm{rep},i}$ represents the repulsion among inter-agent to push them farther apart when the neighbors are closer than the pre-defined distance d^{a}_{rep}. The repulsion term pushes the near neighbor j farther apart as below,

$$\mathbf{v}^{a}_{\mathrm{rep},ij} = \begin{cases} \left(d^{a}_{\mathrm{rep}} - d_{ij}\right)\dfrac{\mathbf{x}_i - \mathbf{x}_j}{d_{ij}} & \text{if } d_{ij} < d^{a}_{\mathrm{rep}} \\ \mathbf{0} & \text{otherwise} \end{cases}. \qquad [7]$$

The total repulsion calculated for agent i with respect to the repulsive set $\mathcal{S}_{\text{rep},i} = \{j | d_{ij} < d^a_{\text{rep}}\}$ is $\mathbf{v}^a_{\text{rep},i} = \sum_{j \in \mathcal{S}_{\text{rep},i}} \mathbf{v}^a_{\text{rep},ij}$. All the parameters used in collective following experiments and simulations are listed in Supplementary Table 1.

In the collective following experiments, we evaluate the collective response triggered by the new moving directions of informed robot. Suppose that if the informed agent points towards a new direction $\hat{\mathbf{n}}(t)$ at time t, the flock starts to respond this new directional information and the transient collective response could be evaluated by

$$r(t) = \hat{\mathbf{v}}(t) \cdot \hat{\mathbf{n}}(t) \in [-1,1],$$

where $\hat{\mathbf{v}}(t) = 1/N \sum_i \hat{\mathbf{v}}_i(t)$ for all robots. Over the duration time of collective response from t_0 to t_1, the cumulative evaluation of collective response is

$$R = \frac{1}{t_1 - t_0} \int_{t_0}^{t_1} 1 - r(t)\, dt.$$

Note that the values of R are mathematically bound between 0 and 2. $R = 0$ means the immediate collective response that all the neighbors copy the new velocity $\hat{\mathbf{n}}(t)$ without any delay during the whole process, while $R = 2$ indicates the worst response that the flock moves the opposite direction of the informed robot.

9.2 Swarm experiments of collective following

Collective following experiments aim to mimic the ability to respond with agility to rapid changes of moving directions while maintaining the group cohesion, subject to the perturbations from external stimuli or interior neighbors. The inspiration of experimental set-up comes from collective foraging, for instance, an informed individual leads the group to the multiple destinations that frequently changes during foraging[12,13]. In the collective following experiments, we suppose that one of the swarm acts the informed role to move towards the nearest target, and the rest align with neighbors through AMS. To simulate the rapid motion changes for the informed robot, we artificially set 10 targets to guide the moving direction of informed robot through the switch among three different states: living, sleeping and arrived. See Supplementary Note 9.1 for detailed information about how the informed robot moves according to the target states. Besides, we use $r(t) \in [-1,1]$ to measure the temporal collective response of a flock to rapid motion changes of the informed robot. $r(t) = 1$ means the perfect response that the swarm could ideally copy the velocity of informed robot at the moment t, while $r(t) = -1$ indicates the worst response that the rest of swarm move oppositely to the informed robot. We also use $R = \frac{1}{t_1 - t_0} \int_{t_0}^{t_1} 1 - r(t)\, dt \in [0,2]$ to cumulatively measure the general performance of collective response for a period. The less R, the better collective response.

The experimental results demonstrate that, using AMS interactions, the swarm with 50 robots not only successfully follow the informed robot to reach 10 targets in sequence (Supplementary Figure 48a and Supplementary Movie 9), but also quickly respond to the motion changes of informed robot (Supplementary Figure 48b). When the informed robot drastically changes its heading towards the nearest living target, the swarm could speedily recover to the consensus state

(see the sudden drops and meteoric rises of $r(t)$ in Supplementary Figure 48c). As a comparison, the swarm using average interaction totally fails to follow the informed robot (Supplementary Figure 48d-f and Supplementary Movie 11). Supplementary Figure 48g demonstrates that when the flock size is single-digit (< 10), there is little difference in R for the interaction rule in the presence or absence of MS. However, AMS interactions show a strong advantage in keeping R stabilized at a low level ($R \approx 0.1$ in Supplementary Figure 48g), no matter what the flock size increases from 10 to 50 robots in real experiments. In addition to the experimental verification of collective following, a simulation platform is developed with the same robot's physical characteristics. Even if the flock size grows up to 100 in simulations, R is still stable at around 0.1 (Supplementary Figure 48h).

To further reflect the greater attention given to neighbors with higher MS, we introduce a tunable parameter $x\%$ to AMS interactions: the focal individual only aligns with its neighbors who cumulatively possess the top $x\%$ MS. Both the real experiment and simulation results demonstrate that even if just the top 20% or 50% MS is involved, the collective response performs almost the same as that used 100% MS (Supplementary Figure 48g,h). Besides, we systematically investigate the collective response as a function of the top $x\%$ MS used in AMS interactions. It indicates that the smallest R occurs between the top 20% and 40% MS, and R slightly increases with the increment of the value of $x\%$ (Supplementary Figure 49), which coincidences with the theoretical modeling of selective attention through limiting the cognitive capacity of individuals to maximize the flocking performance[14,15,16].

On the other hand, we also compare the performance of AMS with ATHD in the collective following experiments. ATHD could be considered a theoretically ideal response to neighbors' perturbations, because it could adaptively alter the heading difference with the leader and does not involve any delay. Regarding AMS, it encompasses adaptive influences on a neighbor's subsequent decision, with the adaptive coefficient being derived from a period of τ. Clearly, while a smaller τ leads to a smaller R in the collective following experiments, it also leads to an extended evacuation time for the swarm in the next experiments about collective evacuation. We discuss the effect of perceiving time τ on AMS in Supplementary Note 9.3. Interestingly, despite AMS taking into account a delay of τ, the collective following experiments show that the collective response of AMS interactions closely approximates that of ATHD interactions (Supplementary Figure 48g,h). This demonstrates the advantage of AMS in responding to transient perturbations.

Furthermore, we consider the scenario of restricted angular velocity with two kinds of collective following simulations: (i) the informed agent and others share the same max angular velocity (Supplementary Figure 50a); (ii) max angular velocity of the others is fixed at 10*1.91 rad/s but we change the informed agent with different max angular velocity (Supplementary Figure 50b). Note that as the miniature mobile robot is constrained by the performance of the motor and battery, the maximum angular rate $\omega_{\max} = k * 1.91\ \mathrm{rad/s}$ and k is an integer number between 1 and 10. From two kinds of collective following simulation results with restricted angular velocity, we find that:

- For the informed agent and others share the same max angular velocity (Supplementary Figure 50a), the performance of collective response (R) does not change no matter what the max angular velocity is high or low. The factor that exerts the greatest influence on performance is still the flock size.
- If max angular velocity of the others is fixed at $10*1.91$ rad/s but we increase max angular velocity of the informed agent from $2*1.91$ to $10*1.91$ (see x-axis in Supplementary Figure 50b), the R value indicates very slight increment for AMS and ATHD, but the increment is obvious for Average especially when the flock size is large (e.g., $N = 70,90$). Overall, AMS and ATHD exhibit insensitivity to variations in the max angular velocity between leaders and the others in the group. This implies that regardless of whether the difference in maximum angular velocity between leaders and others is larger or smaller, the collective response performance from AMS and ATHD stabilizes at a good level. However, the performance from Average interaction deteriorates progressively as the difference in maximum angular velocity between leaders and the rest of the flock decreases.

Of course, the above findings only work when the group size is larger than 30 (Supplementary Figure 50). When the flock size is less than 20, the performance from three kinds of swarm interactions displays no difference. Hence, the simulations of collective following with constrained angular velocity reaffirm the robustness of AMS in ensuring optimal performance in collective response.

9.3 The effect of perceiving time (τ) on AMS

According to the results of collective following and collective evacuation experiments, we find that AMS not only empowers the swarm to promptly respond the transient perturbation, but also strengths the self-organization of collective motions in terms of temporal cognition. Therefore, we investigate the effect of perceiving time (τ) on AMS.

In Fig.4 of the main text, we use AMS with $\tau = 20\Delta t$ to generate the flocking trajectories. Same with other parameters in Fig.4, Supplementary Figure 51a shows the nestedness of LF networks as a function of τ. The results clearly demonstrate the increment of nestedness as τ increases, that is, the longer perceiving time could induce more nested LF relations in the flocks.

Besides, we run the simulations of collective following with different τ to evaluate the collective response. Supplementary Figure 51b shows that R increases with the increment of τ. It is because that the longer perceiving time τ in AMS could weaken the performance of collective response to transient perturbation. However, in the simulations of collective evacuation, Supplementary Figure 51c shows that the spending time for all the swarm passing the narrow exit first deceases with the increment of τ, and then keeps the stable.

Overall, the results demonstrate that the temporal cognition in collective tasks should perceive a period of time to strength the self-organization in collective motions, while the long perceiving time could weaken the collective response to transient perturbation. This phenomenon suggests an

open question of how the real flocks balance the perceiving time to take advantages in coping with both transient perturbation and temporal cognition.

Supplementary Tables

Supplementary Table 1 | Parameters used in the experiments and simulations of collective following and collective evacuation. Note that due to the fact that the simulation platform is developed based on the robot's physical characteristic, the parameters are the same in the experiments and simulations.

**Parameters**	**Symbol**	**Collective evacuation**	**Collective following**	**Unit**
time interval of each step	Δt	0.2	0.5	second (s)
perceiving time in MS	τ	$10\Delta t$	$10\Delta t$	second (s)
anisotropic factor of individual perception	α	0	0	/
normal speed	v_0	15	15	Millimeter/second (mm/s)
speed to avoid collision against inter-agent or agent-wall	v_{col}	2	/	Millimeter/second (mm/s)
alignment coefficient	k_{al}	/	0.2	/
body length of the robot	BL_b	60	60	millimeter (mm)
body length of the deck on top of robot	BL_d	84	84	millimeter (mm)
diameter of wall	D_w	50	/	millimeter (mm)
sensing radius	r_{al}	inf	50BL_b	millimeter (mm)
repulsion distance among agents	d_{rep}^a	1.5BL_d	2.5BL_b	millimeter (mm)
distance of inter-agent collision avoidance	d_{col}^a	1.25BL_d	/	millimeter (mm)
distance of agent-wall collision avoidance	d_{col}^w	$1.2\left(\frac{\text{BL}_d}{2}+\frac{D_w}{2}\right)$	/	millimeter (mm)
radius to get the guidance velocity	r_g	$10D_w$	/	millimeter (mm)
distance between two agents	d_{ij}	calculated	calculated	millimeter (mm)
distance between agent i and the nearest wall	d_i^w	calculated	/	millimeter (mm)
distance between agent i and wall k	d_{ik}^w	calculated	/	millimeter (mm)

Supplementary Table 2 | The descriptions of terms or notions used in the data processing.

Terms/Notions	Descriptions
frame	equals the minimal time stamp of the original video recordings;<br>equals a column of individual present matrix or frame matrix.
frame rate	the minimal time interval of the original video recordings. The frame rate is 60 frame/s for mobbing and transit datasets, and 30 frame/s for circling dataset.
tracks_filt matrix	stores the movement information about absolute (x, y, z)-position and corresponding (v_x, v_y, v_z)-velocity for individuals at time stamp t.
individual present matrix	the rows indicate the individuals and the columns represent the time stamps. The time stamp equals the frame in a video recording. If bird-i is present at time t, the element (i, t) in the individual present matrix stores the row number of tracks_filt matrix to map the movement information of bird-i at time t.
frame matrix	It is a kind of individual present matrix to represent a period of continuous flocking trajectories.

Supplementary Figures

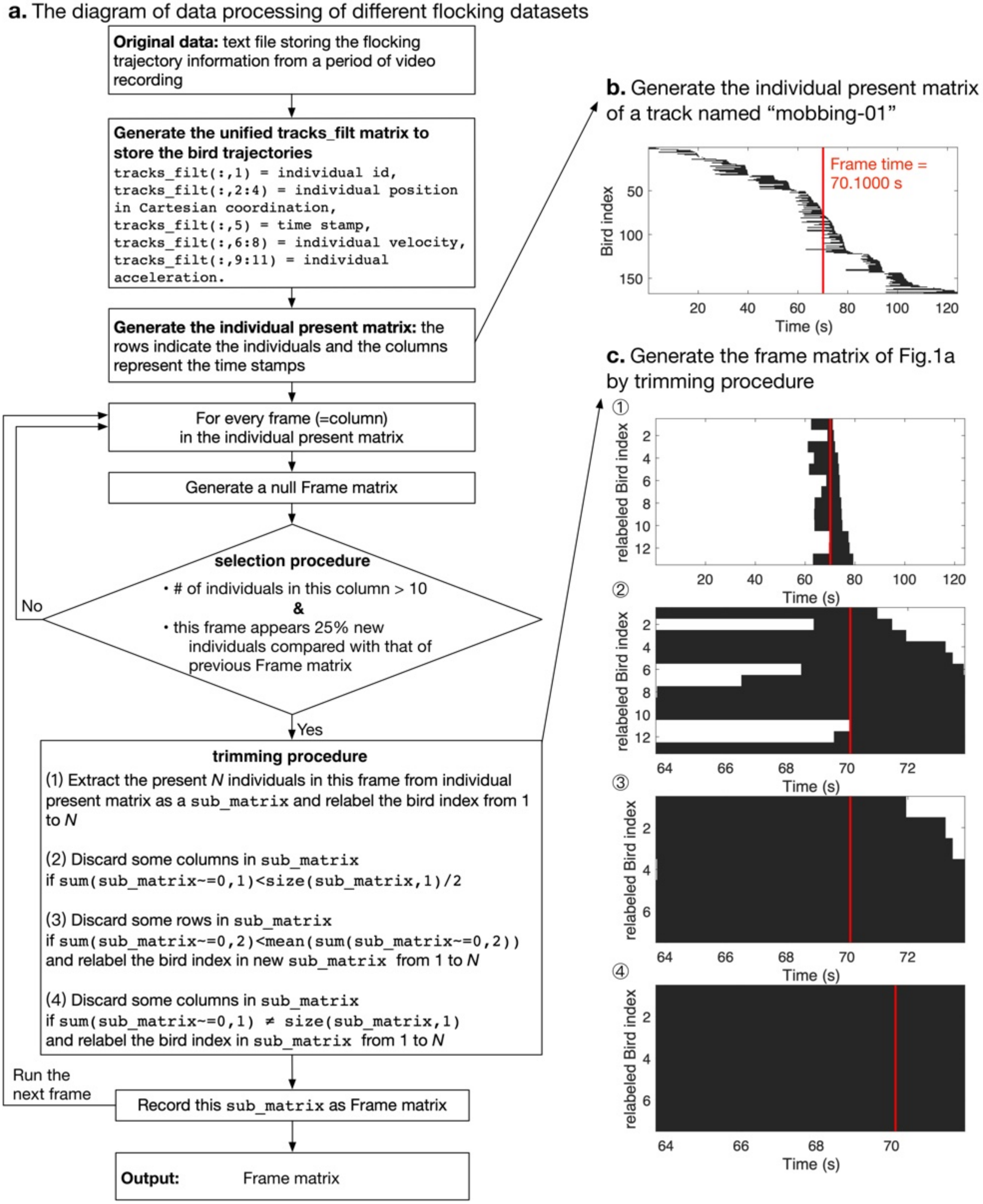

Supplementary Figure 1 | Workflow of data processing for three bird flocking datasets. a, Diagram of data processing to yield many periods of continuous flocking trajectories from the original whole video recording. **b,** For a given frame from the original track of "mobbing-01", our workflow first generated the individual present matrix where the rows indicate the individuals and the columns represent the time stamp (equals the frame in the video recording). The black area means an individual-i is recorded at time t, and the blank area indicates none of birds is recorded at time t. The red line highlights a frame (also equals a column) in the individual present matrix, which will be used to generate the frame matrix in the next step. **c,** It shows 4 steps of trimming procedure to generate the final frame matrix corresponding to a mobbing flock (shown in Fig.1a of the main text).

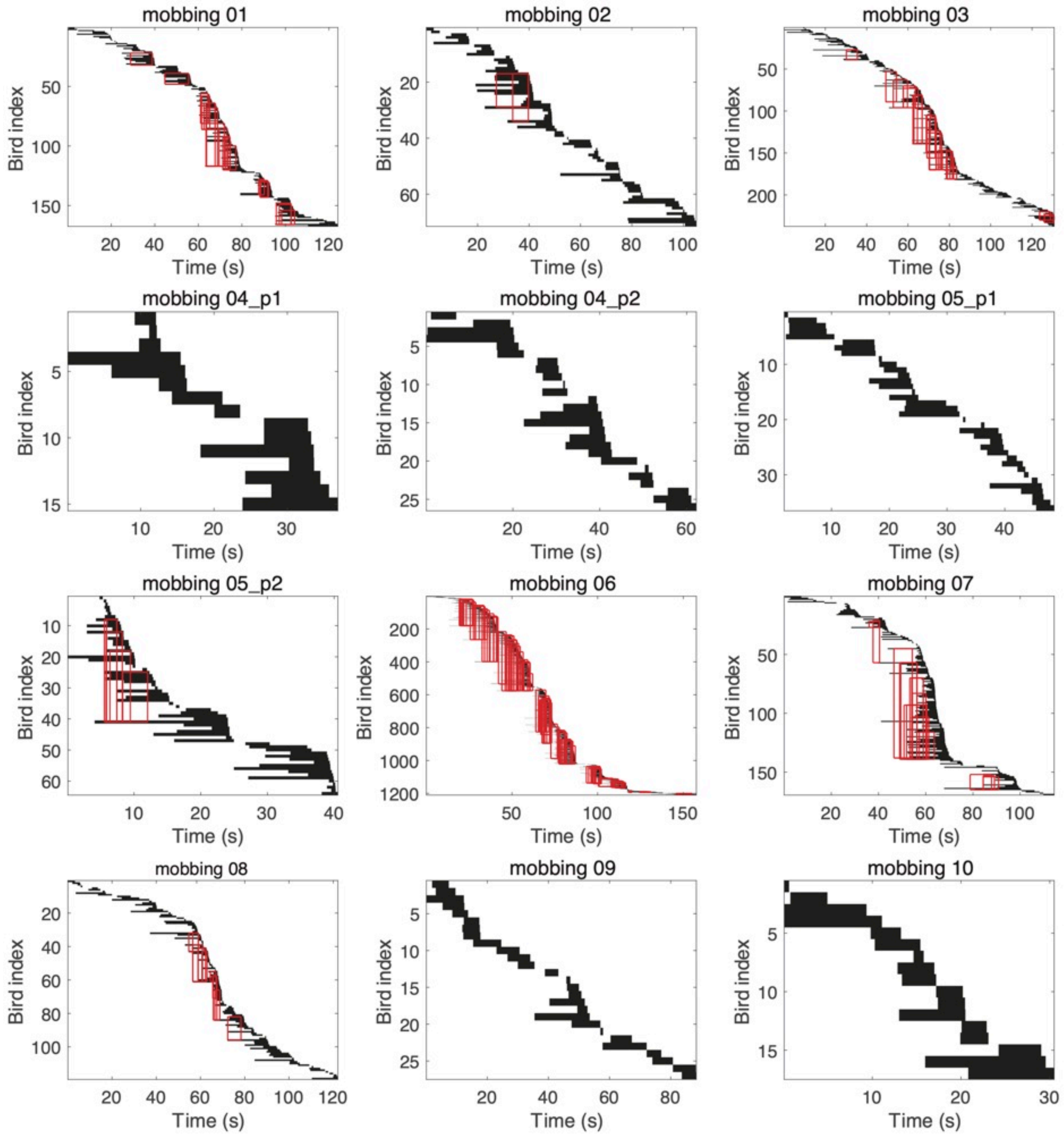

Supplementary Figure 2 | The individual present matrix of the original mobbing dataset. In the original mobbing dataset[1], there are total 12 tracks of video recordings. The resolution of each video recording is 60 frame/s. The elements in the individual present matrix store the row number of corresponding tracks_filt if an individual-i is recorded at time t. After running our workflow of data processing, each individual present matrix could generate multiple frame matrices as highlighted by red boxes. If there does not exist the red box in an individual present matrix, it means this track does not meet the requirements of selection and trimming procedure demonstrated in step (iii) of our workflow.

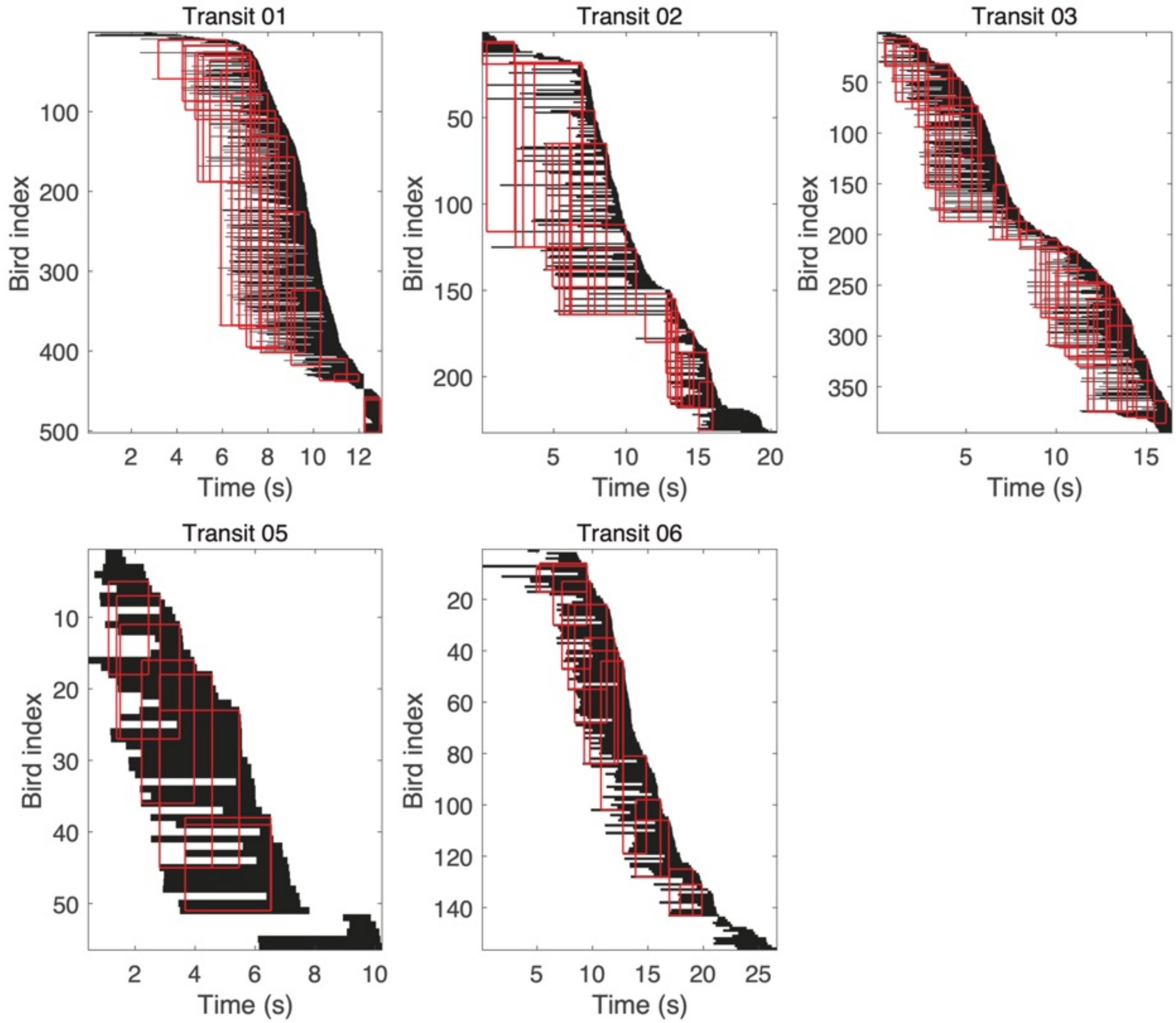

Supplementary Figure 3 | The individual present matrix of the original transit dataset. In the original transit dataset[1], there are total 6 tracks. Due to the same recording between transit-03 and transit-04, we just analyze 5 video recordings. The resolution of each video recording is 60 frame/s. The elements in the individual present matrix store the row number of corresponding tracks_filt if an individual-i is recorded at time t. After running our workflow of data processing, each individual present matrix could generate multiple frame matrices as highlighted by red boxes.

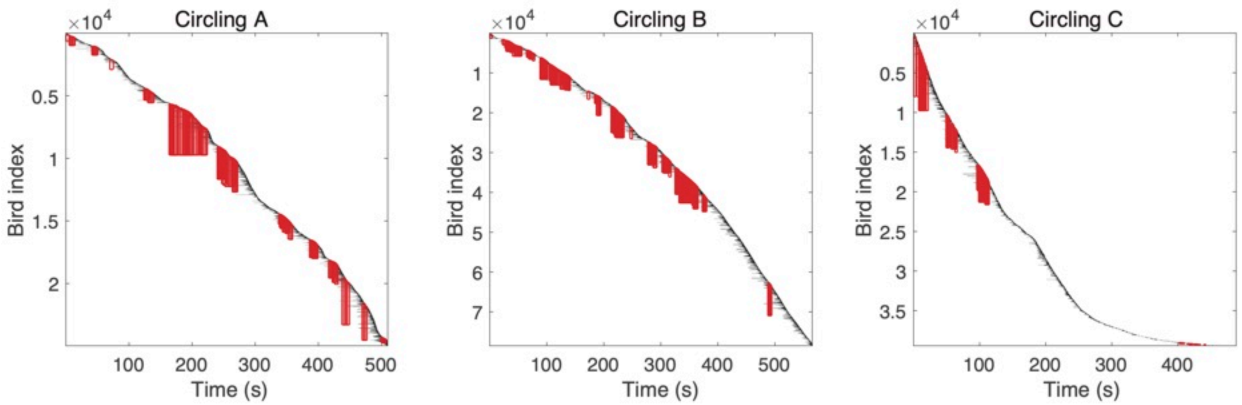

Supplementary Figure 4 | The individual present matrix of the original circling dataset. In the original circling dataset[2], there are total 3 tracks with much longer duration of recording time compared with mobbing and transit datasets. The resolution of each video recording is 30 frame/s. The elements in the individual present matrix store the row number of corresponding tracks_filt if an individual-i is recorded at time t. After running our workflow of data processing, each individual present matrix could generate multiple frame matrices as highlighted by red boxes.

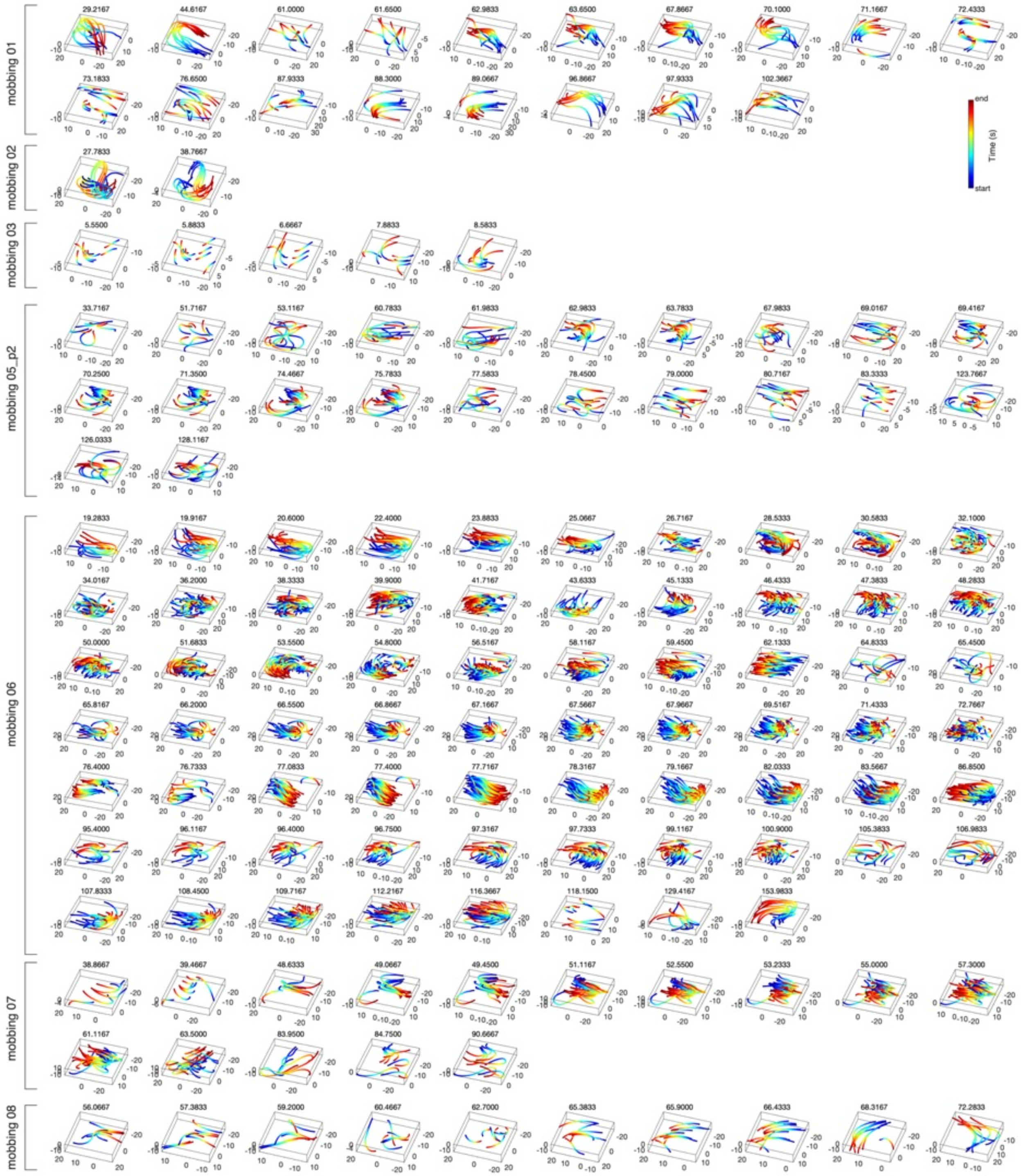

Supplementary Figure 5 | The short periods of continuous tracks of mobbing flocks yielded by our workflow of data processing. Our workflow generates 140 short periods of continuous tracks from the original mobbing dataset. Each 3D panel corresponds to a frame matrix. For example, the video recording of "mobbing-01" yielded 18 frame matrices meeting the requirements of selection and trimming procedure in step (iii) of our workflow. The top number in each panel indicates the frame time to generate the frame matrix. The gradient color from blue to red maps the flocking time from beginning to end.

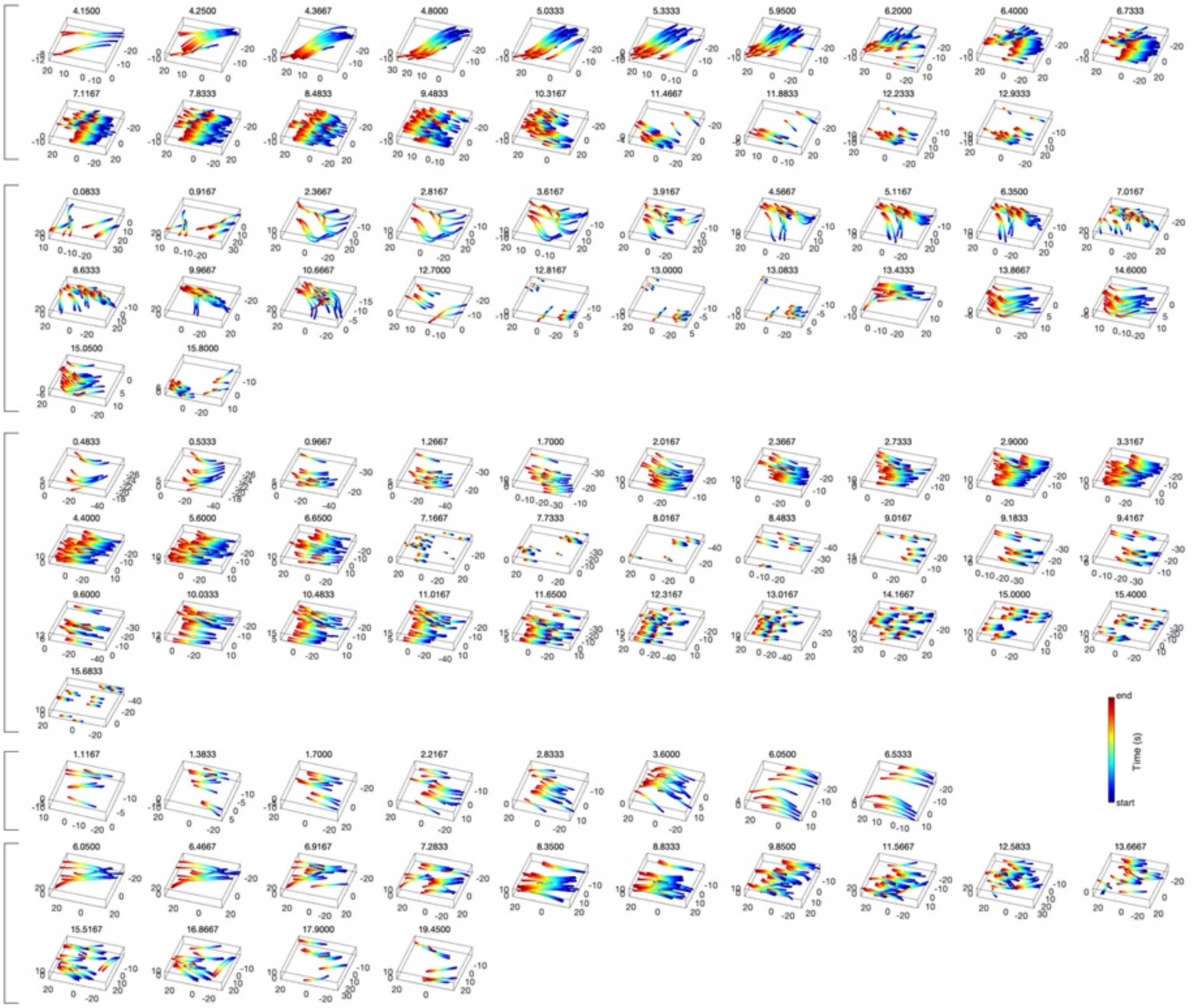

Supplementary Figure 6 | The short periods of continuous tracks of transit flocks yielded by our workflow of data processing. Our workflow generates 94 short periods of continuous tracks from the original transit dataset. Each 3D panel correspond to a frame matrix. The top number in each panel indicates the frame time to generate the frame matrix. The gradient color from blue to red maps the flocking time from beginning to end.

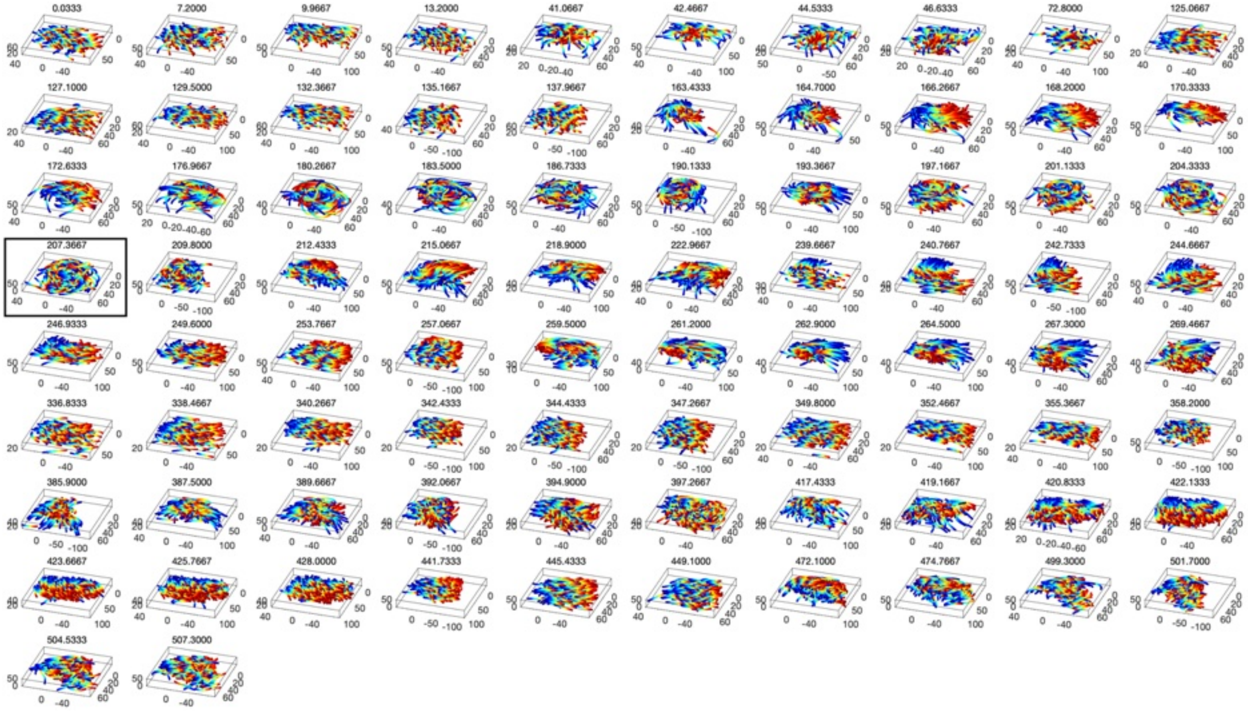

Supplementary Figure 7 | The short periods of continuous tracks of "circling A" flocks yielded by our workflow of data processing. Our workflow generates 82 short periods of continuous tracks from the original "circling A" dataset. Each 3D panel corresponds to a frame matrix. The top number in each panel indicates the frame time to generate the frame matrix. The gradient color from blue to red maps the flocking time from beginning to end. The black box highlights a circling flock to show how to classify the sub-communities in Supplementary Figure 10 and Supplementary Movie 2.

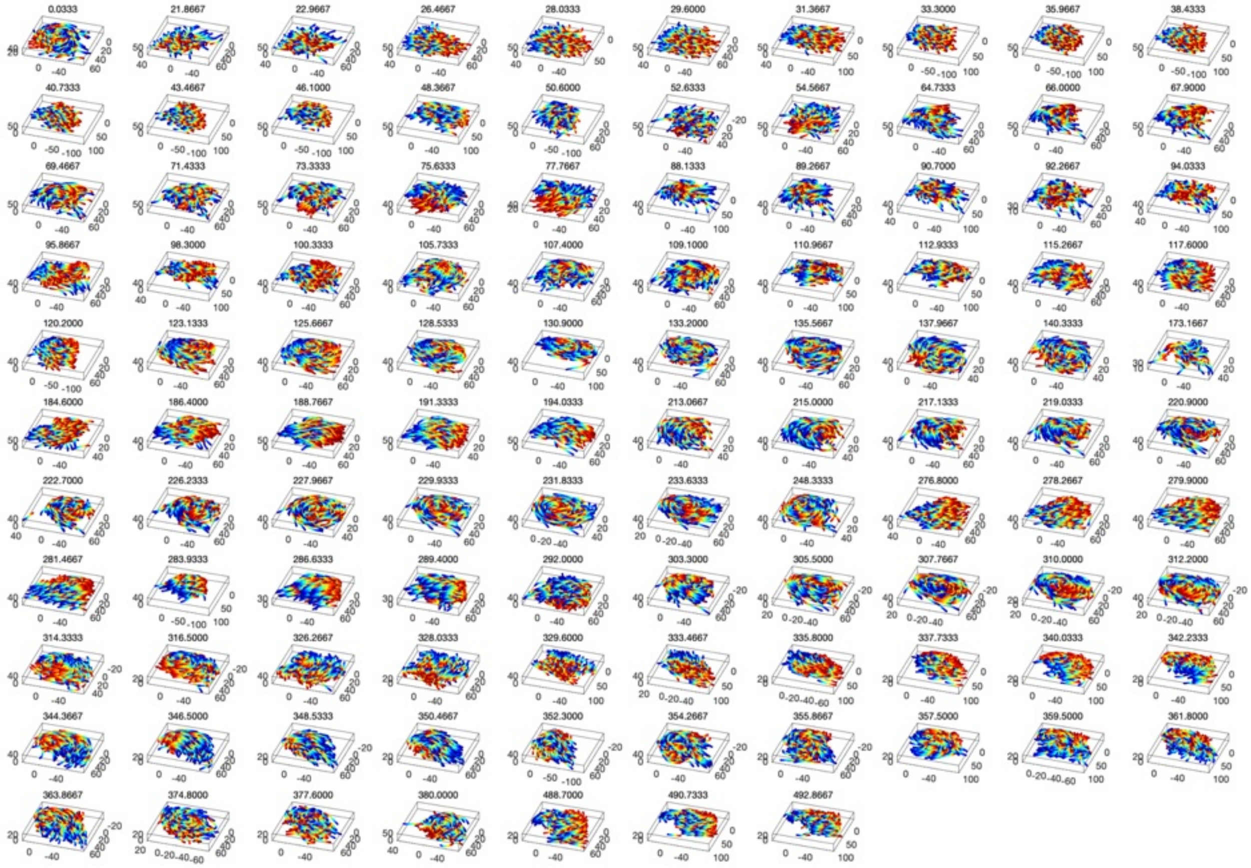

Supplementary Figure 8 | The short periods of continuous tracks of "circling B" flocks yielded by our workflow of data processing. Our workflow generates 107 short periods of continuous tracks from the original "circling B" dataset. Each 3D panel corresponds to a frame matrix. The top number in each panel indicates the frame time to generate the frame matrix. The gradient color from blue to red maps the flocking time from beginning to end.

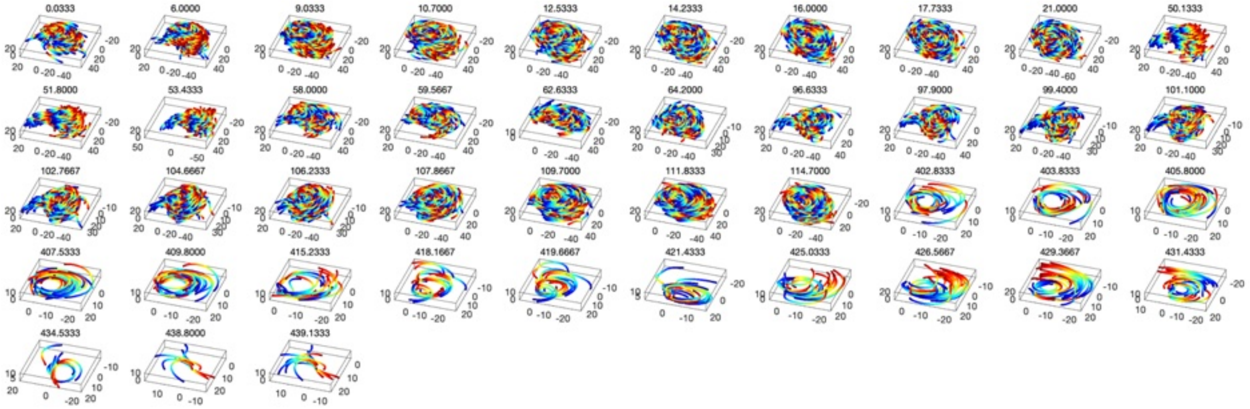

Supplementary Figure 9 | The short periods of continuous tracks of "circling C" flocks yielded by our workflow of data processing. Our workflow generates 43 short periods of continuous tracks from the original "circling C" dataset. Each 3D panel corresponds to a frame matrix. The top number in each panel indicates the frame time to generate the frame matrix. The gradient color from blue to red maps the flocking time from beginning to end.

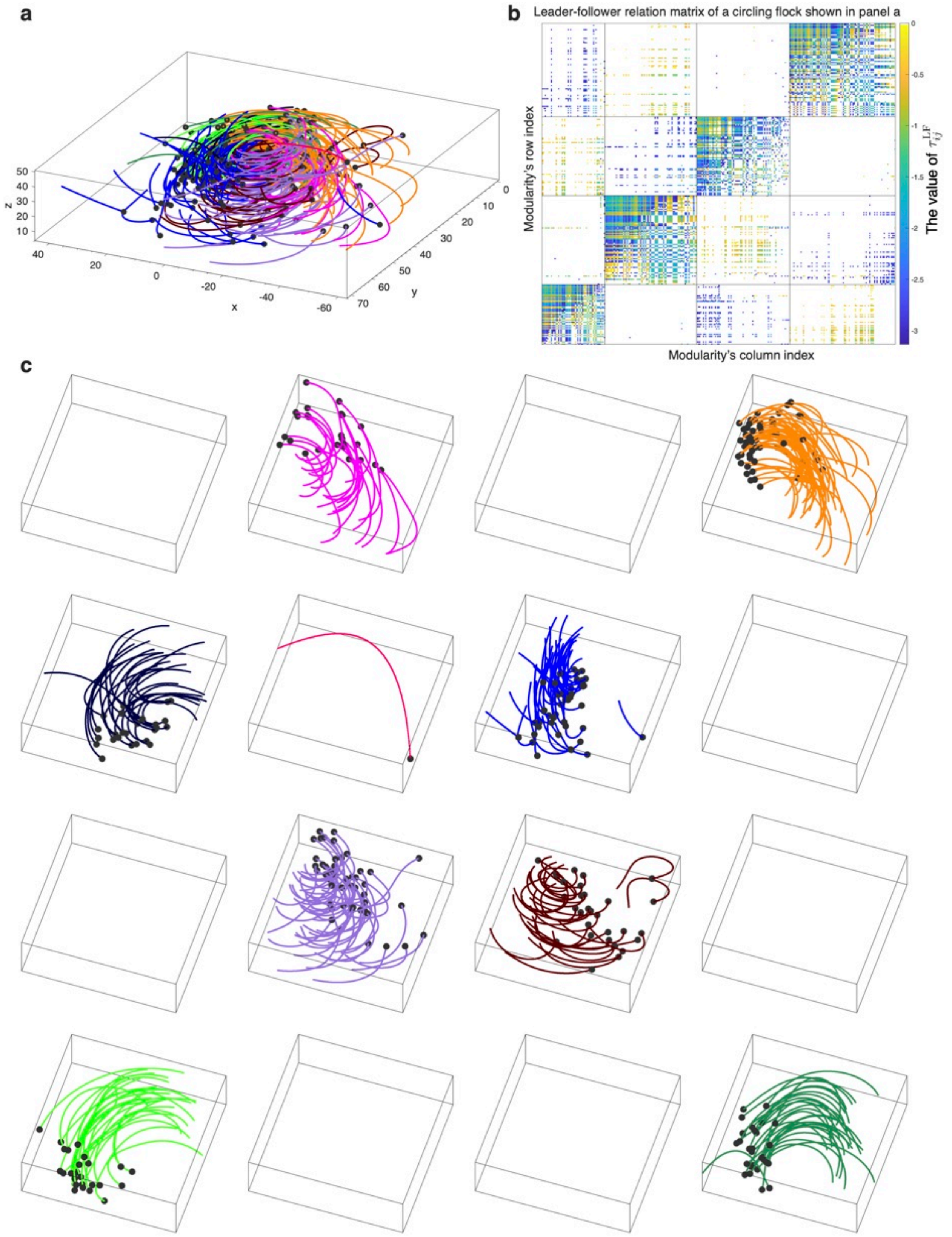

Supplementary Figure 10 | Using LF relation matrix to classify sub-communities in a circling flock. a, 3D trajectories of a circling flock. This flock contains 293 birds and is used in

Supplementary Movie 2 to show the sub-communities. **b,** The modularity of an LF relation matrix for the circling flock shown in panel a. After calculating the modularity by BiMat, the flock is divided into 16 modules. The gradient color from yellow to dark blue maps the value of τ_{ij}^{LF} and the white color represents $\tau_{ij}^{\mathrm{LF}} = 0$. Here the row and column are rearranged according to the modularity index of each individual. **c,** 3D trajectories of corresponding modules. The individuals belonging to a sub-community are the intersection set of row and column of each module in panel b. In panels a and c, the black dots represent the end of flock and different colors of trajectories correspond to the sub-communities.

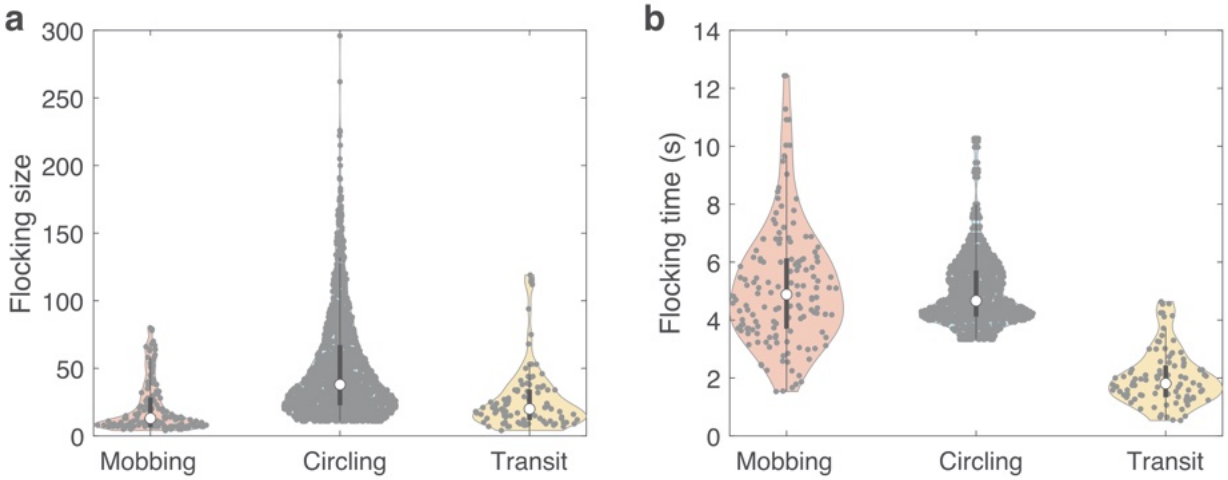

Supplementary Figure 11 | Comparison of flock size, recording time, average order and average angular momentum for mobbing, circling and transit datasets. Each point represents a flock. Note that the points of circling flocks in panel a,b correspond to all the non-empty sub-communities classified from 232 continuous tracks of circling flocks. Therefore, the number of mobbing, circling and transit flocks to be analyzed are 140, 1483 and 94, respectively. **a,** The flock size. **b,** The persisting time of each flock. Each grey dot represents a flock from three datasets, and the white points represent the median value.

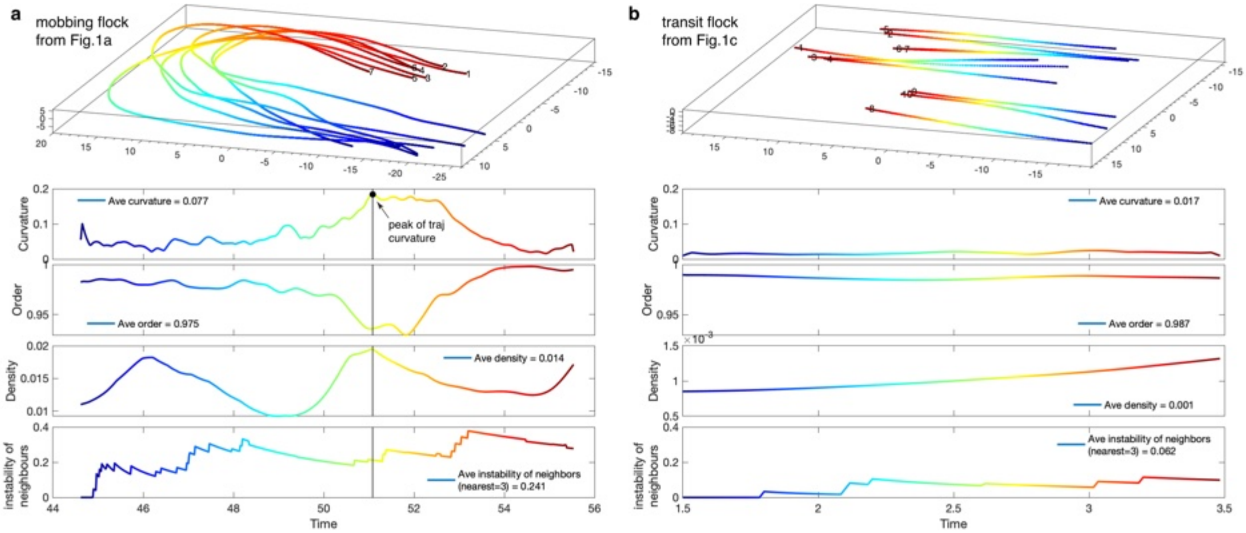

Supplementary Figure 12 | Examples of four flocking metrics for a mobbing or transit flock. Here four metrics, namely trajectory curvature, group order, group density and instability of neighbors over recording times, are evaluated within a mobbing (**a**) and transit (**b**) flock.

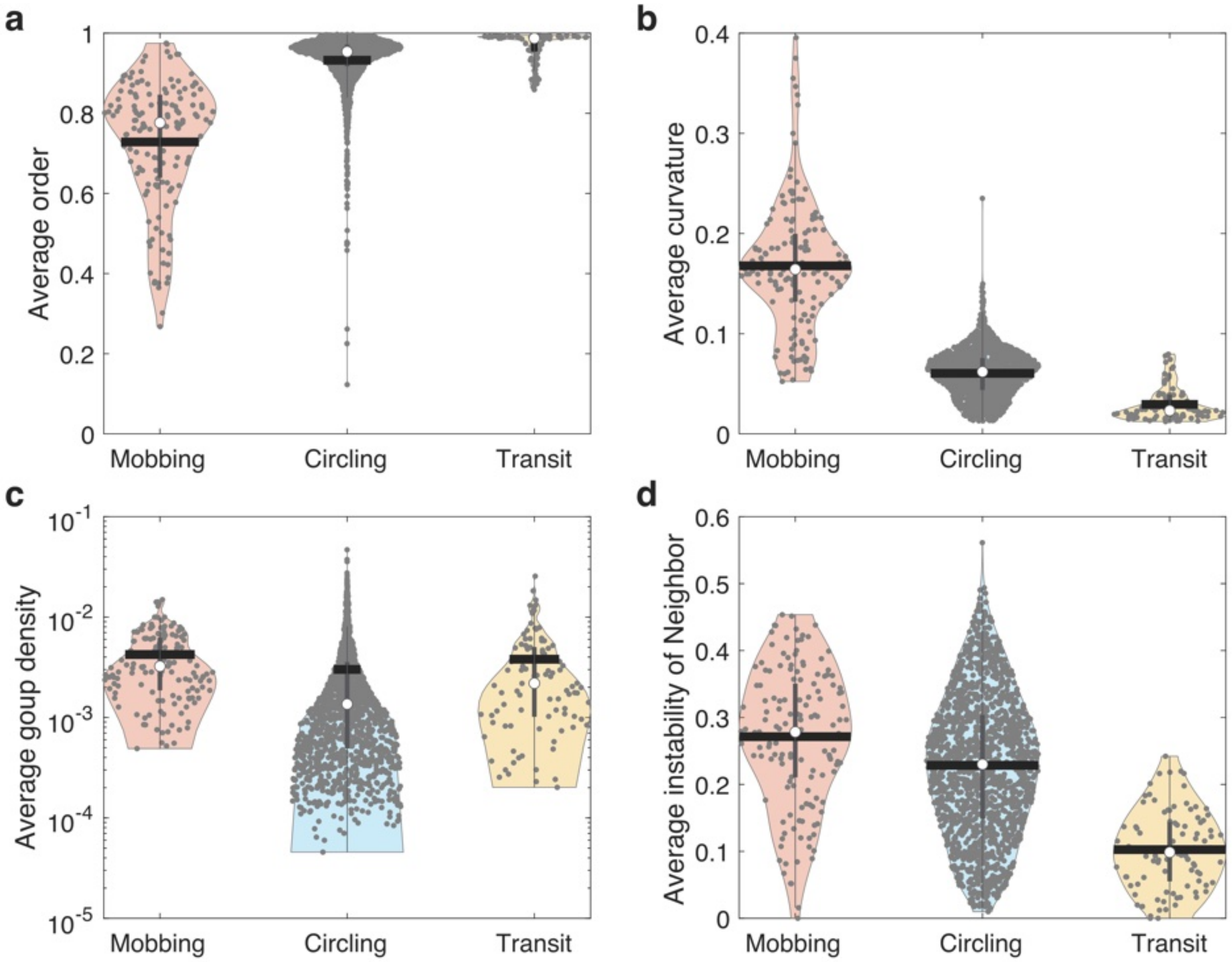

Supplementary Figure 13 | Comparison of average order, curvature, group density and instability of neighbors for mobbing, circling and transit datasets. Each point represents a flock. Note that the points of circling flocks in panel a-d correspond to all the non-empty sub-communities classified from 232 continuous tracks of circling flocks. Therefore, the number of mobbing, circling and transit flocks to be analyzed are 140, 1483 and 94, respectively. **a,** The average order over time. **b,** The average angular momentum over time. **c,** The average group density over time. **d,** The average instability of neighbor over time. Each grey dot represents a flock from three datasets, and the white points (or black lines) represent the median (or mean) value.

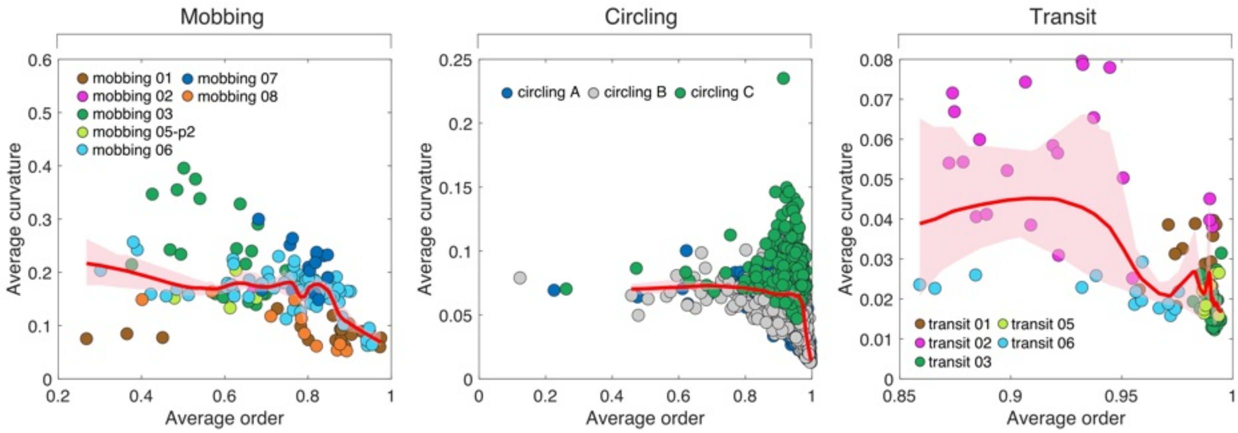

Supplementary Figure 14 | The relation between trajectory curvature and group order accords with our common sense and the observed collective motions across three datasets: the higher group order, the less trajectory curvature. Each point represents a flock from three datasets, and the nonparametric regression and bootstrap sampling are performed to calculate the trend (red curve) and its 94% confidence interval (red shadow) between two measurements.

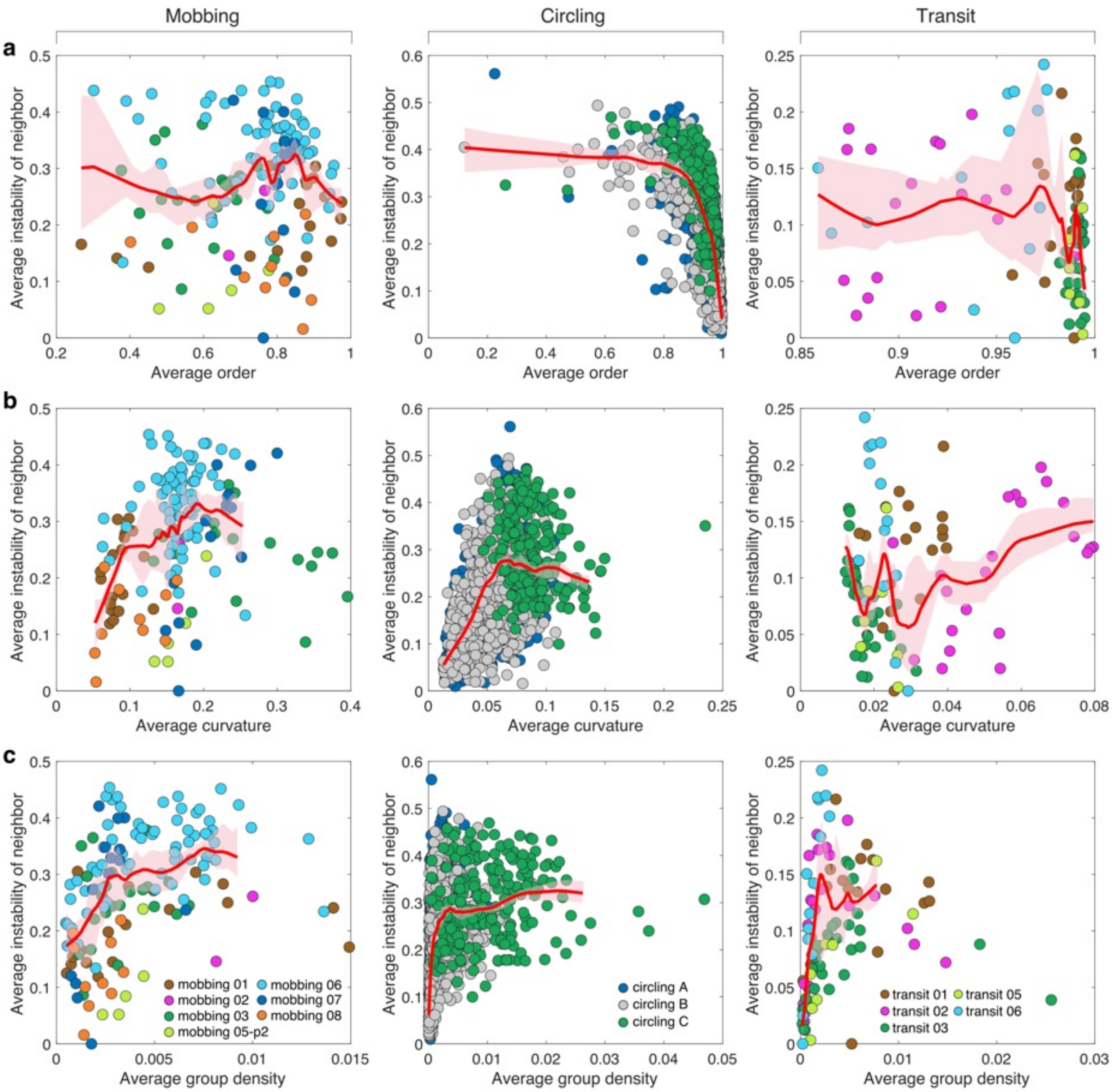

Supplementary Figure 15 | The relation between average instability of neighbors and other flocking metrics across three datasets. Average instability of neighbors vs average order (**a**), average curvature (**b**), and average group density (**c**), respectively**.** Each point represents a flock from three datasets, and the nonparametric regression and bootstrap sampling are performed to calculate the trend (red curve) and its 94% confidence interval (red shadow) between two measurements.

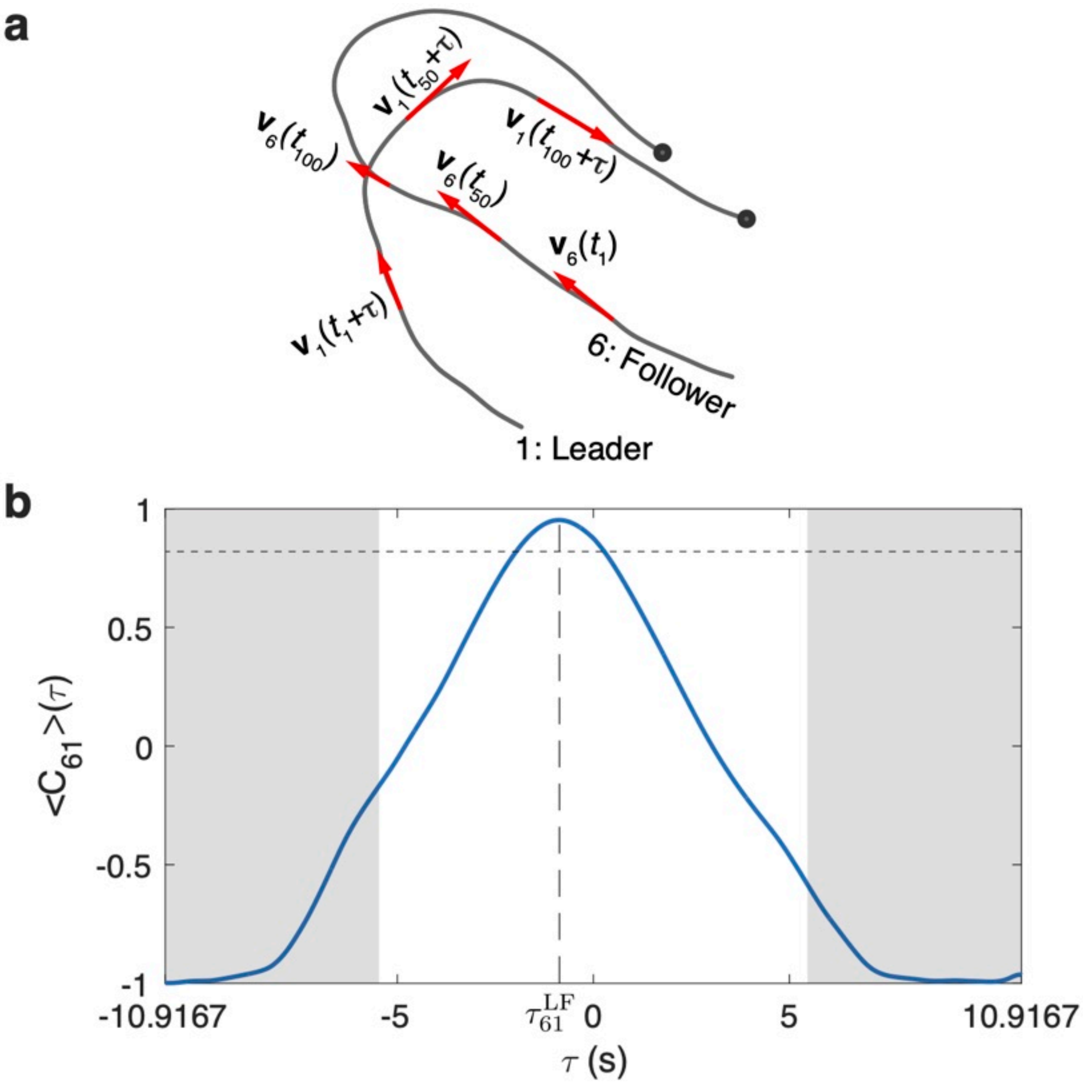

Supplementary Figure 16 | Diagram of determining the leader-follower relation according to the movement information of an individual pair. a, The trajectories of an individual pair from Fig.1a in the main text. For a given τ, we could get the velocity pairs with time evolving, such as, $(\mathbf{v}_6(t_1), \mathbf{v}_1(t_1 + \tau))$, $(\mathbf{v}_6(t_{50}), \mathbf{v}_1(t_{50} + \tau))$ and so on. As the frame rate (equals the time interval) of video recording is fixed for the flocks, t_i (i.e., t_1, t_{50} and t_{100}) represents the i-th time stamp of the flock. The velocity pairs with a fixed τ over the whole flock is first to calculate $\hat{\mathbf{v}}_6(t) \cdot \hat{\mathbf{v}}_1(t + \tau)$, and then to average them over different t as $\langle C_{61}\rangle(\tau)$. Note that the value of τ could be positive or negative. **b**, Collecting different τ, we could get the curve of $\langle C_{61}\rangle(\tau)$ as a function of τ. The maximal value of the curve of $\langle C_{61}\rangle(\tau)$ is labelled τ_{61}^{LF} to determine that individual-6,1 is leader or follower. As $\tau_{61}^{\mathrm{LF}} = -0.87 < 0$, bird-1 is the leader and bird-6 is the follower, because bird-6 falls 1.25 seconds behind to maintain the maximal alignment with bird-1. Note that if τ_{ij}^{LF} locates at first 25% or last 25% of τ-axis (light grey area), we disregard τ_{ij}^{LF} as we consider this τ to be too short for directional copying. Meanwhile we set the threshold of $\langle C_{ij}\rangle(\tau)$ as 0.8 to locate τ_{ij}^{LF} (beyond the horizontal dashed line).

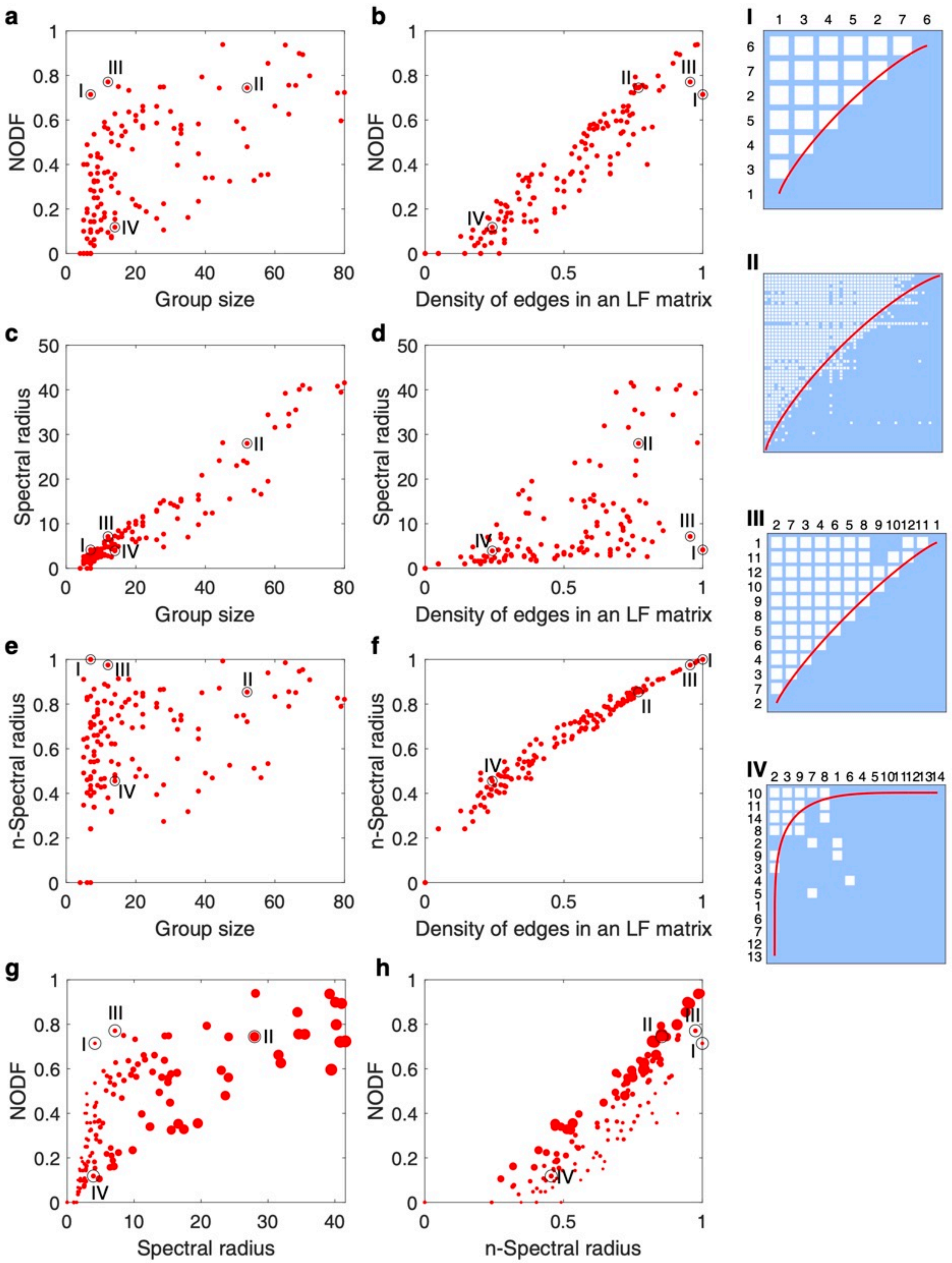

Supplementary Figure 17 | The comparison of NODF and Spectral radius of LF networks for Mobbing dataset. There are in total 140 mobbing flocks. Each point corresponds to a mobbing

flock. The NODF as a function of group size (**a**) and density of edges in an LF network (**b**). The Spectral Radius as a function of group size (**c**) and density of edges in an LF network (**d**). The normalized Spectral Radius as a function of group size (**e**) and density of edges in an LF network (**f**). The NODF as a function of corresponding Spectral Radius (**g**) and normalized Spectral Radius (**h**). The point size in panels g,h linearly scales with the corresponding group size. In the rightmost column (labeled by I,II,III,IV), we show four LF networks with nested-plot layout. We also label four LF networks in panels a-h.

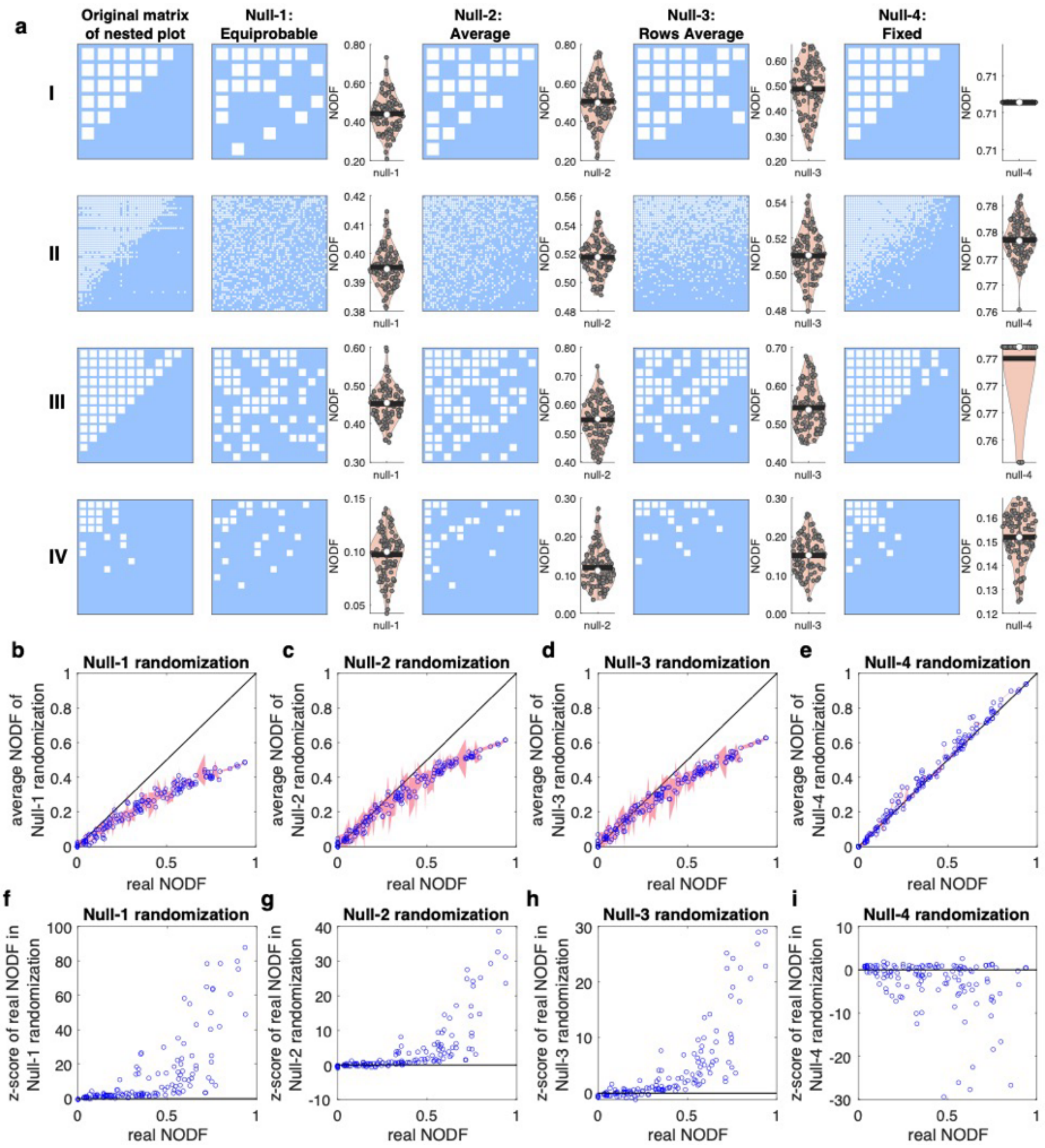

Supplementary Figure 18 | The null models of NODF. a, We present 4 null models for networks, each applied to the 4 LF networks of example-I, II, III, and IV. Within each row, the violin plot of NODF represents 100 independent randomizations. Each grey dot in Violin plot represents a flock from three datasets, and the white points (or black lines) represent the median (or mean) value. **b-e**, For the mobbing dataset, the average NODF of 4 null models based on 100 independent randomizations, displays as a function of the NODF of real LF networks. Each blue circle represents a mobbing flock, and the light red color represents the standard deviation of 100 independent randomizations. **f-i**, For the mobbing dataset, the z-score of real NODF value within the distribution of random NODF values generated from 4 null models respectively. Each point

represents a mobbing flock, the x-axis is real NODF value calculated from the LF matrix of this flock, and the y-axis is z-score of this real NODF value within 100 random NODF values.

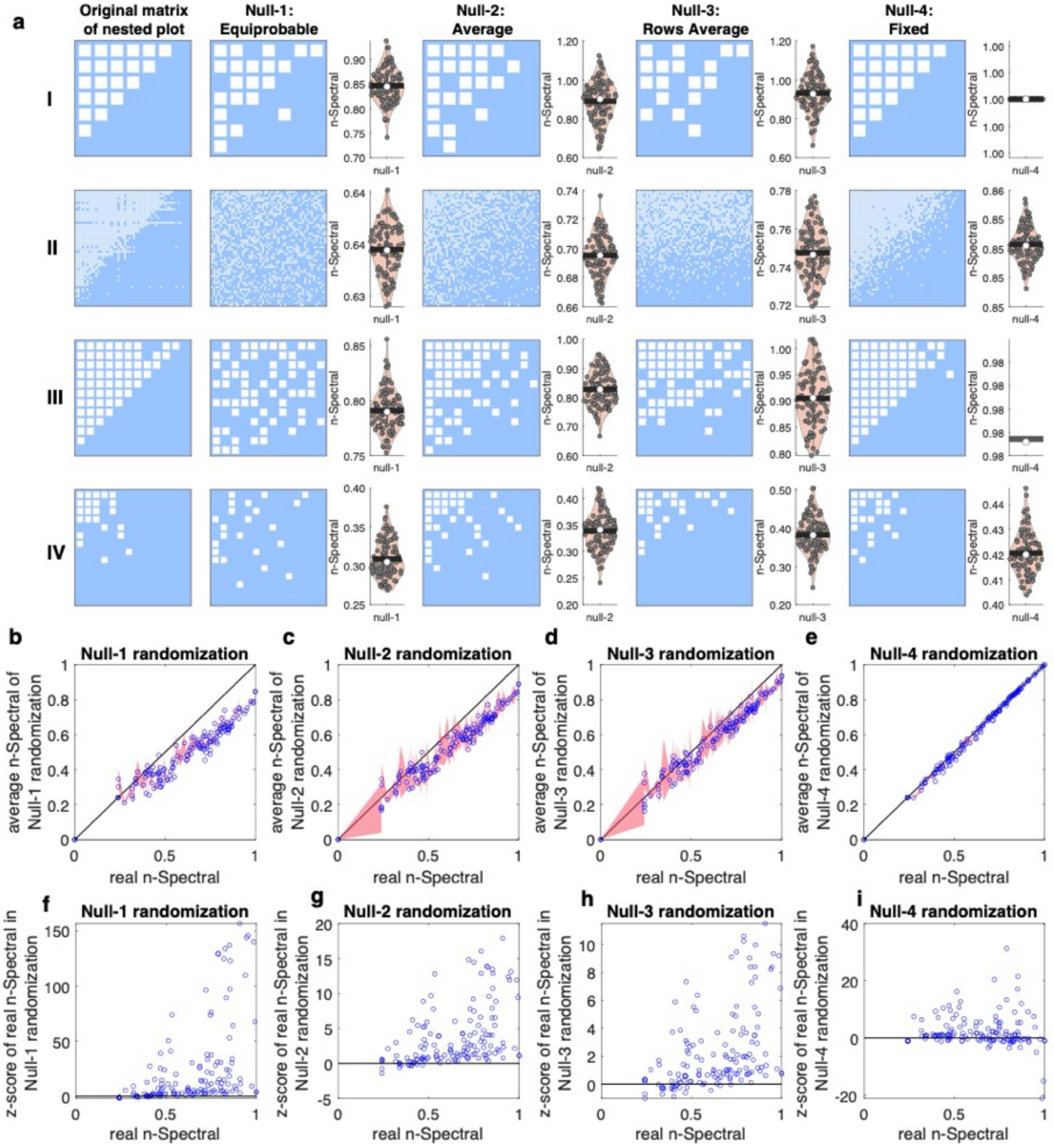

Supplementary Figure 19 | The null models of normalized Spectral Radius. a, We present 4 null models for networks, each applied to the 4 LF networks of example-I, II, III, and IV. Within each row, the violin plot of normalized Spectral Radius represents 100 independent randomizations. Each grey dot in Violin plot represents a flock from three datasets, and the white points (or black lines) represent the median (or mean) value. **b-e**, For the mobbing dataset, the average n-Spectral Radius of 4 null models based on 100 independent randomizations, displays as a function of the n-Spectral Radius of real LF networks. Each blue circle represents a mobbing flock, and the light red color represents the standard deviation of 100 independent randomizations. **f-i**, For the mobbing dataset, the z-score of real n-Spectral Radius value within the distribution of random n-

Spectral Radius values generated from 4 null models respectively. Each point represents a mobbing flock, the x-axis is real n-Spectral Radius value calculated from the LF matrix of this flock, and the y-axis is z-score of this real n-Spectral Radius value within 100 random n-Spectral Radius values.

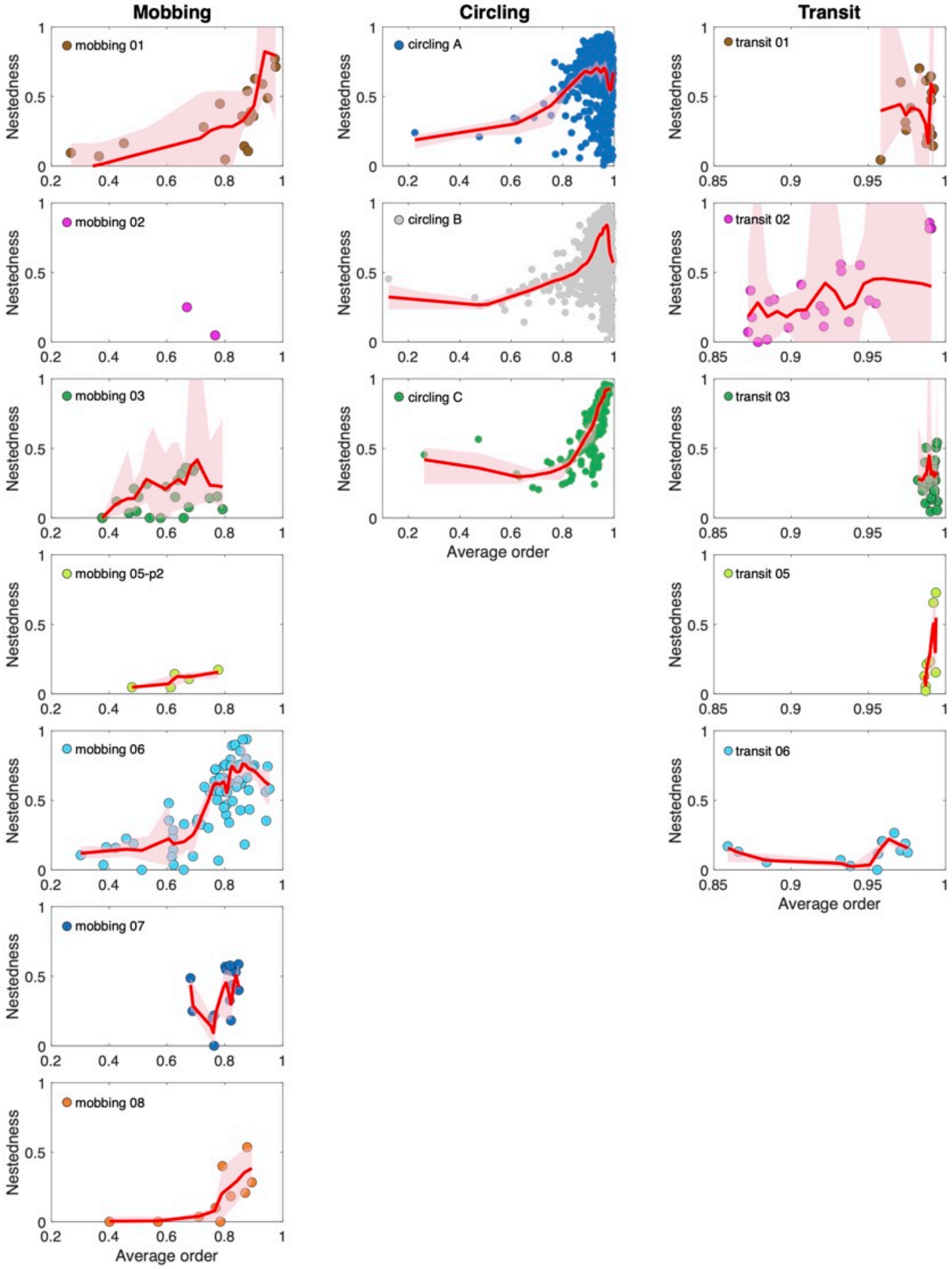

Supplementary Figure 20 | The scatter plot between nestedness of LF networks and average order for different flock id. Different with Fig.1g-i that we collect flocks with different id

together to show the relations between nestedness of LF networks and group order, we separately calculate the relations of each flock id for three flocking datasets. Each point represents a flock, and the nonparametric regression and bootstrap sampling are performed to calculate the trend (red curve) and its 94% confidence interval (red shadow) between nestedness and average order.

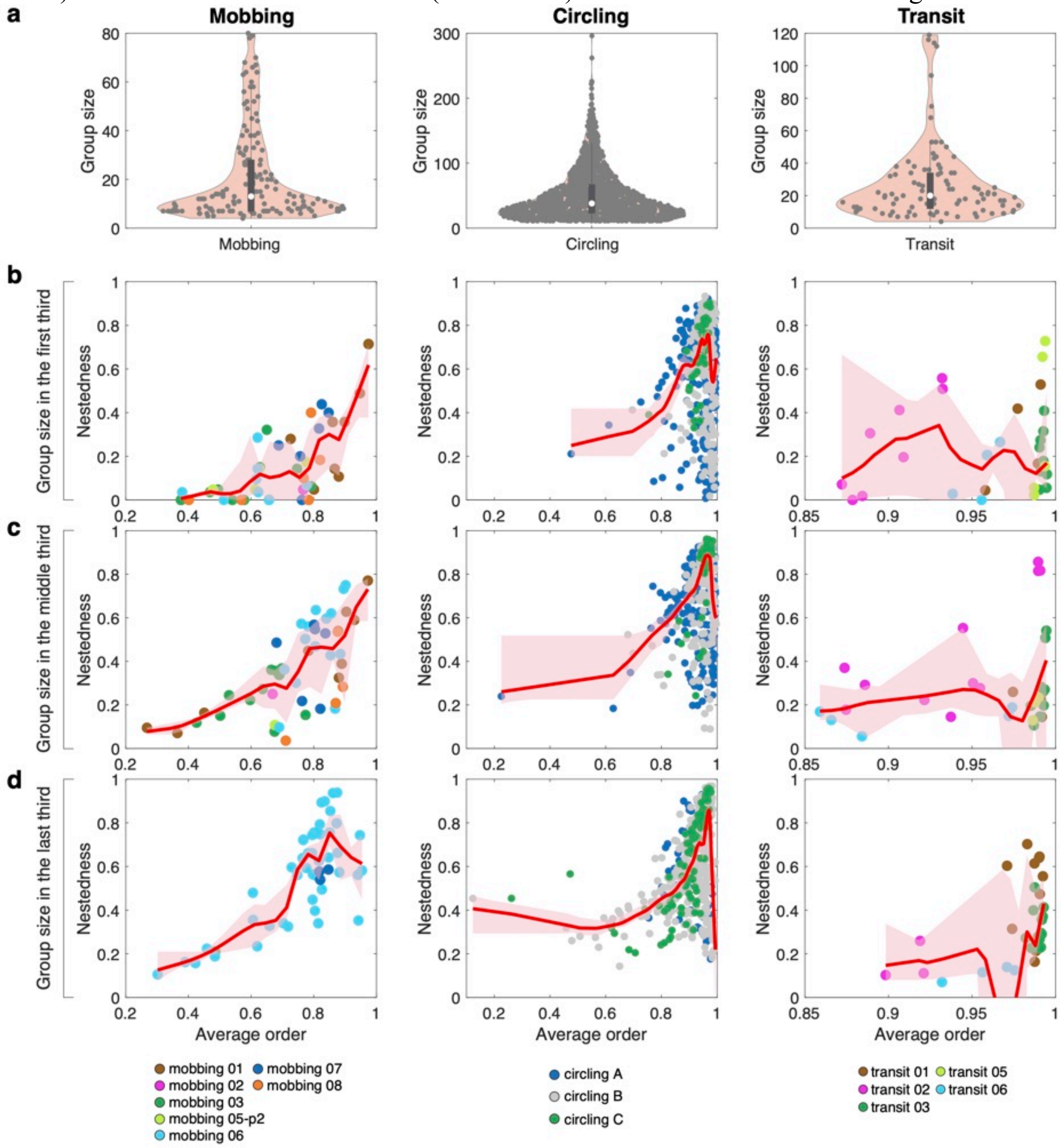

Supplementary Figure 21 | The scatter plot between nestedness of LF networks and average order for different proportions of group size. a, The distribution of group size for three datasets. Each grey dot represents a flock from three datasets, and the white points represent the median value. According to group size, we divide the flocks into three parts and then to calculate their nestedness of LF networks and average order: (**b**) the first third of all group size, (**c**) the middle third of all group size and (**d**) the last third of all group size. Each point represents a flock from

three datasets, and the nonparametric regression and bootstrap sampling are performed to calculate the trend (red curve) and its 94% confidence interval (red shadow) between nestedness and average order.

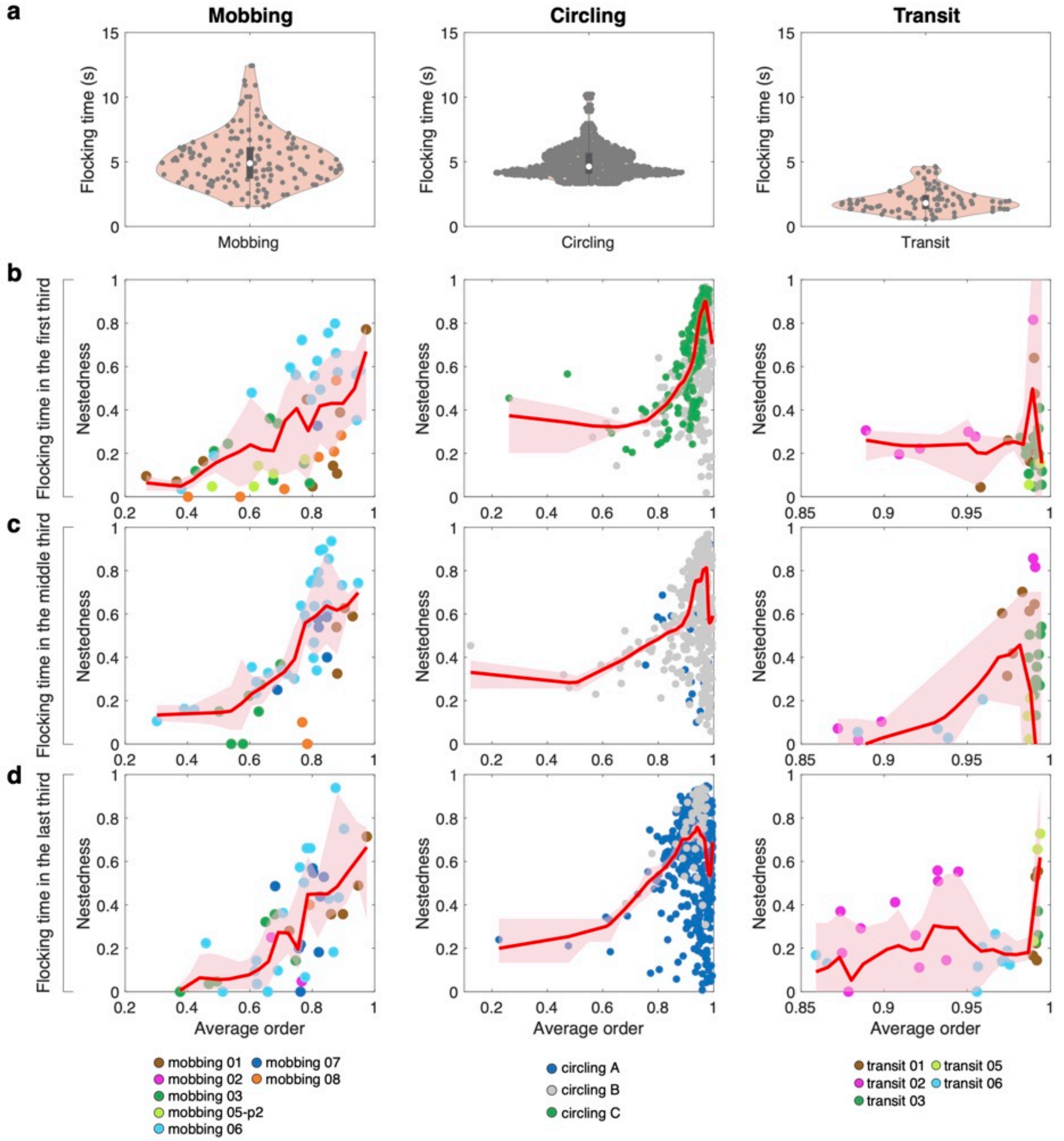

Supplementary Figure 22 | The scatter plot between nestedness of LF networks and average order for different proportions of flocking time. a, The distribution of flocking time (or called duration) for three datasets. Each grey dot in represents a flock from three datasets, and the white points represent the median value. According to flocking time, we divide the flocks into three parts and then to calculate their nestedness of LF networks and average order: (**b**) the first third of all flocking time, (**c**) the middle third of all flocking time and (**d**) the last third of all flocking time.

Each point represents a flock from three datasets, and the nonparametric regression and bootstrap sampling are performed to calculate the trend (red curve) and its 94% confidence interval (red shadow) between nestedness and average order.

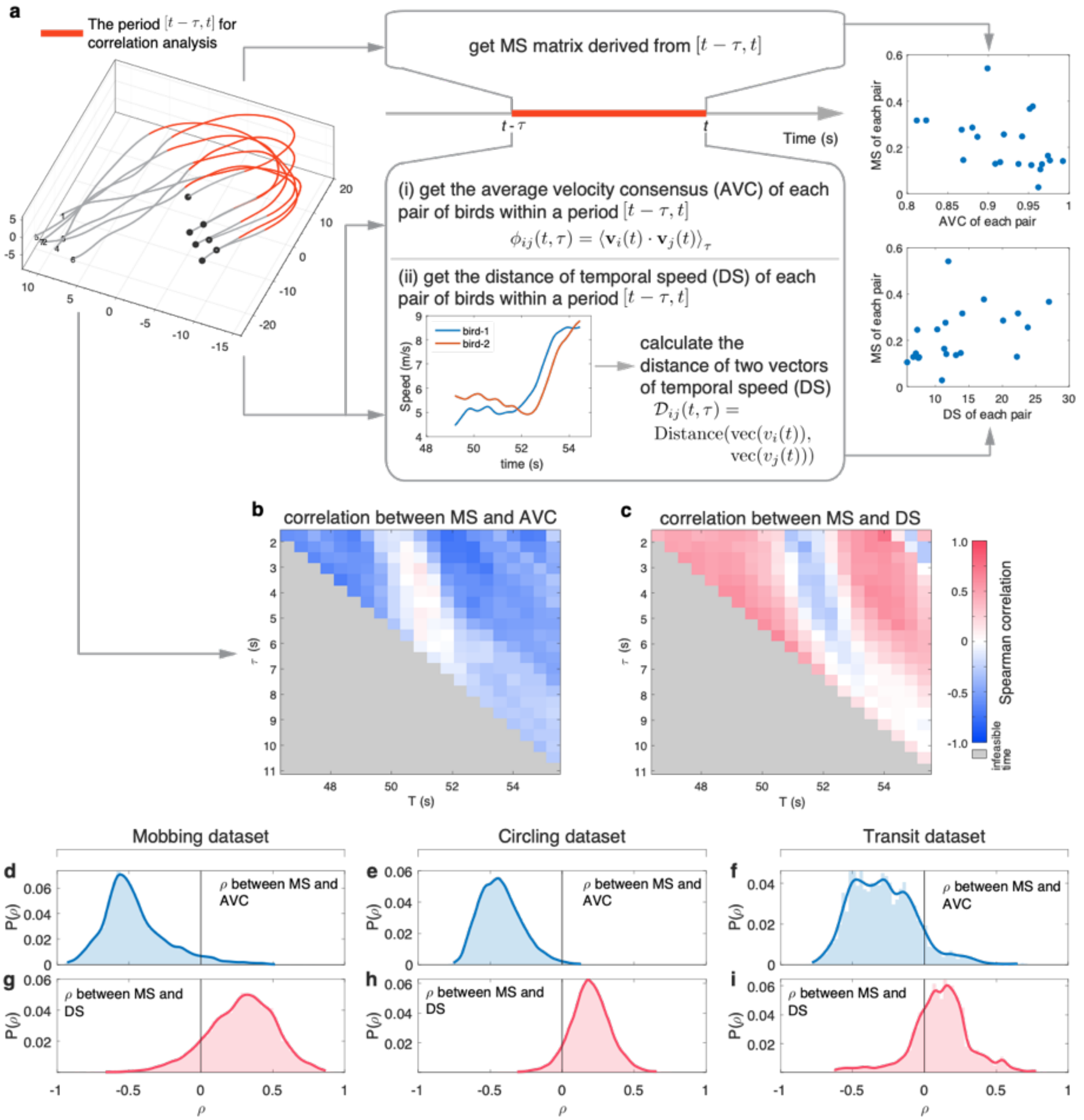

Supplementary Figure 23 | The correlation analysis between MS and other two metrics quantifying the motion differences by velocity and speed. a, For a period $[t-\tau, t]$ highlighted by red, we perform the correlation analysis for all pairs of birds between MS and other two metrics: (i) average velocity consensus (AVC) of each pair of birds, denoted as $\phi_{ij}(t,\tau) = \langle \mathbf{v}_i(t) \cdot \mathbf{v}_j(t) \rangle_\tau$; (ii) distance of temporal speed (DS) of each pair of birds, denoted as $\mathcal{D}_{ij}(t,\tau) = \mathrm{Distance}\left(\mathrm{vec}\left(v_i(t)\right), \mathrm{vec}\left(v_j(t)\right)\right)$. For simplicity, the DS value $\mathcal{D}_{ij}(t,\tau)$ is calculated by the Euclidean distance of two vectors composed of v_i and v_j within the period $[t-\tau, t]$. From the

flock shown in panel a, the heatmap of Spearman correlation of MS-AVC (**b**) and MS-DS (**c**) under various combinations of t and τ. The distribution of Spearman correlation of MS-AVC (**d-f**) and MS-DS (**g-i**) from three bird flocking datasets. In panels b-i, for each flock, we take $\alpha = 0$ to calculate MS, 21 time stamps for t, and 22 time points for τ.

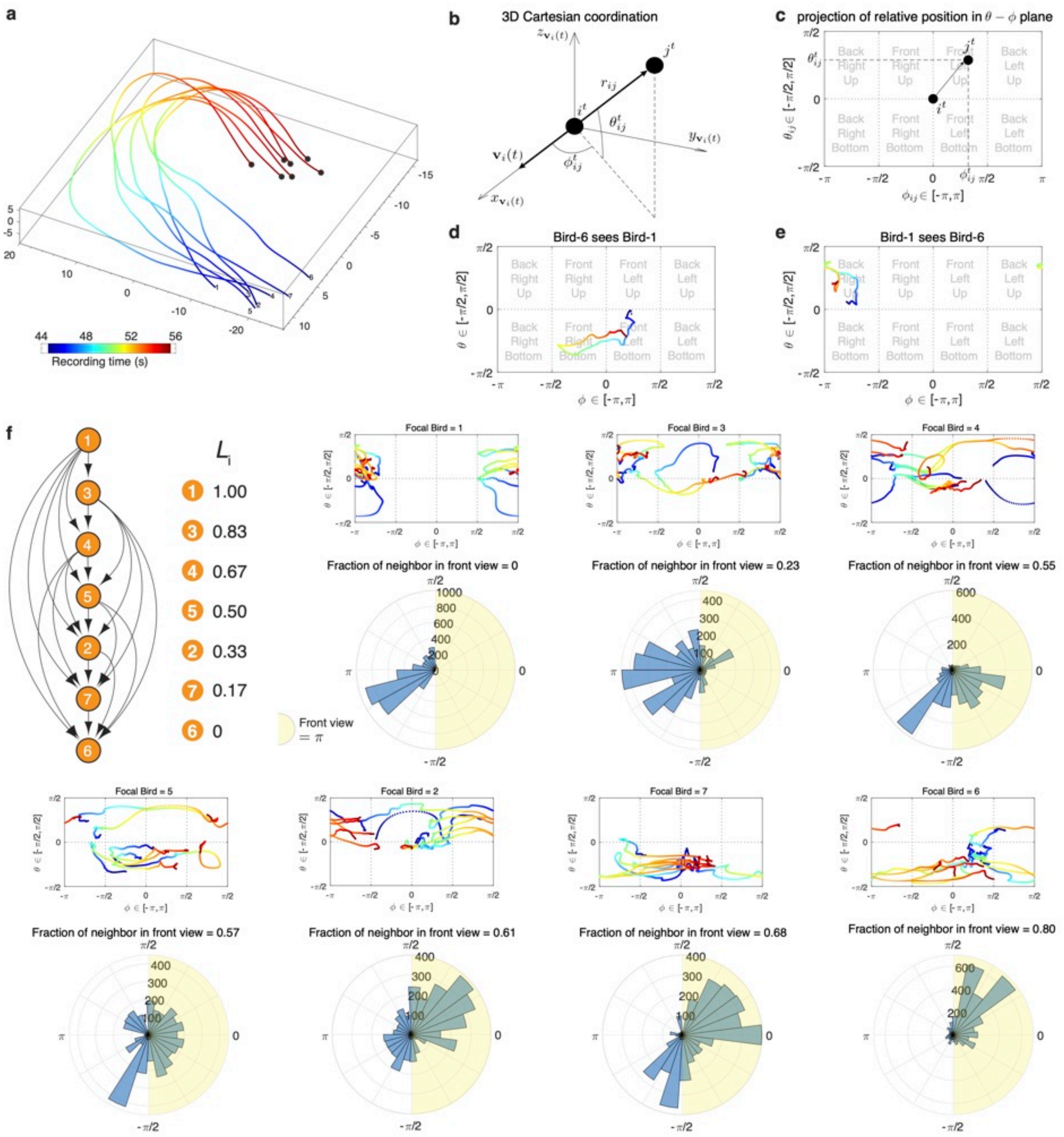

Supplementary Figure 24 | Spatial structure between leading tier and fraction of neighbors present in the front view. a, The flock is the same with Fig.1a in the main text. **b**, The relative 3D Cartesian coordination from the view of the focal individual-i at time t. The origin is the center of individual-i and the direction of x-axis follows $\mathbf{v}_i(t)$. $(r_{ij}^t, \theta_{ij}^t, \phi_{ij}^t)$ in the relative 3D Cartesian coordination represents the relative position between individual-i and neighbor-j. r_{ij}^t is distance, $\theta_{ij}^t \in [-\pi/2, \pi/2]$ is the zenith angle, and $\phi_{ij}^t \in [-\pi, \pi]$ is the azimuth angle. **c**, Projection of relative position of neighbor-j on $\theta - \phi$ plane according to $(r_{ij}^t, \theta_{ij}^t, \phi_{ij}^t)$. The $\theta - \phi$ plane could be divided into 8 parts from the view of the focal individual-i. With the time evolving, it makes up the continuous trajectory of relative position projecting to $\theta - \phi$ plane, such as "Bird-6 sees

Bird-1" (**d**) and "Bird-1 sees Bird-6" (**e**). **f**, The LF network and leading tier of each individual from the flock shown in panel a. For the range of front view $= \pi$ (highlighted by light yellow), we show the projection of relative positions in $\theta - \phi$ plane and the histogram of neighbors present in the view for all individuals over the whole time of flocks. In panels d-f, the gradient color (from blue to red, same with that of panel a) of curves in $\theta - \phi$ plane corresponds to the flocking time from beginning to end.

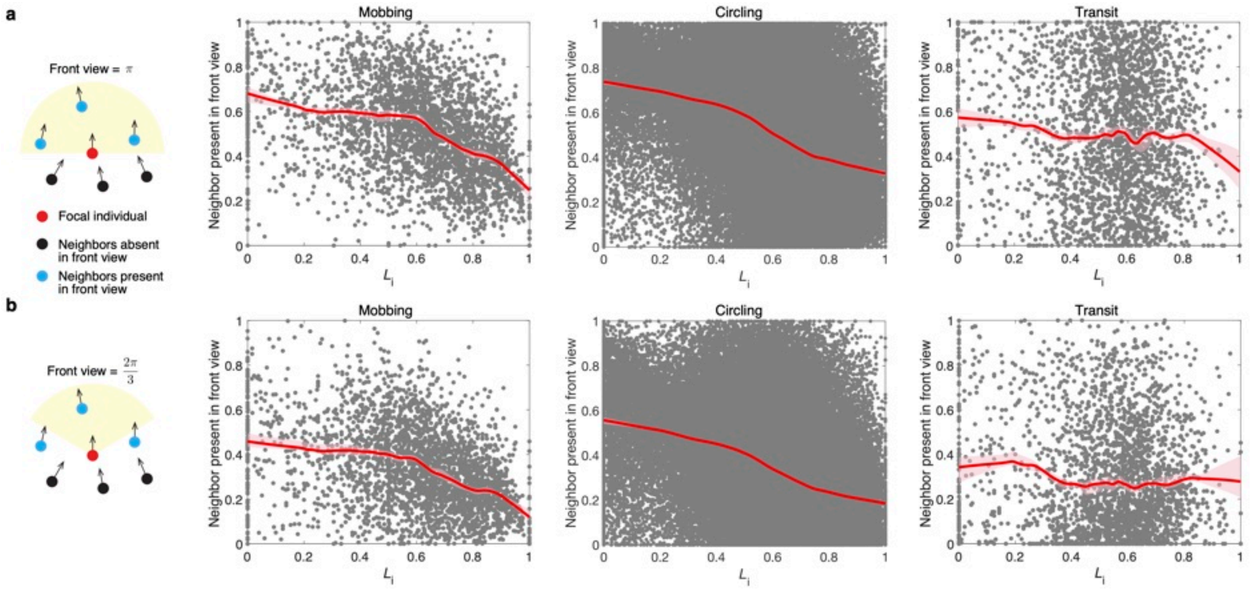

Supplementary Figure 25 | Correlation between leading tier and fraction of neighbors present in the front view for each individual. Scatter plot of leading tier ($L_i \in [0,1]$) and fraction of neighbors present in a given front view ($\in [0,1]$) for three flocking datasets when we set the range of front view is π (**a**) or $2\pi/3$ (**b**). L_i is calcuated from an LF network derived by the whole flock, and the fraction of neighbors present in front view accounts for the birds accumulatively present in the front view during the whole flocking time. Note that in this figure each point represents an individual from a flock rather than a flock. For example, in Supplementary Figure 24f, the flock contains 7 elements of L_i and fraction of neighbors in front view, respectively. The nonparametric regression and bootstrap sampling are performed to calculate the trend (red curve) and its 94% confidence interval (red shadow) between leading tier and fraction of neighbor present in the front view.

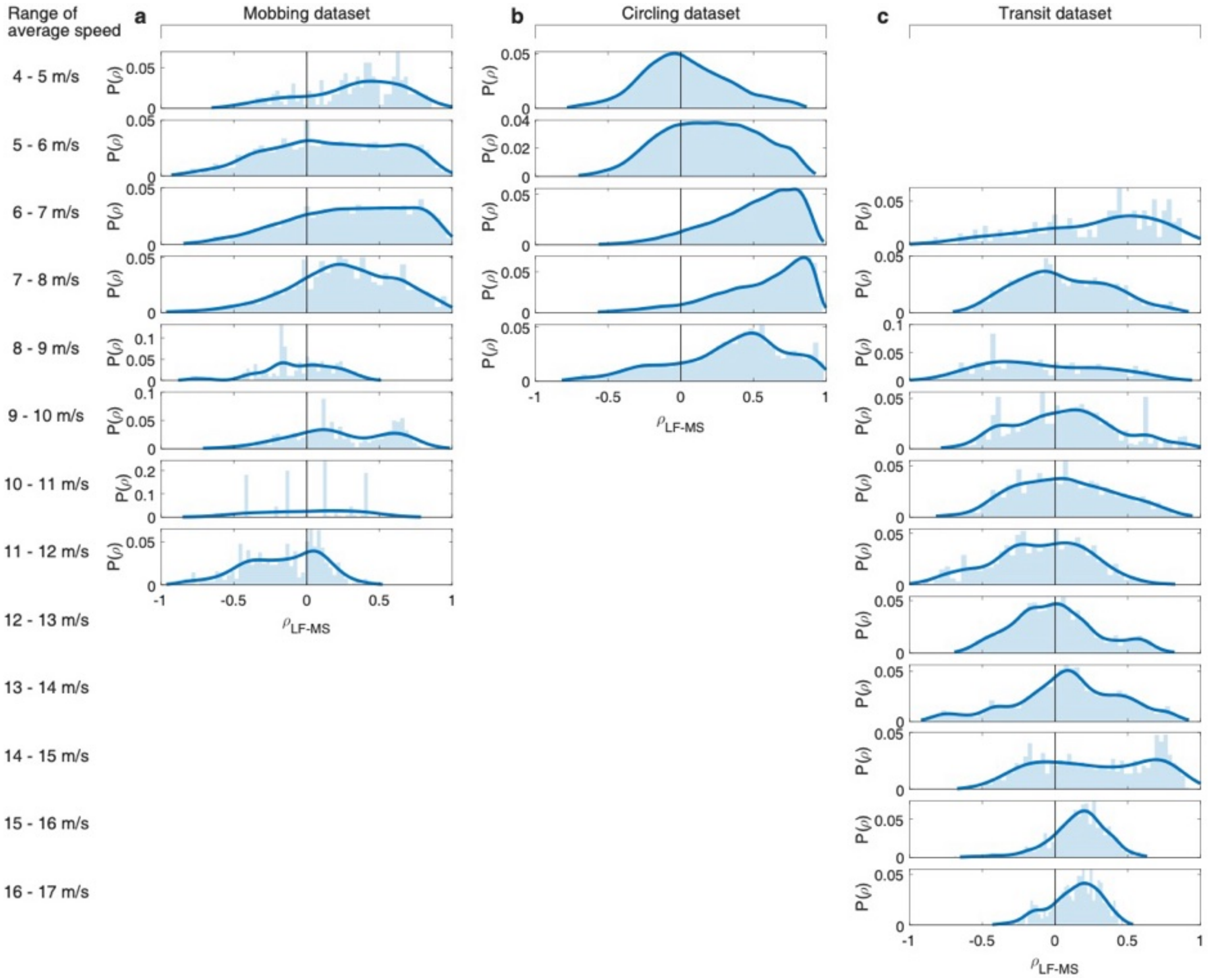

Supplementary Figure 26 | The distribution of correlation between LF and MS if the flocking dataset is categorized by average speed in 1m/s interval. Here the speed range is categorized by the flock's average speed. Then the flocks from mobbing (**a**), circling (**b**) and transit (**c**) datasets are performed by the correlation analysis between LF and MS as shown in Fig.3a.

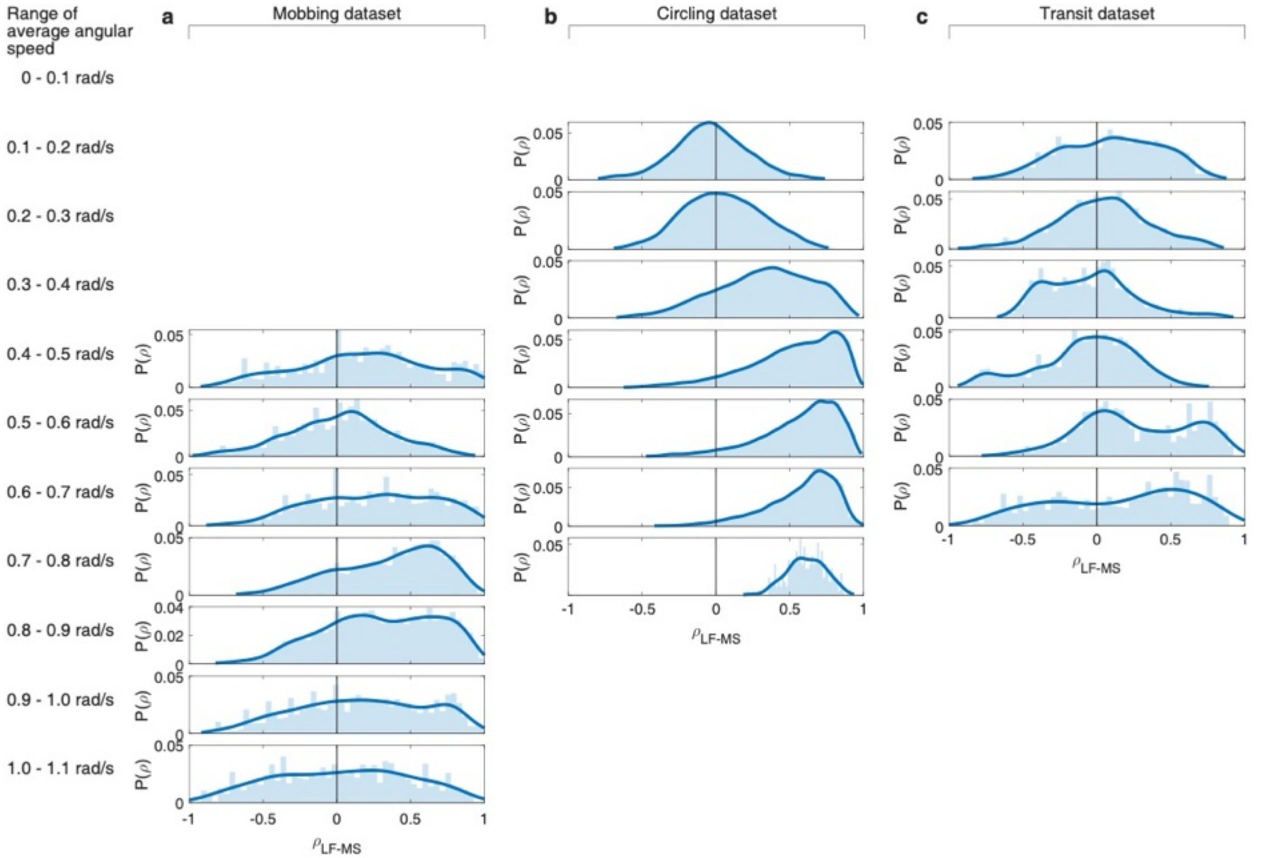

Supplementary Figure 27 | The distribution of correlation between LF and MS if the flocking dataset is categorized by average angular speed in 0.1rad/s interval. Here the range of average angular speed is categorized by the flock's average angular speed. Then the flocks from mobbing (**a**), circling (**b**) and transit (**c**) datasets are performed by the correlation analysis between LF and MS as shown in Fig.3a.

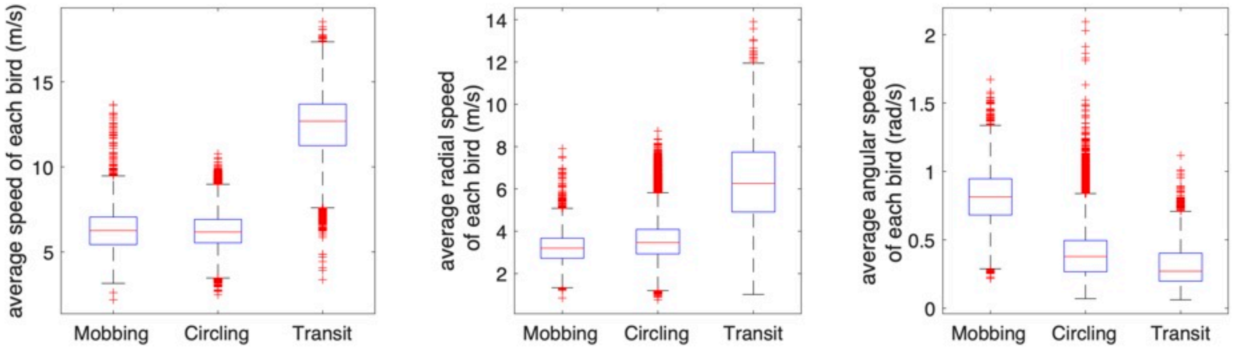

Supplementary Figure 28 | Statistics of bird's average speed, radial and angular speed for three flocking datasets. For mobbing, circling and transit datasets, we count three speeds of 3103,

75683, and 2597 birds, respectively. Note that each bird's average speed, radial and angular speed is calculated over the entire duration of a flock.

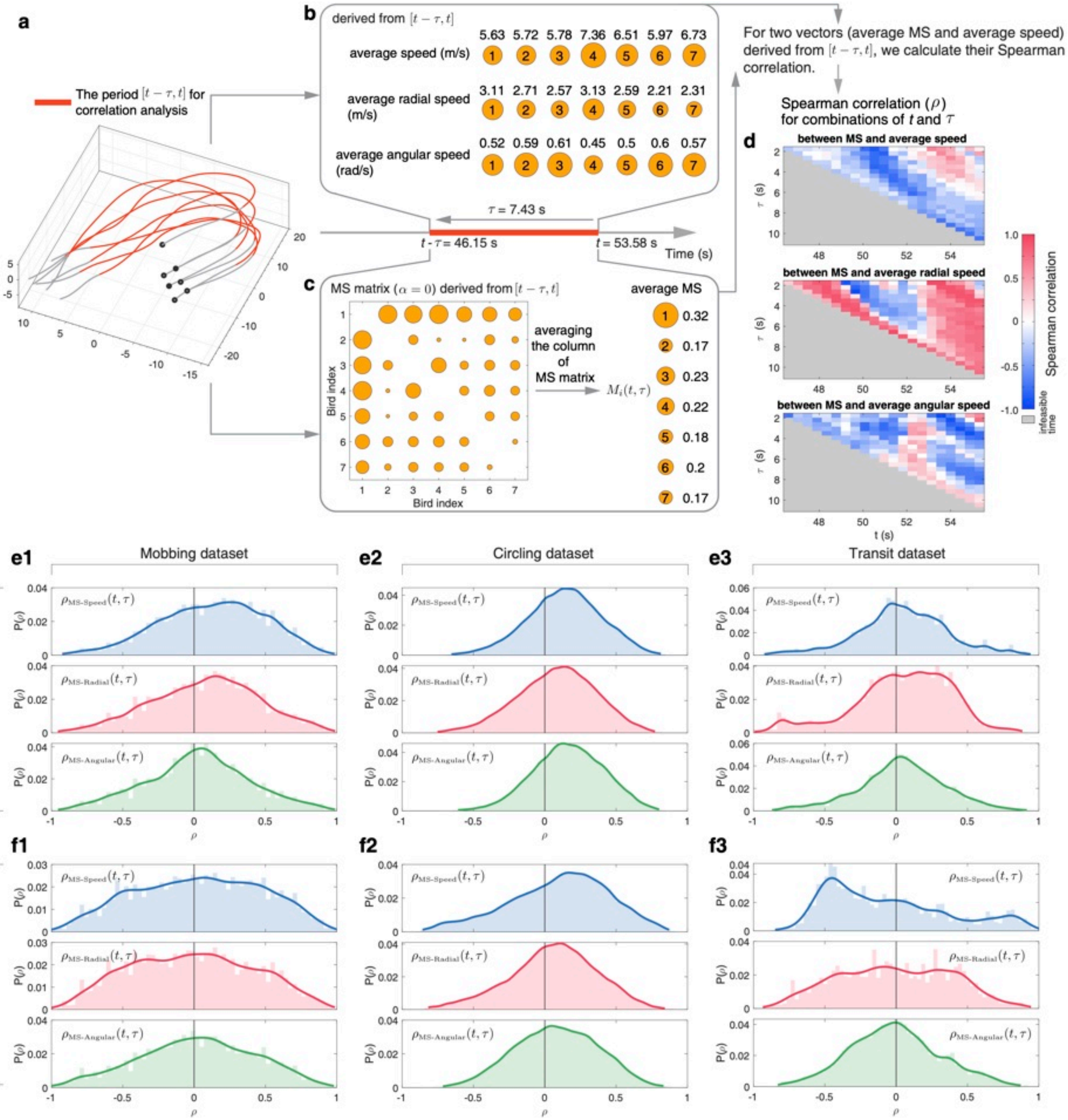

Supplementary Figure 29 | The workflow to perform the correlation analysis between MS and three kinds of speed. Similar with Fig.3a in the main text, a period of flock $[t-\tau,t]$ (a) could derive each bird's average speed, radial and angular speed (b), and average MS of each individual (c). For two vectors, such as average MS and average speed derived from $[t-\tau,t]$, we calculate their Spearman correlation (ρ). Finally, we could get the ρ between MS and three kinds of speed for different combinations of (t,τ) within a flock (d). We extend the correlation analysis to three datasets and yielded the distribution of $\rho_{\text{MS-Speed}}(t,\tau)$, $\rho_{\text{MS-Radial}}(t,\tau)$ and

$\rho_{\text{MS-Angular}}(t, \tau)$. Here we take MS with $\alpha = 0$ (e) and $\alpha = 1$ (f). For each flock, we take 21 time stamps for t and 22 time points for τ no matter how long a flock lasts.

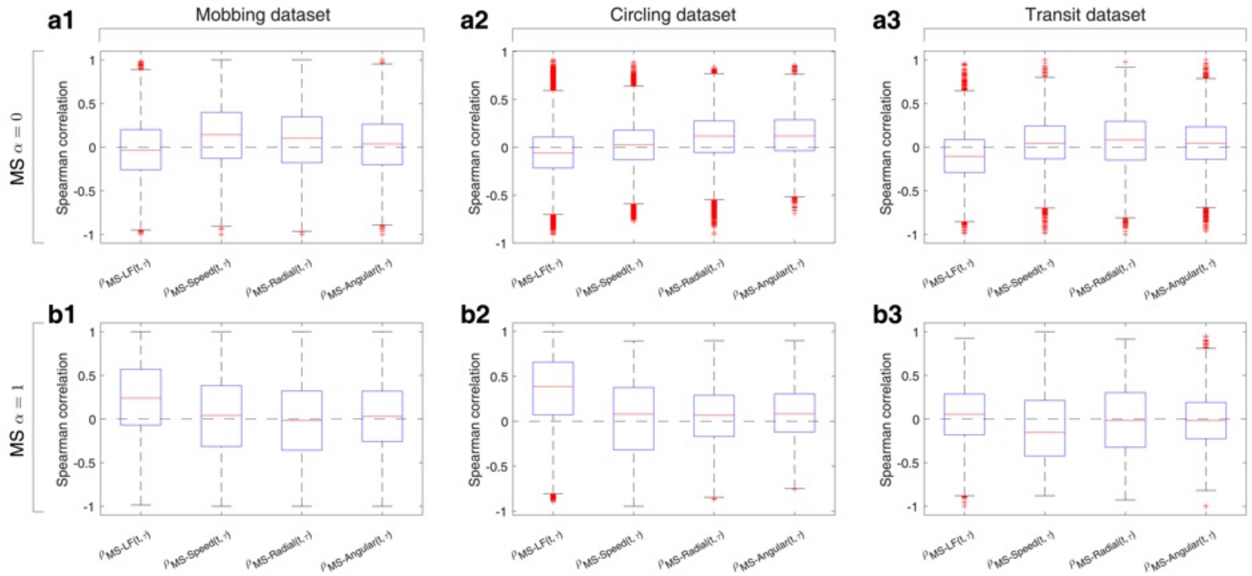

Supplementary Figure 30 | Boxplot of the correlation of MS-LF and three types of MS-Speed for three flocking datasets. In order to facilitate a clear comparison of the correlation levels between MS-LF (as shown in Fig.3 of the main text) and three types of MS-Speed, we present the boxplots of $\rho_{\text{MS-LF}}(t, \tau)$, $\rho_{\text{MS-Speed}}(t, \tau)$, $\rho_{\text{MS-Radial}}(t, \tau)$ and $\rho_{\text{MS-Angular}}(t, \tau)$ for mobbing, circling and transit datasets.

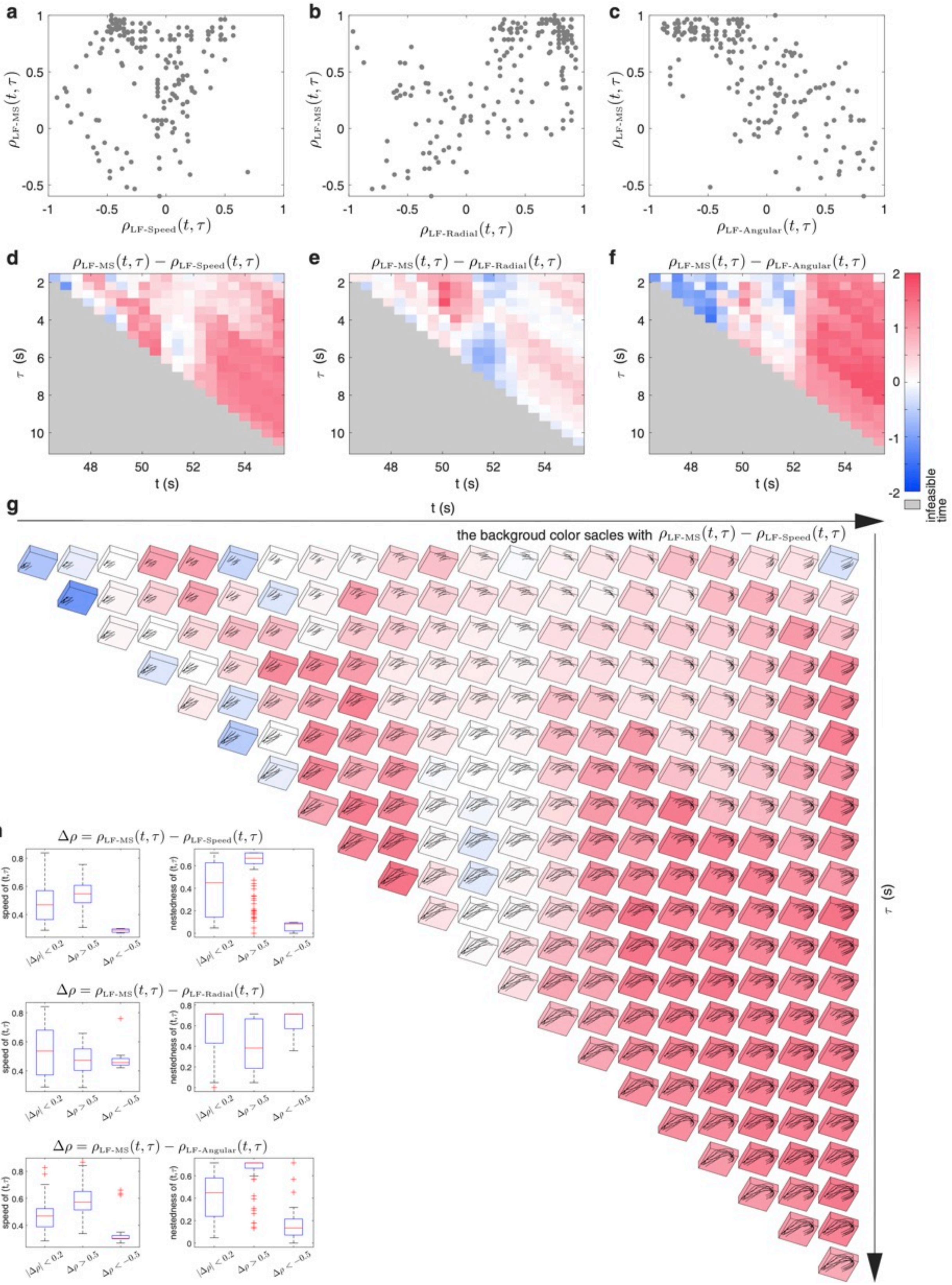

a
b
c
d
e
f
g
h
i
j
$\rho_{\text{LF-MS}}(t,\tau)$
$\rho_{\text{LF-Speed}}(t,\tau)$
$\rho_{\text{LF-Radial}}(t,\tau)$
$\rho_{\text{LF-Angular}}(t,\tau)$
$\rho_{\text{LF-MS}}(t,\tau) - \rho_{\text{LF-Speed}}(t,\tau)$
$\rho_{\text{LF-MS}}(t,\tau) - \rho_{\text{LF-Radial}}(t,\tau)$
$\rho_{\text{LF-MS}}(t,\tau) - \rho_{\text{LF-Angular}}(t,\tau)$
τ (s)
t (s)
infeasible time
the backgroud color sacles with $\rho_{\text{LF-MS}}(t,\tau) - \rho_{\text{LF-Speed}}(t,\tau)$
$\Delta\rho = \rho_{\text{LF-MS}}(t,\tau) - \rho_{\text{LF-Speed}}(t,\tau)$
$\Delta\rho = \rho_{\text{LF-MS}}(t,\tau) - \rho_{\text{LF-Radial}}(t,\tau)$
$\Delta\rho = \rho_{\text{LF-MS}}(t,\tau) - \rho_{\text{LF-Angular}}(t,\tau)$
average angular speed of (t,τ)
nestedness of (t,τ)
$|\Delta\rho| < 0.2$
$\Delta\rho > 0.5$
$\Delta\rho < -0.5$

Supplementary Figure 31 | Illustration of comparison of correlation of LF-MS and LF-Speed for a mobbing flock. Here we use a mobbing flock (shown in Fig.1a of the main text) to show the workflow of comparison. **a-c**, we perform the Spearman correlation to yield $\rho_{\text{LF-MS}}(t,\tau)$ and $\rho_{\text{LF-Speed}}(t,\tau)$, $\rho_{\text{LF-Radial}}(t,\tau)$, $\rho_{\text{LF-Angular}}(t,\tau)$ for different combinations of (t,τ). In panels a-c, each panel contains 231 points corresponding to 231 combinations of (t,τ) within the mobbing flock. **d-f**, The heatmap of difference of ρ as $\Delta\rho(t,\tau) = \rho_{\text{LF-MS}}(t,\tau) - \rho_{\text{LF-Speed}}(t,\tau)$, $\rho_{\text{LF-MS}}(t,\tau) - \rho_{\text{LF-Radial}}(t,\tau)$, $\rho_{\text{LF-MS}}(t,\tau) - \rho_{\text{LF-Angular}}(t,\tau)$. The gradient color ranging from red to white to blue in panels d-f is indicative of the value of $\Delta\rho \in [-2,2]$. **g**, We show the flocking trajectory from $[t-\tau, t]$ with the gradient background color scaling with $\Delta\rho = \rho_{\text{LF-MS}}(t,\tau) - \rho_{\text{LF-Speed}}(t,\tau)$. We observe that the $\Delta\rho(t,\tau)$ values indicated by the red background color consistently correspond to the process of collective turn. **h-j**, the 231 points are categorized into 3 parts: $|\Delta\rho| < 0.2$, $\Delta\rho > 0.5$ and $\Delta\rho < -0.5$. The y-axis of panels h-j records the corresponding average angular and nestedness.

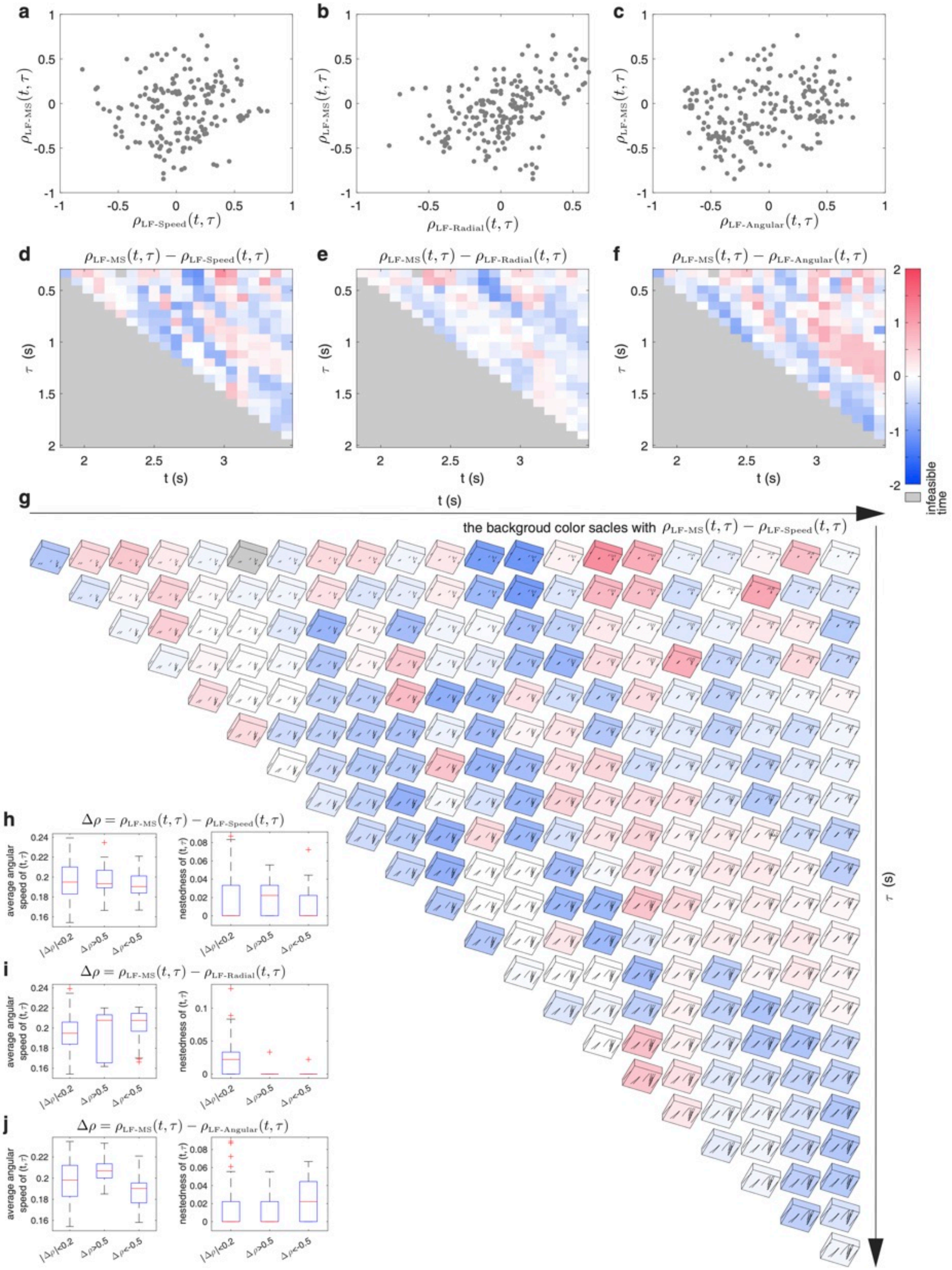

a
b
c
$\rho_{\text{LF-MS}}(t,\tau)$
$\rho_{\text{LF-Speed}}(t,\tau)$
$\rho_{\text{LF-Radial}}(t,\tau)$
$\rho_{\text{LF-Angular}}(t,\tau)$
d
e
f
$\rho_{\text{LF-MS}}(t,\tau) - \rho_{\text{LF-Speed}}(t,\tau)$
$\rho_{\text{LF-MS}}(t,\tau) - \rho_{\text{LF-Radial}}(t,\tau)$
$\rho_{\text{LF-MS}}(t,\tau) - \rho_{\text{LF-Angular}}(t,\tau)$
τ (s)
t (s)
infeasible time
g
t (s)
the backgroud color sacles with $\rho_{\text{LF-MS}}(t,\tau) - \rho_{\text{LF-Speed}}(t,\tau)$
τ (s)
h
$\Delta\rho = \rho_{\text{LF-MS}}(t,\tau) - \rho_{\text{LF-Speed}}(t,\tau)$
i
$\Delta\rho = \rho_{\text{LF-MS}}(t,\tau) - \rho_{\text{LF-Radial}}(t,\tau)$
j
$\Delta\rho = \rho_{\text{LF-MS}}(t,\tau) - \rho_{\text{LF-Angular}}(t,\tau)$
average angular speed of (t,τ)
nestedness of (t,τ)
$|\Delta\rho|$<0.2
$\Delta\rho$>0.5
$\Delta\rho$<-0.5

Supplementary Figure 32 | Illustration of comparison of correlation of LF-MS and LF-Speed for a transit flock. Here we use a transit flock (shown in Fig.1c of the main text) to show the workflow of comparison. **a-c**, we perform the Spearman correlation to yield $\rho_{\text{LF-MS}}(t,\tau)$ and $\rho_{\text{LF-Speed}}(t,\tau)$, $\rho_{\text{LF-Radial}}(t,\tau)$, $\rho_{\text{LF-Angular}}(t,\tau)$ for different combinations of (t,τ). In panels a-c, each panel contains 231 points corresponding to 231 combinations of (t,τ) within the transit flock. **d-f**, The heatmap of difference of ρ as $\Delta\rho(t,\tau) = \rho_{\text{LF-MS}}(t,\tau) - \rho_{\text{LF-Speed}}(t,\tau)$, $\rho_{\text{LF-MS}}(t,\tau) - \rho_{\text{LF-Radial}}(t,\tau)$, $\rho_{\text{LF-MS}}(t,\tau) - \rho_{\text{LF-Angular}}(t,\tau)$. The gradient color ranging from red to white to blue in panels d-f is indicative of the value of $\Delta\rho \in [-2,2]$. We find three kinds of $\Delta\rho$ is significantly lower than that of mobbing flock, because the color intensity in Supplementary Figure 32d-f is noticeably lighter compared to that in panels Supplementary Figure 31d-f. **g**, We show the flocking trajectory from $[t-\tau, t]$ with the gradient background color scaling with $\Delta\rho = \rho_{\text{LF-MS}}(t,\tau) - \rho_{\text{LF-Speed}}(t,\tau)$. **h-j**, the 231 points are categorized into 3 parts: $|\Delta\rho| < 0.2$, $\Delta\rho > 0.5$ and $\Delta\rho < -0.5$. The y-axis of panels h-j records the corresponding average angular and nestedness.

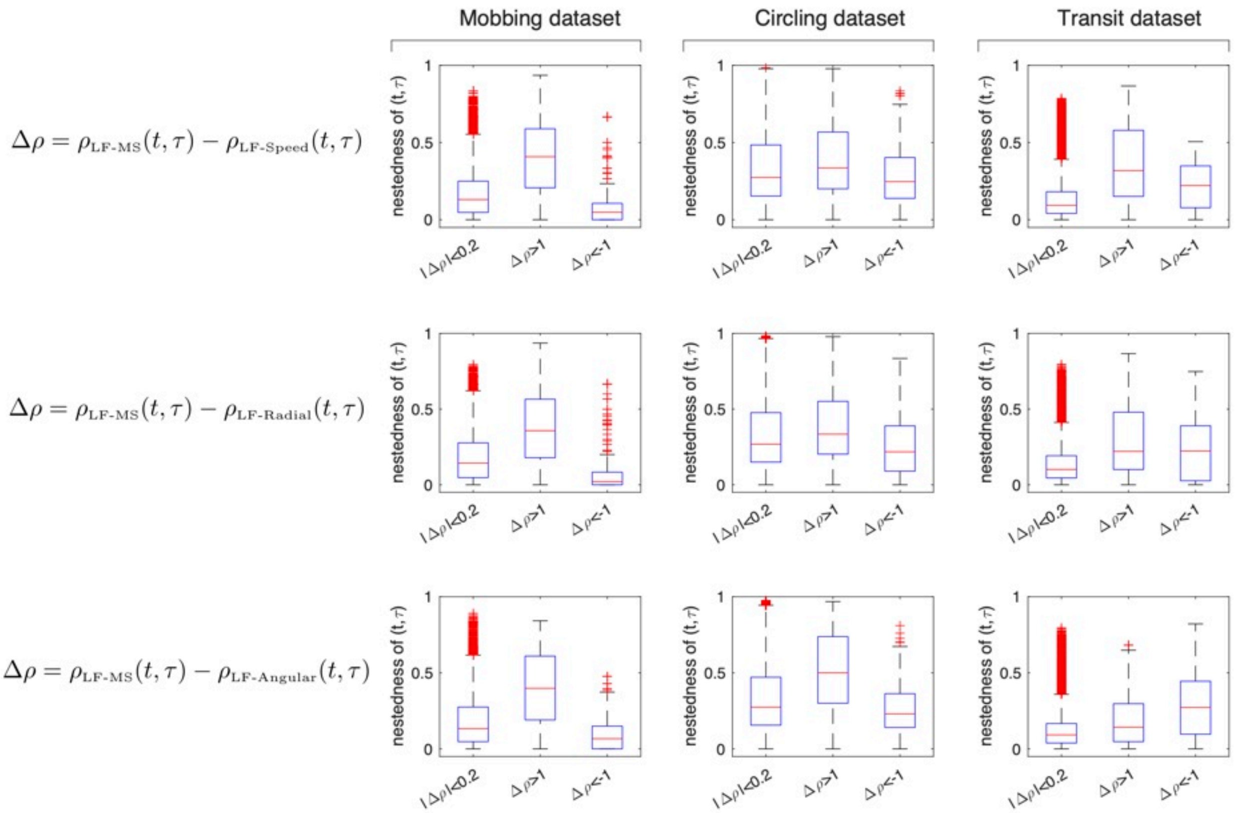

Supplementary Figure 33 | Comparison of correlation of LF-MS and LF-Speed for three flocking datasets. We extend the three kinds of $\Delta\rho$ to mobbing, circling and transit datasets. In mobbing and circling dataset, the sub-flocks with $\Delta\rho > 1$ exhibit the highest nestedness, while those sub-flocks with $\Delta\rho < -1$ display the lowest nestedness. The nestedness of sub-flocks with $|\Delta\rho| < 0.2$ are positioned in the middle. Note that the trend observed in mobbing and circling datasets is the same for three kinds of $\Delta\rho$. For the transit dataset, we could not observe the clear pattern between $\Delta\rho > 1$ and $\Delta\rho < -1$.

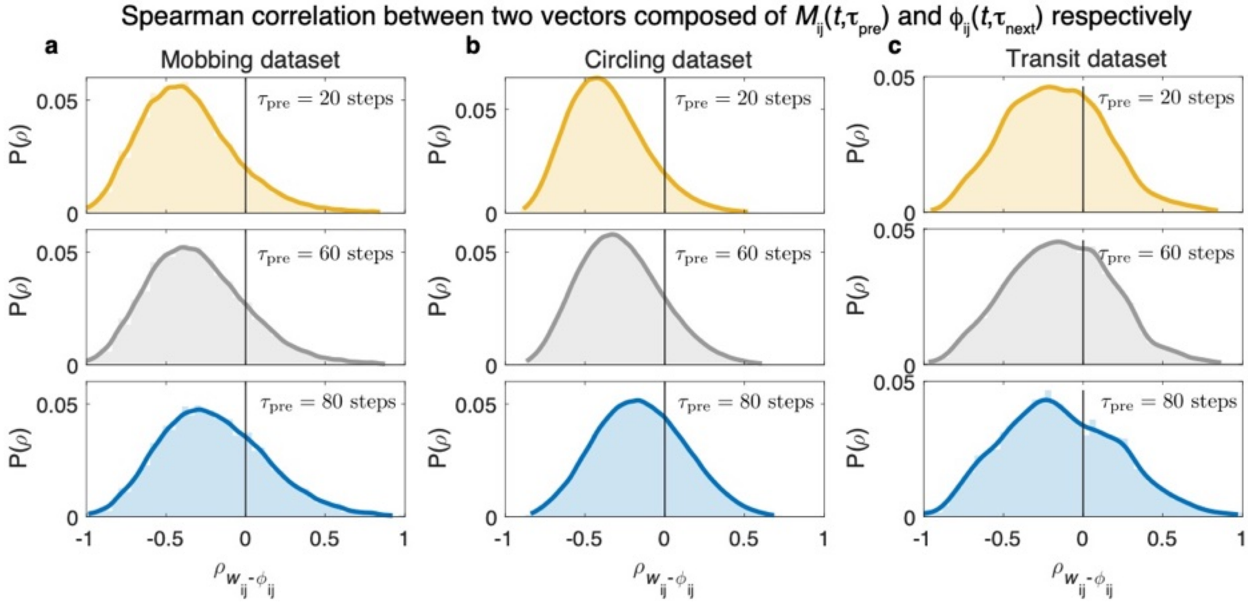

Supplementary Figure 34 | The correlation analysis between MS and average of temporal velocity consensus at the individual level. We use the flock within the period $[t-\tau_{\mathrm{pre}}, t]$ (highlighted by blue in a) and the period $[t, t+\tau_{\mathrm{next}}]$ to generate $M_{ij}(t,\tau_{\mathrm{pre}})$ (and the normalization $w_{ij}(t,\tau_{\mathrm{pre}}) = \frac{M_{ij}(t,\tau)}{\sum M_{ij}(t,\tau)}$) and the average of temporal velocity consensus $\phi_{ij}(t,\tau_{\mathrm{next}}) = \langle \mathrm{v}_1(t) \cdot \mathrm{v}_j(t) \rangle$. We extend the correlation analysis between $w_{ij}(t,\tau_{\mathrm{pre}})$ and $\phi_{ij}(t,\tau_{\mathrm{next}})$ across different combinations of t and τ to mobbing (**a**), circling (**b**) and transit (**c**) datasets. In this figure we take $\alpha = 0$ to calculate MS.

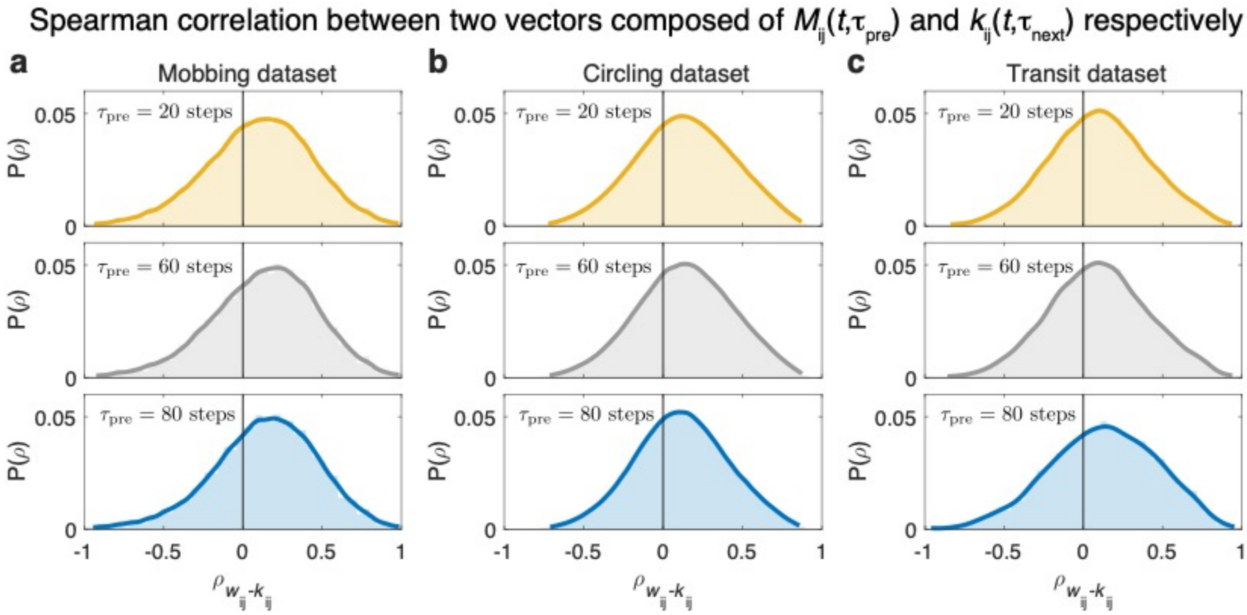

Supplementary Figure 35 | The correlation analysis between $w_{ij}(t, \tau_{pre})$ and $k_{ij}(t, \tau_{next})$ across different combinations of t and τ for three datasets. The correlation analysis, conducted on mobbing (**a**), circling (**b**) and transit (**c**) datasets, is same with Fig.4h-j in the main text, except that we take $\alpha = 1$ to calculate MS in this figure.

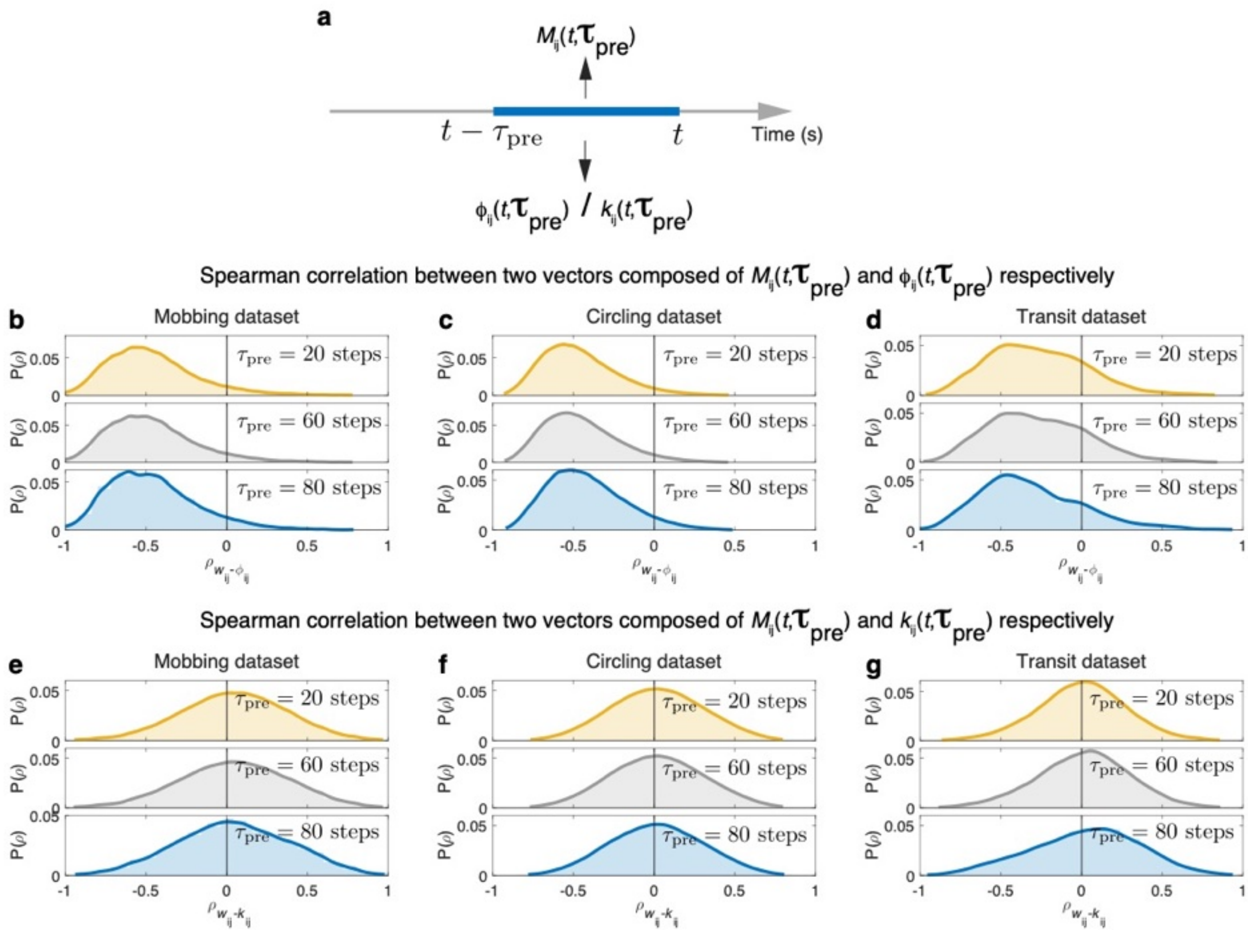

Supplementary Figure 36 | The correlation analysis between two vectors composed of $\boldsymbol{M_{ij}}$ and $\boldsymbol{\phi_{ij}}$ (or $\boldsymbol{k_{ij}}$) from the same period $[\boldsymbol{t-\tau_{\mathrm{pre}}, t}]$. a, Different with Fig.4 in the main text, both w_{ij} and k_{ij} are derived from the same period of $[t-\tau_{\mathrm{pre}}, t]$. In three flocking datasets, $\phi_{ij}(t,\tau_{\mathrm{pre}})$ continues to exhibit a predominance of negative correlation with $w_{ij}(t,\tau_{\mathrm{pre}})$ during the same period (**b-d**). However, unlike the emergence of positive correlations between $w_{ij}(t,\tau_{\mathrm{pre}})$ and $k_{ij}(t,\tau_{\mathrm{next}})$ in a sequential period, we could not observe a prevalence of positive correlations between $w_{ij}(t,\tau_{\mathrm{pre}})$ and $k_{ij}(t,\tau_{\mathrm{pre}})$ (**e-g**). In this figure we take $\alpha = 0$ to calculate MS.

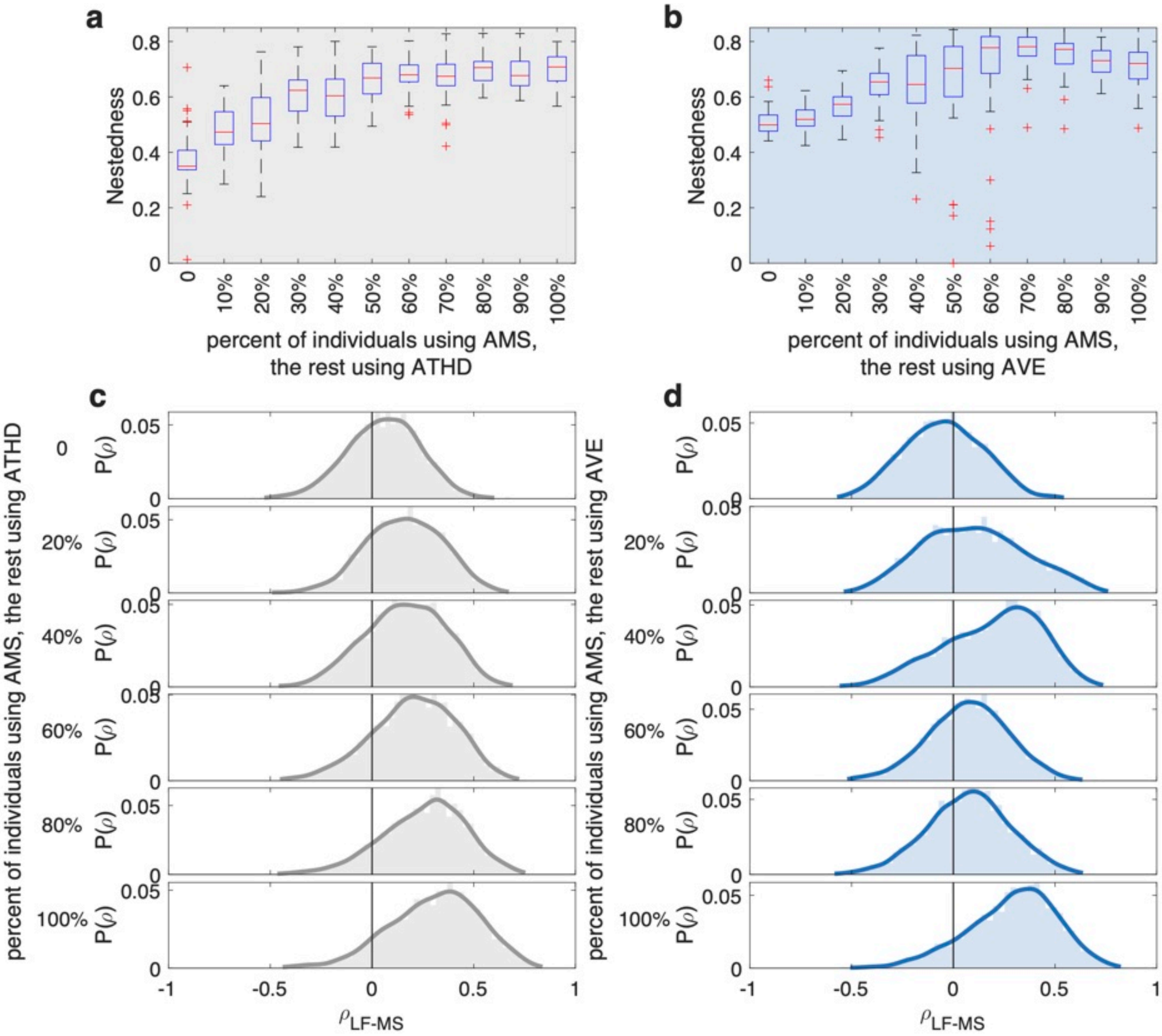

Supplementary Figure 37 | The hybrid simulations by introducing AMS to ATHD or average interaction. The simulations follow the setting of Fig.5 in the main text. Firstly we set all the individuals follow ATHD (or average interaction), and then we tune the percent of individual utilizing AMS form 0% to 100%, such as, x-axis of a,b. Then we compare the nestedness of LF networks (**a,b**) and distribution of LF-MS correlation (**c,d**) across the different percent of individual using AMS.

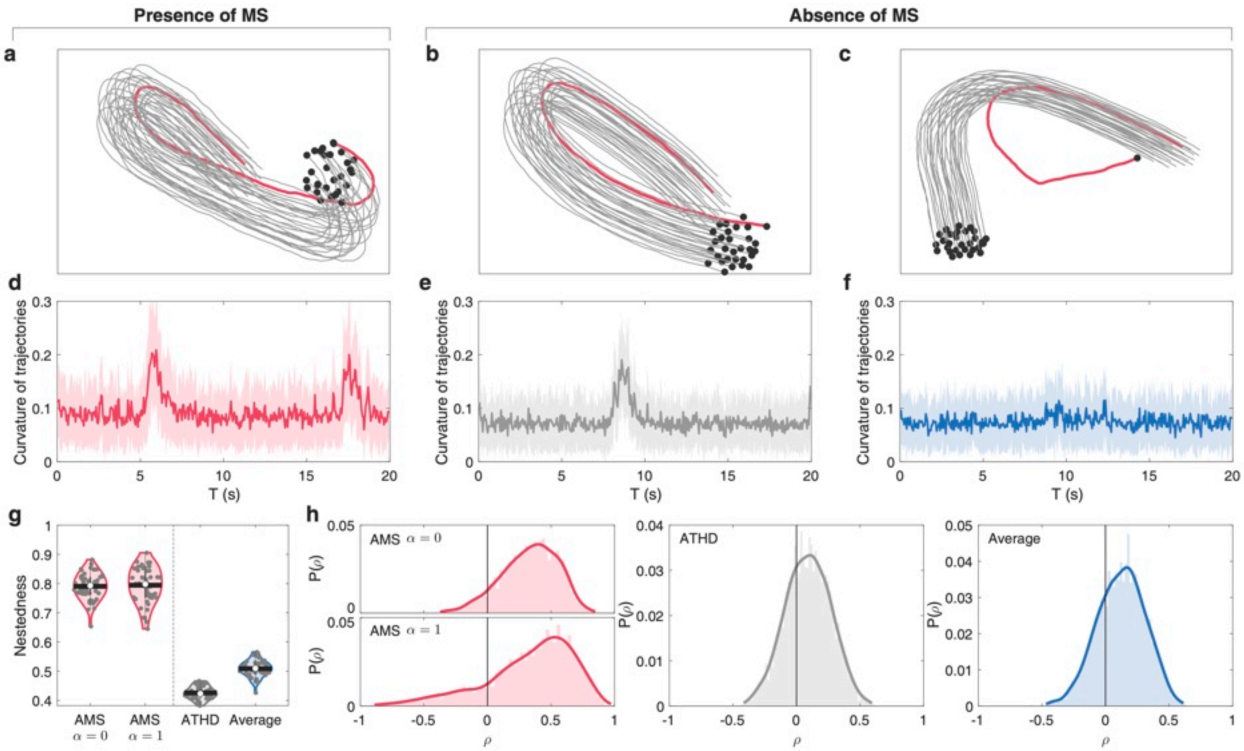

Supplementary Figure 38 | Comparison of self-propelled particle model in presence or absence of MS in 2D. The panel layout is identical to Fig. 4 in the main text, with the only distinction being that the results in this figure are derived from 2D data. We use a self-propelled particle model that particles follow the local interaction rules, i.e., AMS (**a**), ATHD (**b**) and average interaction (**c**). The additional potential well, imposed on one of individuals (red trajectories), aims to lead the flock come back to origin. The swarm size is 30 particles. The black points represent the end of trajectories. In panel a, the flocking trajectories are generated by AMS with $\alpha = 0$. **d-f**, The temporal curvature of flock trajectories respectively shown in panels a-c. In panels d-f, the solid curves represent the average of trajectory curvatures from 30 particles, and the shadow area represents the standard deviation (SD). **g**, The distribution of nestedness of LF networks derived from flocking trajectories using AMS, ATHD and average interaction. The white points (or black lines) represent the median (or mean) value. **h**, The distribution of Spearman correlation (ρ) between LF and MS over different combinations of t and τ from flocking trajectories using AMS, ATHD and average interaction. For each flock we take 10 time stamps for t and 11 time points for τ to perform the correlation analysis between LF and MS. In panel g and h, we run 50 independent simulations for each interaction type.

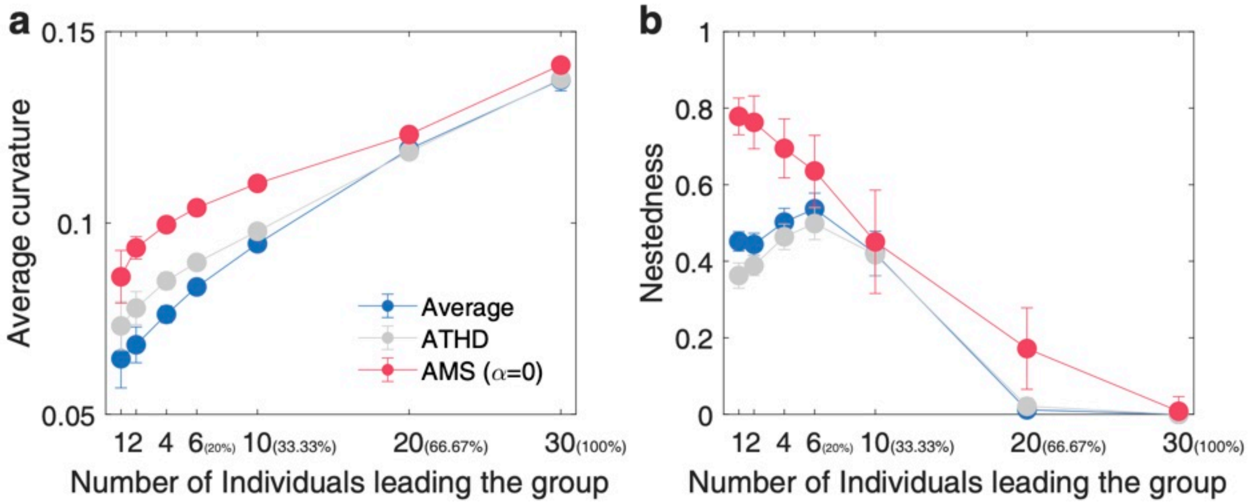

Supplementary Figure 39 | A tunable parameter of the number of individuals leading the group in the simulation results of Fig.4. The simulation results are generated by the same setting and parameters with Fig.4 except the number of individuals leading the group. The curves of average curvature of flocking trajectory (**a**) and nestedness (**b**) as a function of the number of individuals leading the group. The percent in the parenthesis indicates the proportion of informed agent to lead the group. The error bar represents the standard deviation (SD) calculated from 50 independent simulations.

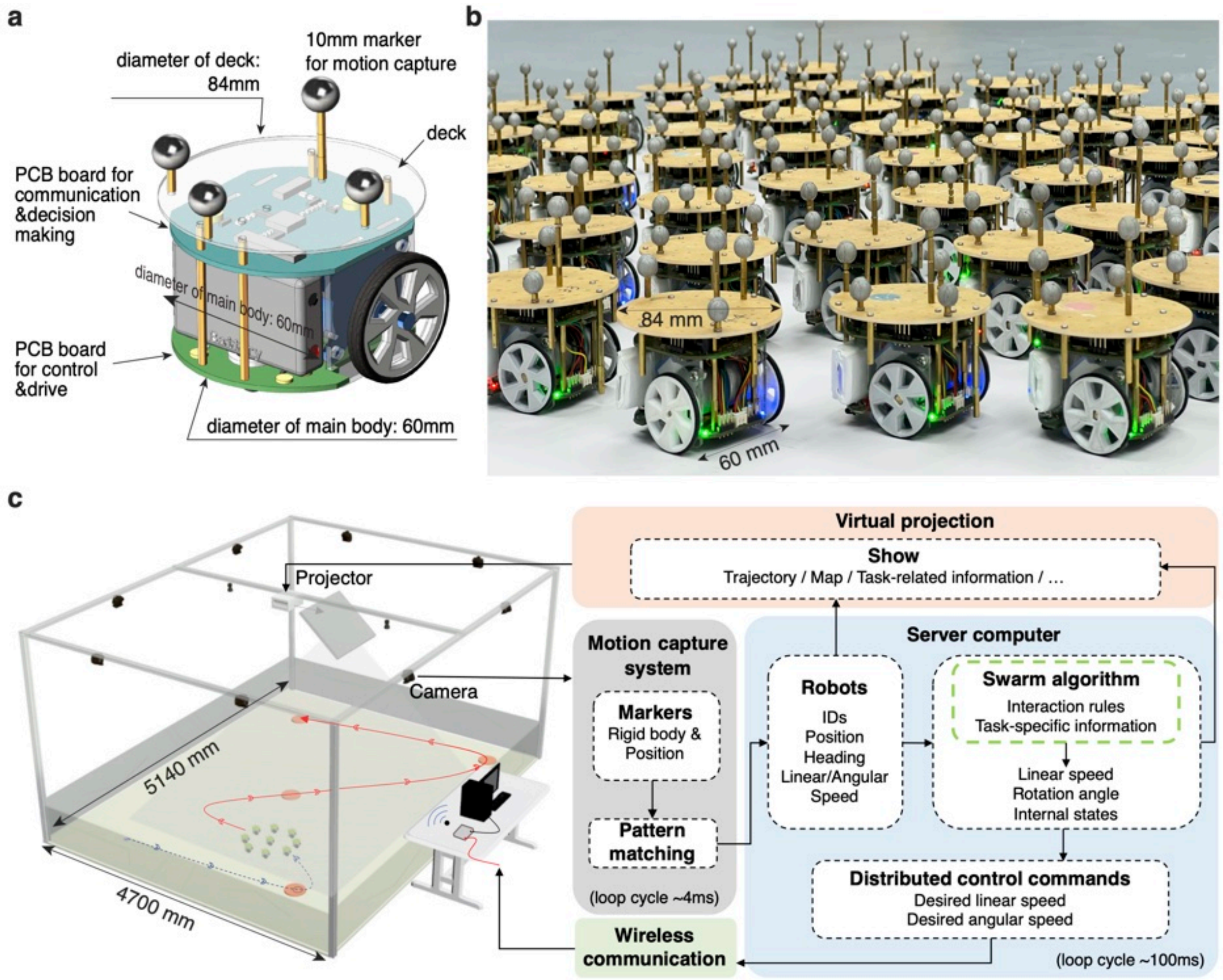

Supplementary Figure 40 | Experimental set-up of swarm robotics system. a, Overview of the miniature two-wheel differential mobile robot used for swarm experiments. The diameter of robots' main body is 60mm and the deck at top to place markers for localization is 84mm. **b**, The system could support the swarm size of $\sim 10^2$ magnitudes of miniature robots. Due to experimental arena constraints, the maximum of 50 robots are used for swarm experiments in this work. **c,** Illustration of the experimental set-up and the environment configuration. A ground control station, equipped with a radio transmitter, computes and sends run-time control commands to the robots. The swarm robotics system uses motion capture to locate the position of each robot.

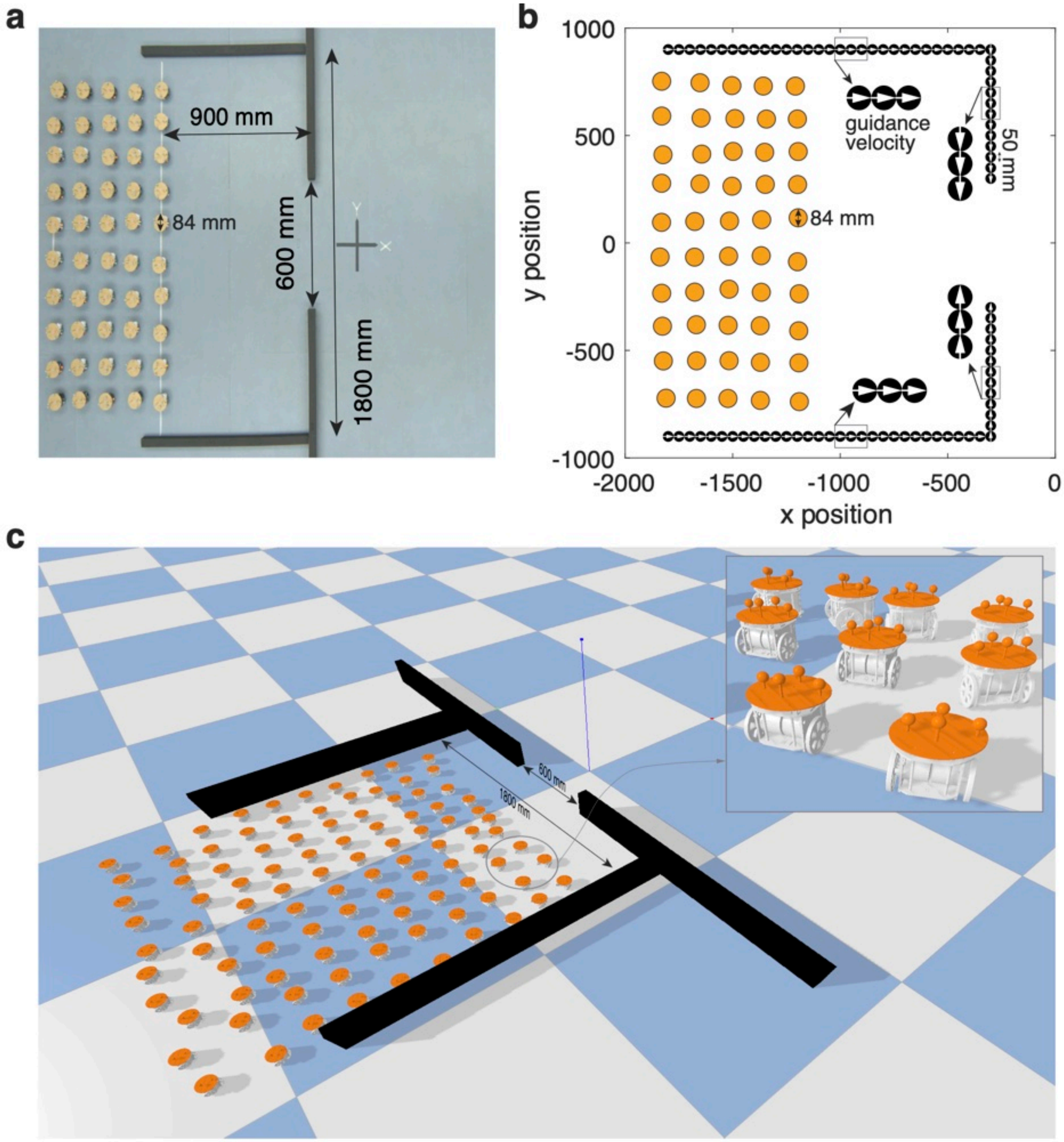

Supplementary Figure 41 | Experimental set-up and semi-physical simulation of the collective evacuation. a, The swarm of 50 robots for collective evacuation experiment (the narrow exit of 600mm) are initially distributed far from the exit with grid layout. **b,** To ensure the goal-oriented swarm evacuation in a confined environment, we assume that each virtual cylinder in the wall could indicate the exit direction according to their location. See Methods in main text for detailed information about the swarm model of collective evacuation. **c**, To perform the swarm experiments with hundreds of robots, we transfer the real robots to semi-physical simulation with the same motion characteristic in Pybullet[11].

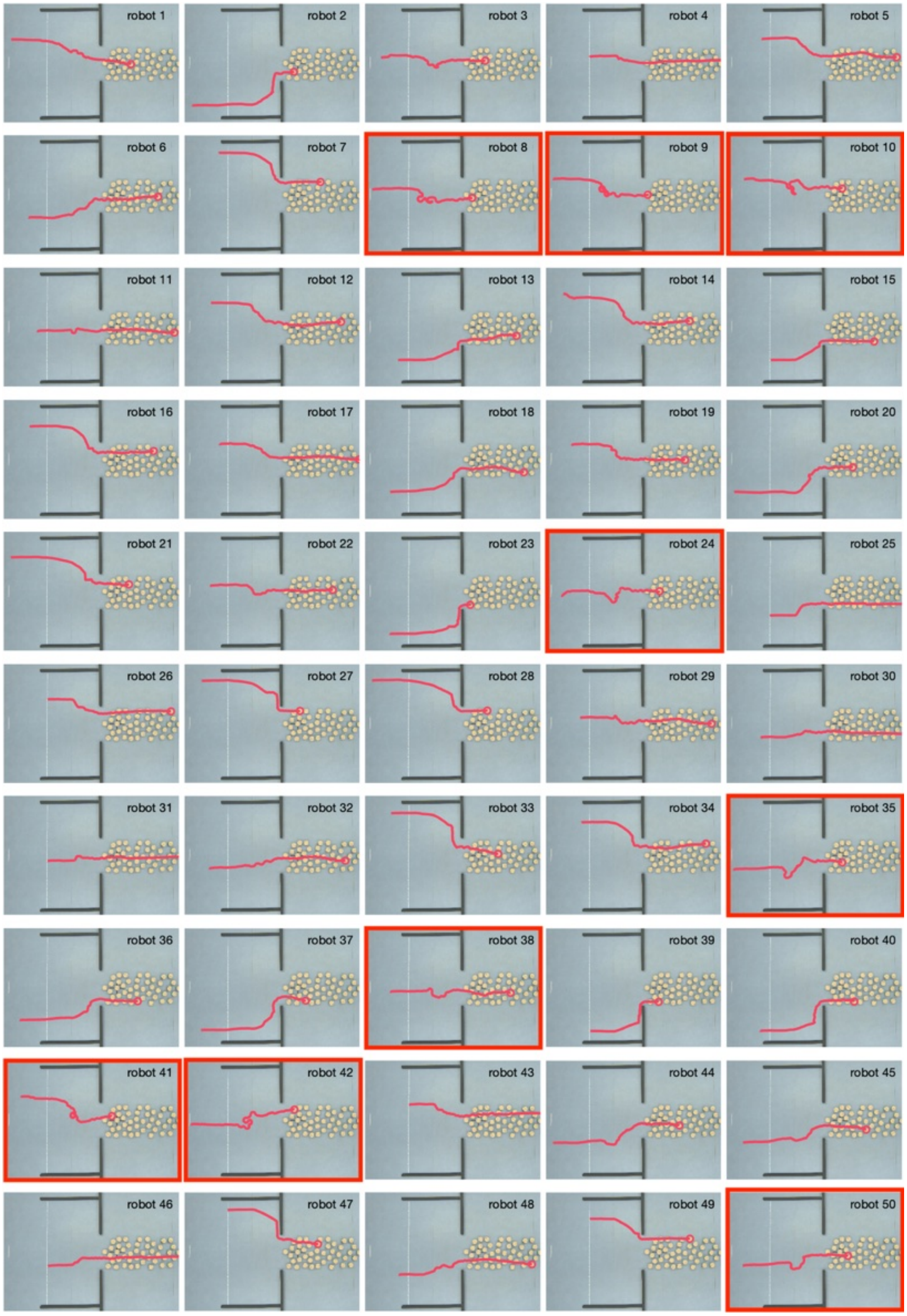

Supplementary Figure 42 | Trajectory of each robot in collective evacuation experiment shown in Fig.6c of the main text. This experiment is performed by AMS-100%. The swarm size is 50 robots. The red boxes are used to highlight some individuals sacrificing their spending time to temporarily move towards the opposite direction of the exit.

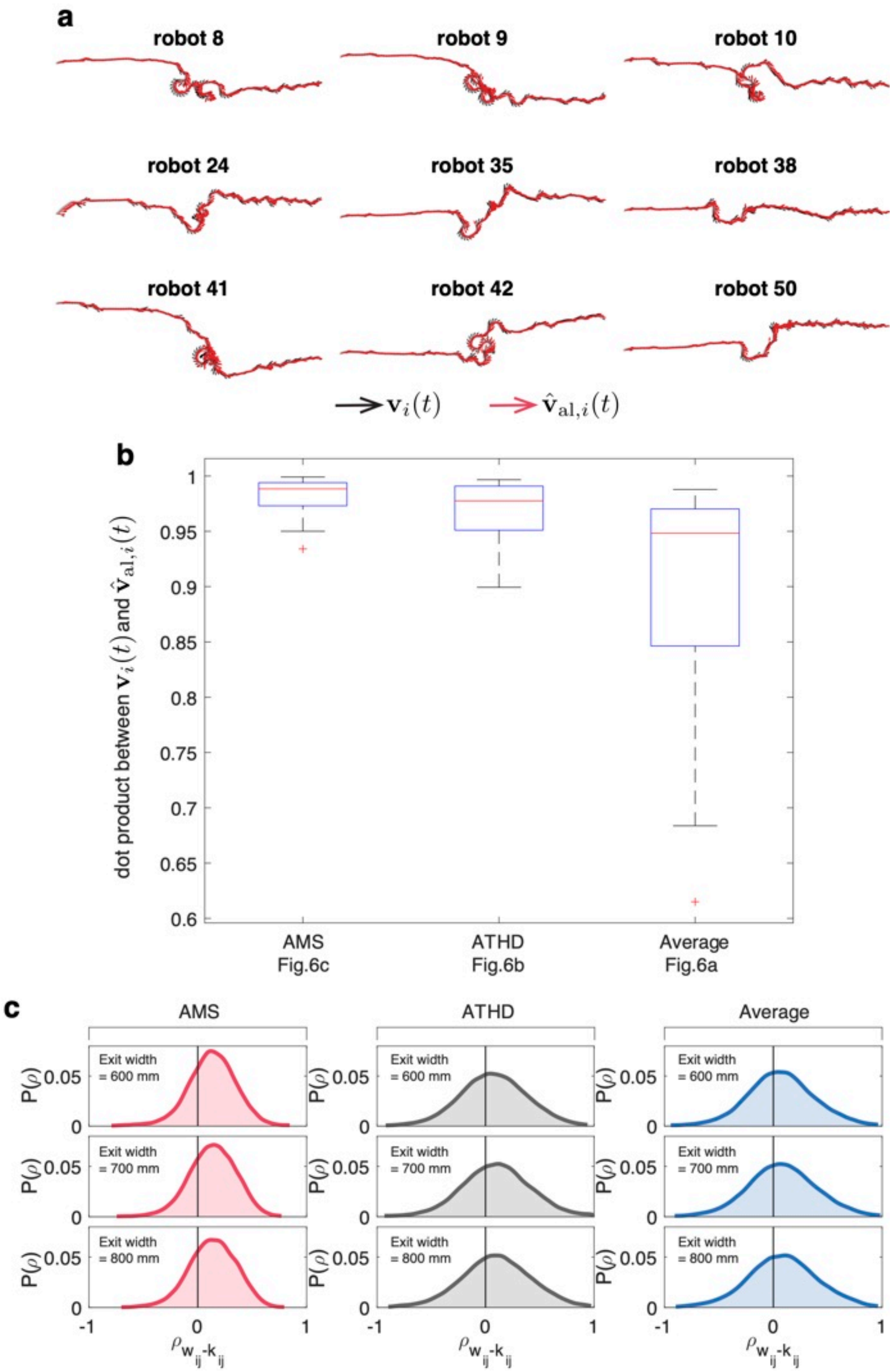

Supplementary Figure 43 | The individuals moving in the opposite direction of the exit in experiment of Fig.6c. a, To gain a better understanding of the self-organized mechanism of AMS and why certain individuals (highlighted by red boxes in Supplementary Figure 42) are moving in the opposite direction of the exit, we present three types of information about these individuals: trajectory, $\mathbf{v}_i(t)$ (black arrow), and $\hat{\mathbf{v}}_{\mathrm{al},i}$ (red arrow). **b**, We compare the dot product between $\mathbf{v}_i(t)$ and $\hat{\mathbf{v}}_{\mathrm{al},i}(t)$ for the results of AMS, ATHD and Average shown in Fig.6a-c. We find the dot product between $\mathbf{v}_i(t)$ and $\hat{\mathbf{v}}_{\mathrm{al},i}(t)$ in descending order is AMS, ATHD and Average, and AMS has the least variance of dot product.

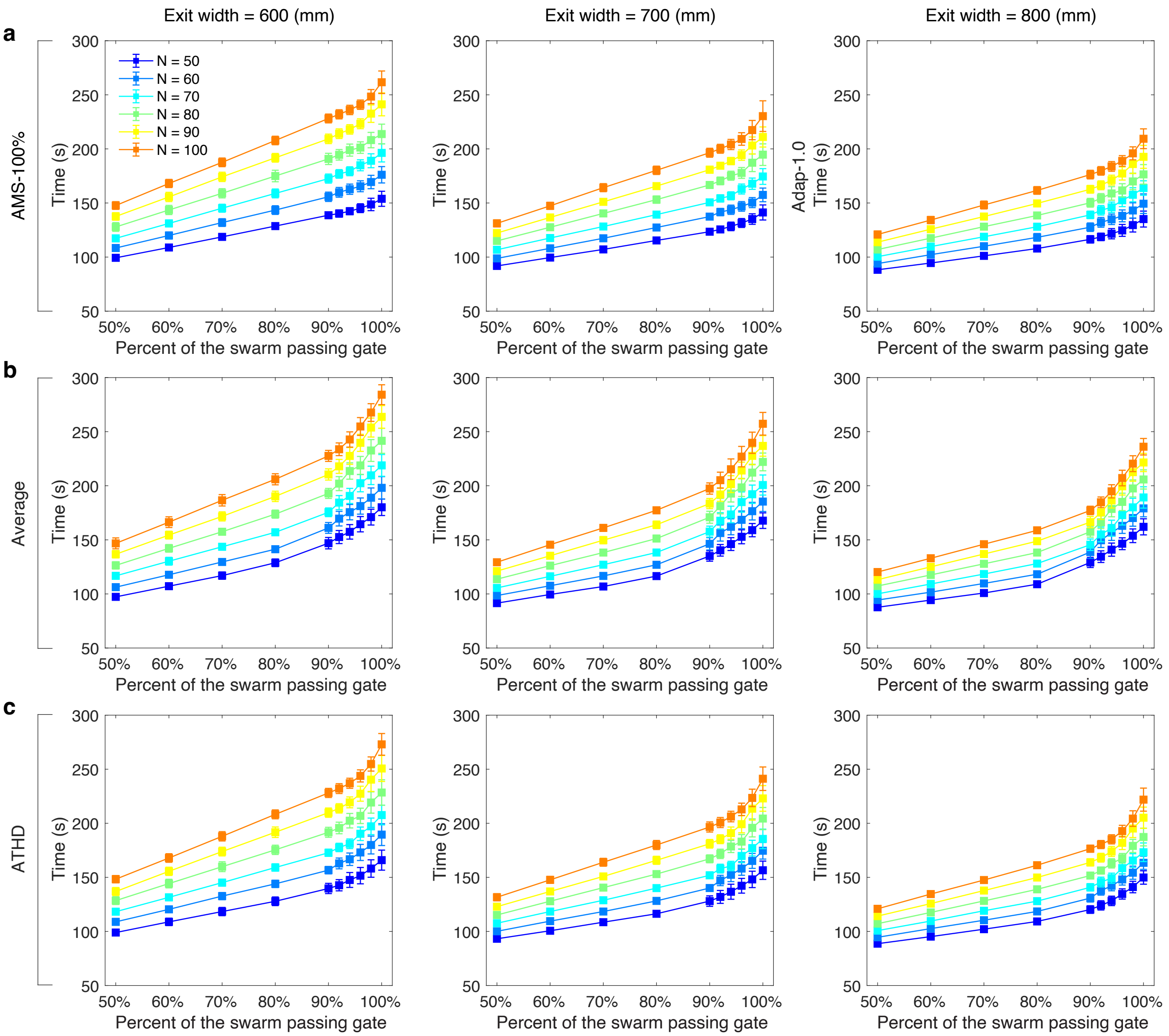

Supplementary Figure 44 | The spending time as a function of percent of the swarm passing gate from the semi-physical simulations. We run the semi-physical simulations with the alignment interaction of AMS-100% (**a**), Average (**b**) and ATHD (**c**). The error bar represents the standard deviation (SD) from 50 independent simulations. The parameters in semi-physical simulations of collective evacuation are the same with Fig.6 of the main text.

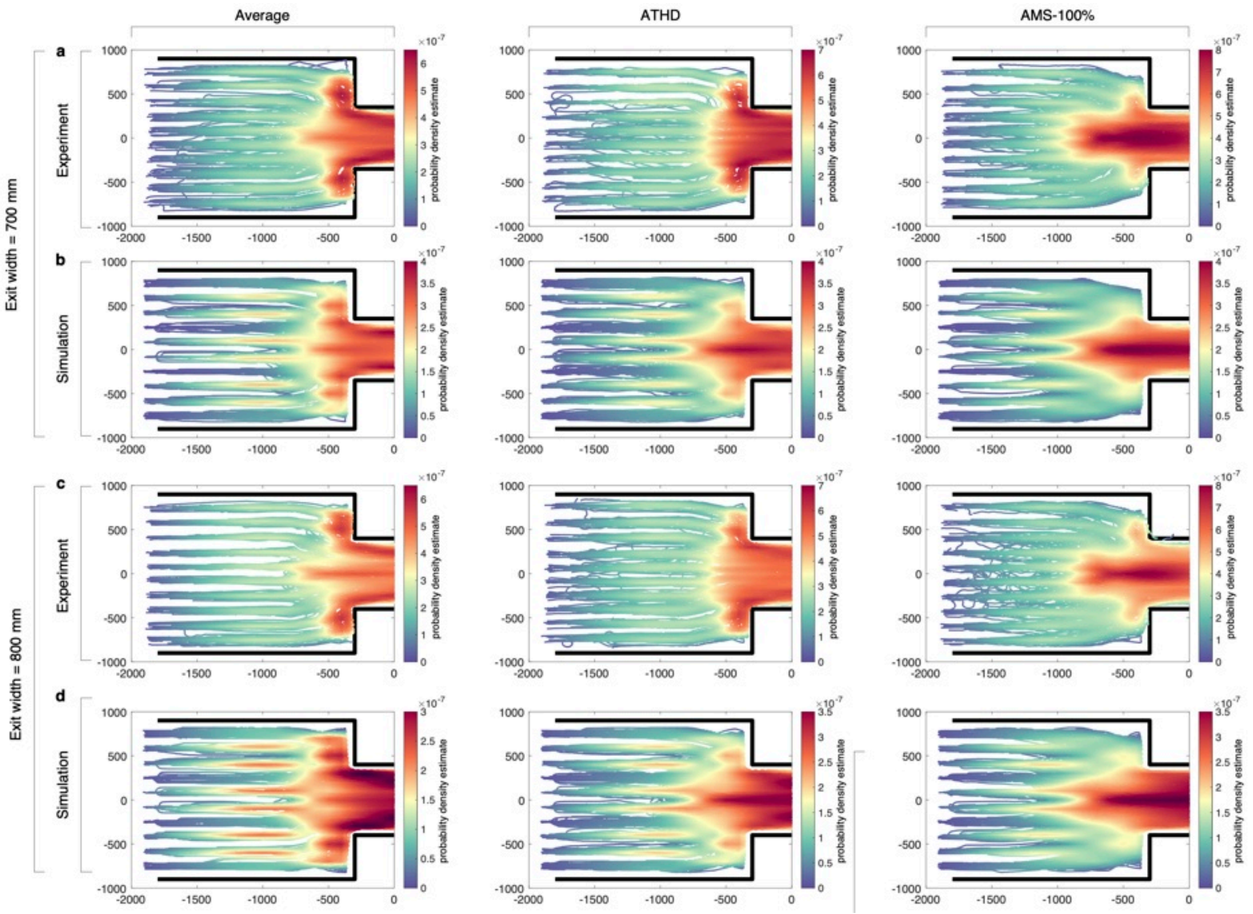

Supplementary Figure 45 | The statistics of historical trajectories of all robots in collective evacuation experiments or simulations under the exit width of 700mm and 800mm. The statistics of historical trajectories of all 50 robots in 10 independent experiments (**a,c**) and 50 independent simulations (**b,d**). The color scales correspond to the probability density estimate of historical trajectories. The black line indicates the wall. The swarm size is 50 in the experiments and semi-physical simulations. The parameters in the experiments or semi-physical simulations of collective evacuation are the same with Fig.6 of the main text.

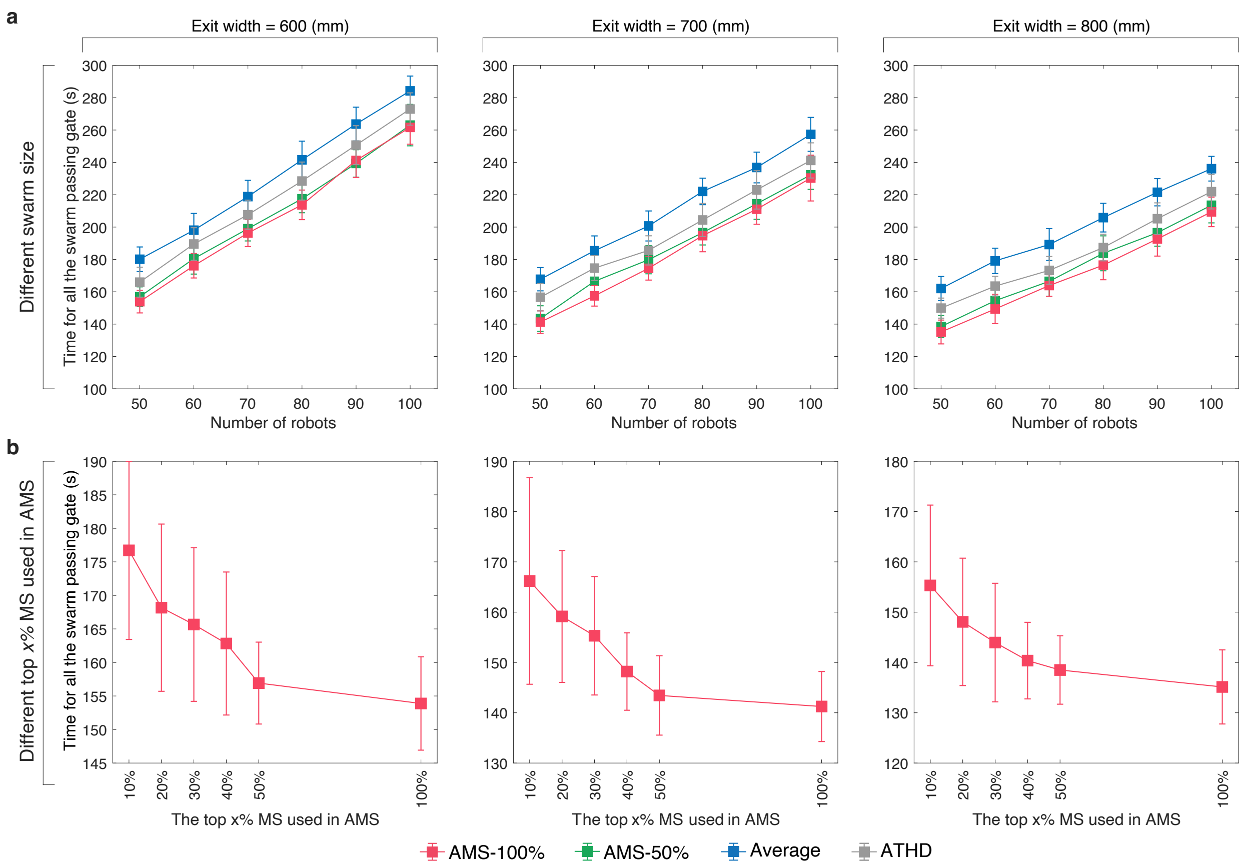

Supplementary Figure 46 | The spending time of all the swarm evacuating the narrow exit as a function of swarm size and the top x% used in AMS from the semi-physical simulations. **a,** The evacuation time as a function of swarm size. Here AMS involves 100% MS. **b,** The evacuation time as a function of top x% used in AMS. Here the swarm size is 50. In panel a,b, the error bar represents the standard deviation (SD) calculated from 50 independent semi-physical simulations. The parameters in semi-physical simulations of collective evacuation are the same with Fig.6 of the main text.

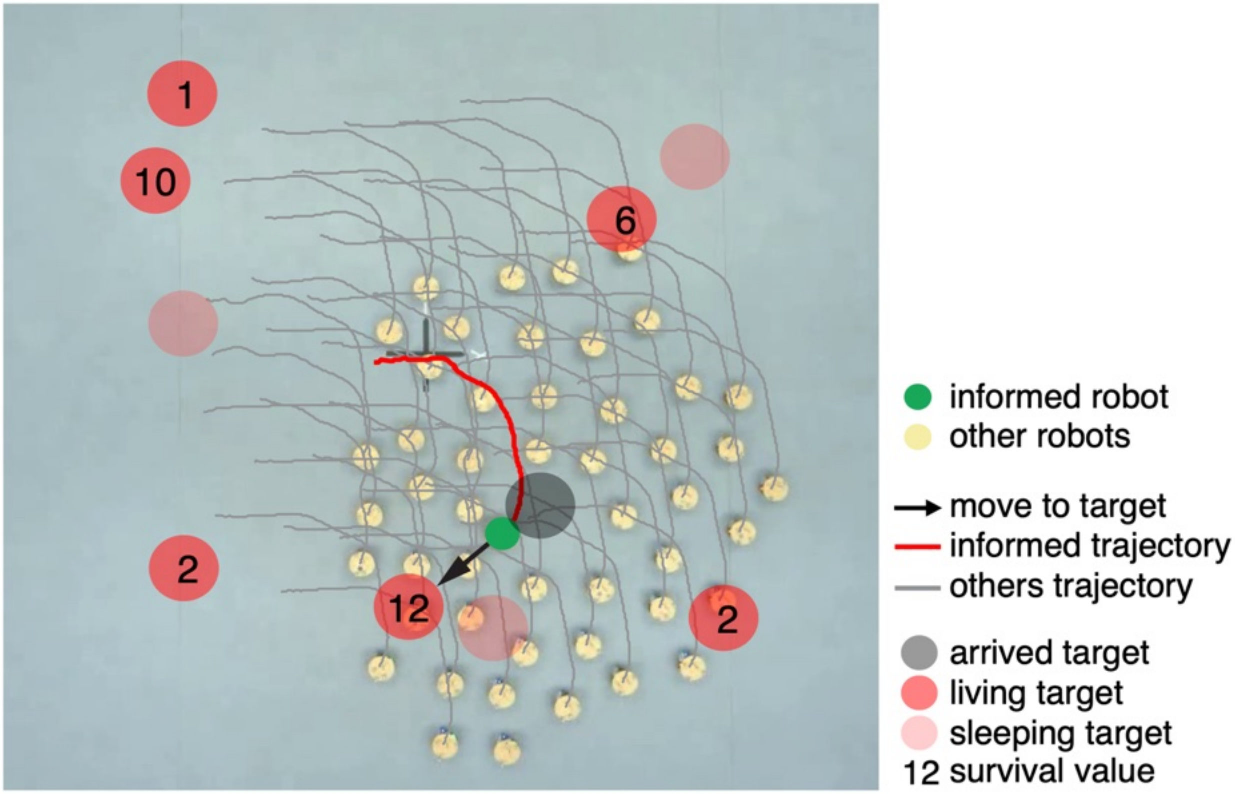

Supplementary Figure 47 | The snapshot of collective following experiments. A snapshot from the top view to show that an informed robot (green circle) moves to the nearest living target and the others (yellow circles) follow. The 10 targets are randomly distributed at the experiment arena. The target's living, sleeping or arrived states are highlighted by dark red, light red and dark grey, respectively. The number in the targets represents the survival value. See Supplementary Note 9.1 for detailed information about how the informed robot moves according to the frequent switch of target states.

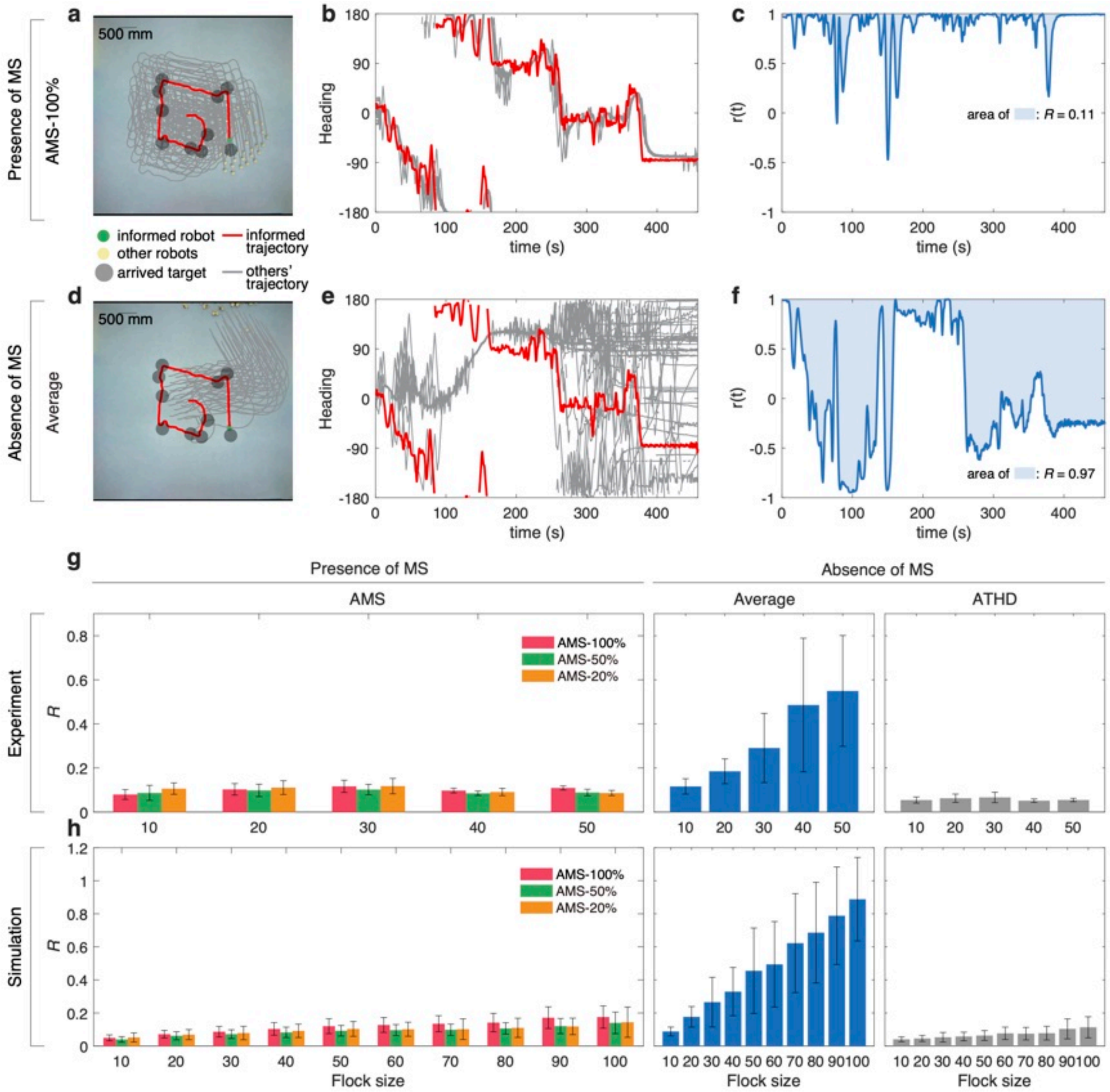

Supplementary Figure 48 | Collective following experiments. a, The swarm consisted of 50 robots use AMS to perform the collective following experiment. **b**, Following the informed robots' movements (red curve), the rest of swarm could promptly respond to stimuli from neighbors and maintain the swarm move together. **c**, The temporal collective response $r(t)$ for the experment of panel a. AMS makes the cumulative evaluation of collective response R approach to about 0.11 (equals to the area of light bule zone). **d-f**, For the same experiment setting of panel a, i.e., the movement of informed robot, the swarm using average interaction could not respond to heading changes of informed robot in time and totally fail to execute collective following. **g,** R as a function of swarm size up to 50 robots in experiments for different interaction types. **h,** In the simulations with the same robot's motion characteristic, R as a function of swarm size up to 100 robots for different interaction types. Here AMS-x% represents the focal robot only adaptively aligns with those neighbors who cumulatively possess the top x% MS. Note that ATHD could be the ideal condition of responding to neighbors' perturbations because the individual could be able to immediately and adaptively alter the influences from neighbors based on heading difference. In panels g,h, the error bar represents the standard deviation (SD) calculated from 10 independent experiments and 100 independent simulations.

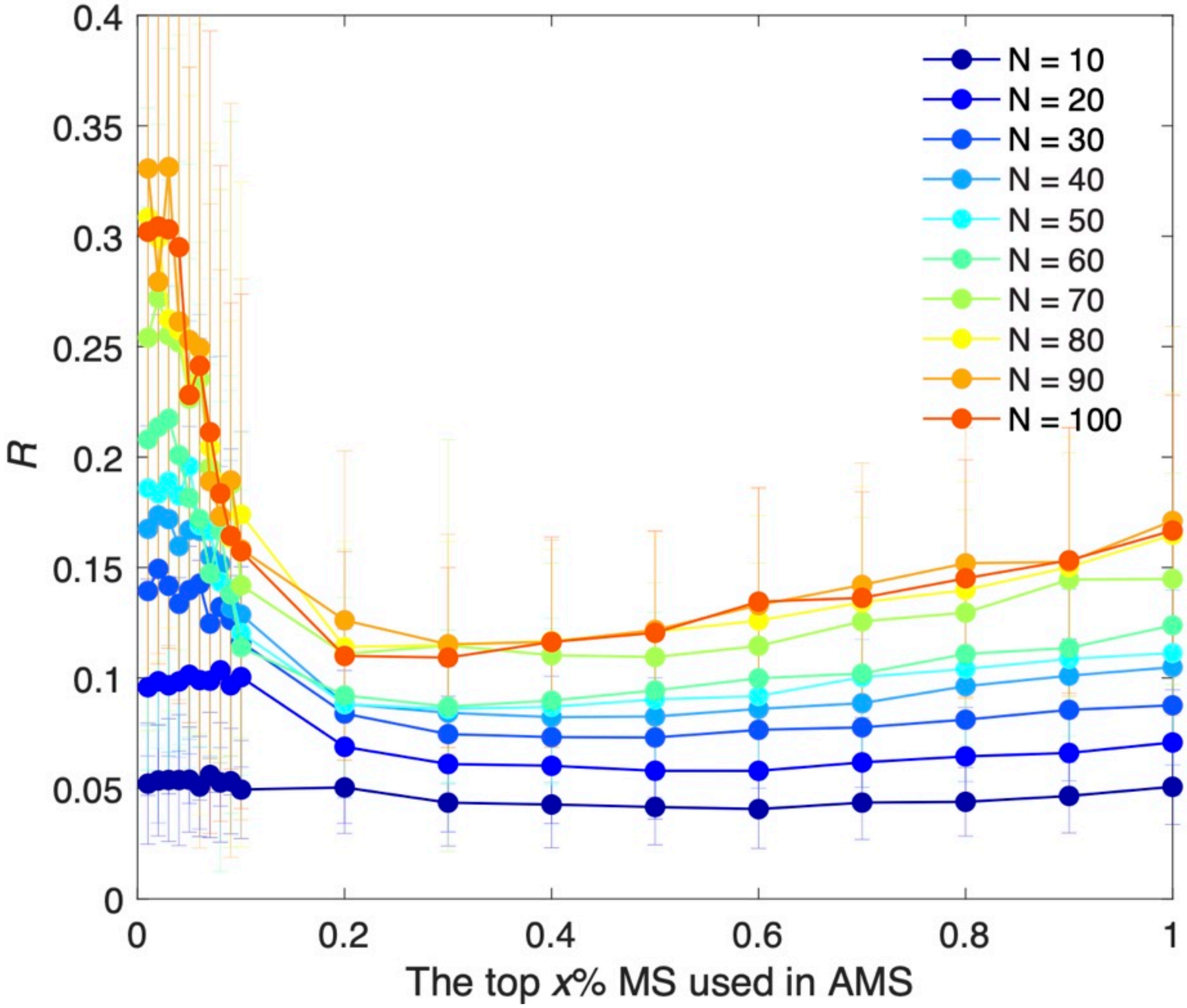

Supplementary Figure 49 | The performance of collective following as a function of swarm size and the top $x\%$ used in AMS. The results are generated by simulations with the same of robot's physical characteristic. The error bar represents the standard deviation (SD) calculated from 100 independent simulations. The parameters in simulations of collective following are the same with Fig.5 of the main text.

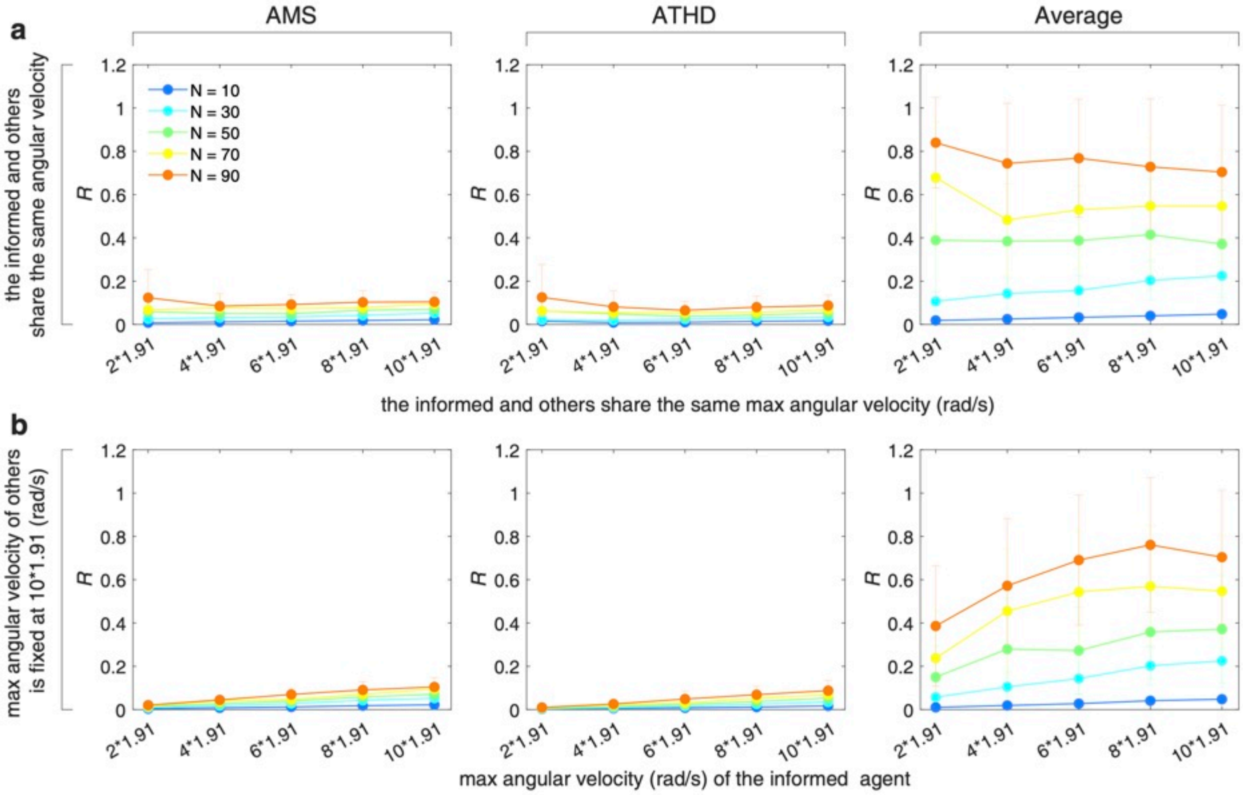

Supplementary Figure 50 | Consider the restricted angular velocity in the simulation of collective following. We set two kinds of restricted angular velocity in collective following simulations: (**a**) the informed agent and others share the same max angular velocity, (**b**) max angular velocity of the others is fixed at 10*1.91 rad/s but we change the informed agent with different max angular velocity. The error bar represents the standard deviation (SD) calculated from 50 independent simulations.

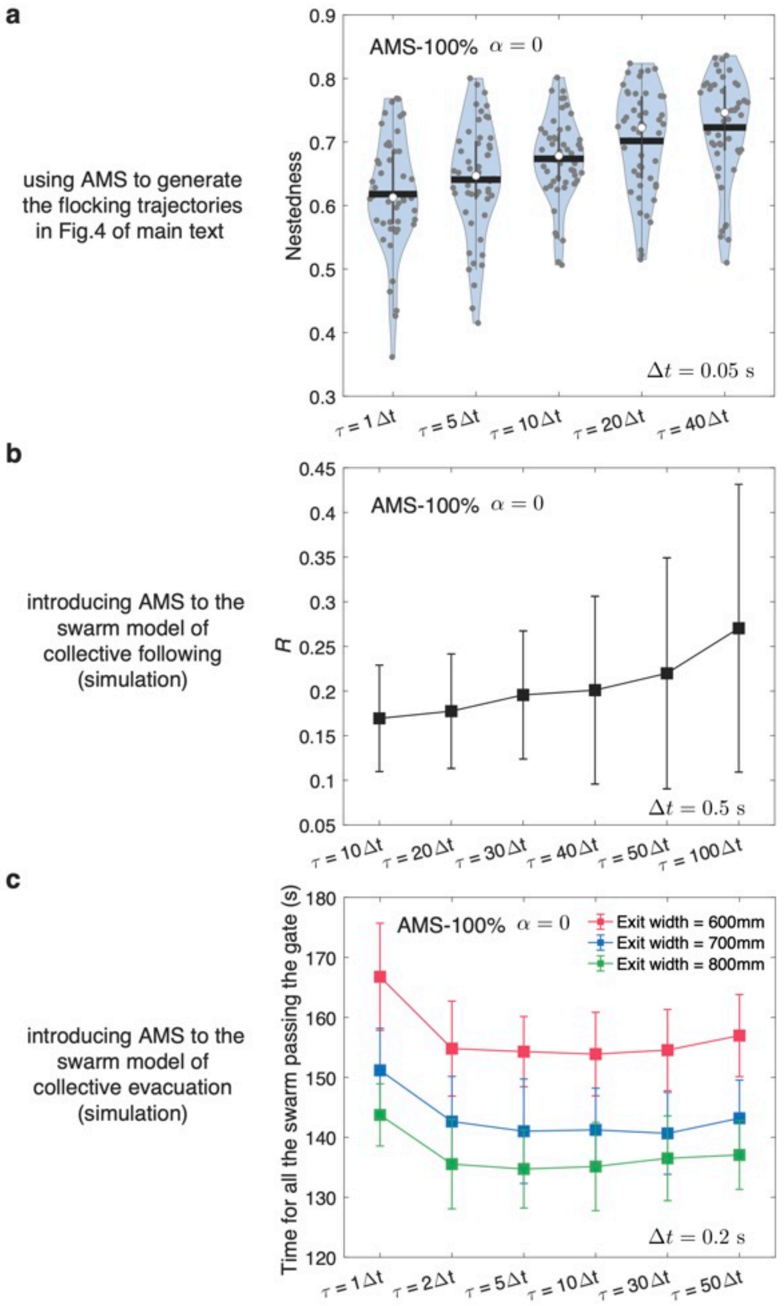

Supplementary Figure 51 | The effect of perceiving time (τ) on AMS-100%. a, The nestedness of LF networks as a function of τ. The flocking trajectories are generated by AMS with the same parameters used in Fig.4 of main text. We run 50 independent simulations for each τ. The white points (or black lines) represent the median (or mean) value. **b**, The performance of collective following R as a function of τ. The parameters in simulations of collective following are the same with Fig.5 of the main text. The error bar represents the standard deviation (SD) calculated from 100 independent simulations. **c**, The spending time for all the swarm passing the narrow exit as a function of τ. The parameters in semi-physical simulations of collective evacuation are the same with Fig.6 of the main text. The error bar represents the standard deviation (SD) calculated from 50 independent semi-physical simulations.

Supplementary References

1. Ling, H. *et al.* Behavioural plasticity and the transition to order in jackdaw flocks. *Nat Commun* **10**, 5174 (2019).
2. Evangelista, D. J., Ray, D. D., Raja, S. K. & Hedrick, T. L. Three-dimensional trajectories and network analyses of group behaviour within chimney swift flocks during approaches to the roost. *Proc. R. Soc. B.* **284**, 20162602 (2017).
3. Ling, H. *et al.* Simultaneous measurements of three-dimensional trajectories and wingbeat frequencies of birds in the field. *Journal of The Royal Society Interface* **15**, 20180653 (2018).
4. Unity Real-Time Development Platform, the 3D game engine. https://unity.com/
5. BiMat, a Matlab code package to calculate the modularity and nestedness of complex networks. http://bimat.github.io/
6. Papadopoulou, M., Hildenbrandt, H. & Hemelrijk, C. K. Diffusion during collective turns in bird flocks under predation. *Frontiers in Ecology and Evolution* **11**, (2023).
7. Almeida-Neto, M., Guimarães, P., Guimarães Jr, P. R., Loyola, R. D. & Ulrich, W. A consistent metric for nestedness analysis in ecological systems: reconciling concept and measurement. *Oikos* **117**, 1227–1239 (2008).
8. Staniczenko, P. P. A., Kopp, J. C. & Allesina, S. The ghost of nestedness in ecological networks. *Nat Commun* **4**, 1391 (2013).
9. Payrató-Borràs, C., Hernández, L. & Moreno, Y. Measuring nestedness: A comparative study of the performance of different metrics. *Ecology and Evolution* **10**, 11906–11921 (2020).
10. Pettit, B., Ákos, Z., Vicsek, T. & Biro, D. Speed Determines Leadership and Leadership Determines Learning during Pigeon Flocking. *Current Biology* **25**, 3132–3137 (2015).
11. Bullet Real-Time Physics Simulation. https://pybullet.org/
12. Detrain, C. & Deneubourg, J.-L. Collective Decision-Making and Foraging Patterns in Ants and Honeybees. in *Advances in Insect Physiology* (ed. Simpson, S. J.) vol. **35** 123–173 (Academic Press, 2008).
13. Talamali, M. S. *et al.* Sophisticated collective foraging with minimalist agents: a swarm robotics test. *Swarm Intell* **14**, 25–56 (2020).
14. Rahmani, P., Peruani, F. & Romanczuk, P. Flocking in complex environments—Attention trade-offs in collective information processing. *PLOS Computational Biology* **16**, e1007697 (2020).
15. Talamali, M. S., Saha, A., Marshall, J. A. R. & Reina, A. When less is more: Robot swarms adapt better to changes with constrained communication. *Sci. Robot.* **6**, eabf1416 (2021).
16. Zheng, Z., Lei, X. & Peng, X. Selective interaction and its effect on collective motion. *Sci Rep* **12**, 8601 (2022).
