# Supplementary material for: Perception of motion salience shapes the emergence of collective motions
Source: Nat Commun. 2024 Jun 5;15:4779. doi: 10.1038/s41467-024-49151-x (PMC11153630; doi:10.1038/s41467-024-49151-x)
Supplement: Supplementary file 3 — Description Of Additional Supplementary File [file 41467_2024_49151_MOESM3_ESM.pdf]

Description of Additional Supplementary File

Supplementary Movie 1 | Reconstructing a mobbing flock from the original dataset. This video reconstructs the whole process of "mobbing-01" from the original mobbing dataset. For the individual present matrix (see Supplementary Figure 1b) of "mobbing-01", there are 167 rows (equals the number of birds present in this video) and 7439 columns (equals all the time stamps). As the frame rate of the original video recording is 60 frame/s, this flock lasts $7439/60 \approx 123.9833$ seconds. The gradient colors of trajectories from blue to red represent the flocking time from beginning to end. According to our workflow of data processing shown in Supplementary Figure 1, the "mobbing-01" flock is trimmed into 18 short periods of flocks, which are shown in Supplementary Figure 5.

Supplementary Movie 2 | Reconstructing a circling flock and highlighting the sub-communities in the flock. Due to the long duration of original circling dataset (each video recording lasted about 500 seconds, see x-axis in Supplementary Figure 4), we do not show the video of the whole process of "Circling A". Here we only reconstructed a circling flock, which is trimmed from the original dataset "Circling A" by our workflow of data processing. This flock is highlighted by a black box in Supplementary Figure 7. For the individual present matrix of this flock, there are 293 rows (equals the number of birds present in the video recording) and 189 columns (equals all the time stamps). As the frame rate of the original video recording is 30 frame/s, the flock lasts $189/30 = 6.30$ seconds. This video replays 2 times of the circling flock. At the first time of replay, the gradient colors of trajectories from blue to red represent the flocking time from beginning to end. At the second time of replay, the different colors correspond to the sub-communities embedded in the flock which are classified by the modularity of LF relation matrix. The inset figure to show the sub-communities is Supplementary Figure 10.

Supplementary Movie 3 | Reconstructing a transit flock from the original dataset. This video reconstructs the whole process of "transit-01" from the original transit dataset. For the individual present matrix of "transit-01", there are 502 rows (equals the number of birds present in the video recording) and 760 columns (equals all the time stamps). As the frame rate of the original video recording is 60 frame/s, the flock lasts $760/60 \approx 12.6667$ seconds. The gradient colors of trajectories from blue to red represent the flocking time from beginning to end. According to our workflow of data processing shown in Supplementary Figure 1, this "transit-01" flock is trimmed into 19 short periods of flocks, which are shown in Supplementary Figure 6.

Supplementary Movie 4 | Correlation analysis between LF and MS according to the mobbing flock shown in Fig.1a. We set the perceiving time $\tau = 7.43$ seconds (same with Fig.3a) to calculate the temporal LF and MS. The magenta arrow in the LF network indicates the LF relation from leader to follower. The size of circles in the MS matrix maps the values of $M_{ij}(t, \tau)$. The gradient colors (from blue to white to red) of the background of scatter plot of M_j and L_j correspond to the values of Spearman correlations (from -1 to 0 to 1) between two vectors composed of M_j and L_j, respectively. The gradient colors (from white to grey then to black) of ID at the top of each bird represents the increasing leading tier (from 0 to 1).

Supplementary Movie 5 | The swarm evacuates the narrow exit with width of 600mm based on three interaction types. We showed the evacuation process and compared the spatiotemporal distribution of the swarm based on AMS-100%, ATHD and average interaction. The swarm size is 50 and the width of exit is 600mm. For the parameters used in the swarm model of collective evacuation experiments see Supplementary Table 1.

Supplementary Movie 6 | The swarm evacuates the narrow exit with width of 700mm based on three interaction types. We showed the evacuation process and compared the spatiotemporal distribution of the swarm based on AMS-100%, ATHD and average interaction. The swarm size is 50 and the width of exit is 700mm. For the parameters used in the swarm model of collective evacuation experiments see Supplementary Table 1.

Supplementary Movie 7 | The swarm evacuates the narrow exit with width of 800mm based on three interaction types. We showed the evacuation process and compared the spatiotemporal distribution of the swarm based on AMS-100%, ATHD and average interaction. The swarm size is 50 and the width of exit is 800mm. For the parameters used in the swarm model of collective evacuation experiments see Supplementary Table 1.

Supplementary Movie 8 | The swarm evacuates the narrow exit with width of 600mm based on the semi-physical simulations. In the semi-physical simulator of Pybullet, we showed the evacuation process and compared the spatiotemporal distribution of the swarm based on AMS-100%, ATHD and average interaction. The swarm size is 50 and the width of exit is 600mm. The parameters used in the semi-physical simulations of collective evacuation is the same with real experiments.

Supplementary Movie 9 | A collective following experiment using AMS-100%. This video records the experiment process of Fig.5a. The swarm size is 50. For the parameters used in the swarm model of collective following experiments see Supplementary Table 1.

Supplementary Movie 10 | A collective following experiment using ATHD. The swarm size is 50. For the parameters used in the swarm model of collective following experiments see Supplementary Table 1.

Supplementary Movie 11 | A collective following experiment using the average interaction. This video records the experiment process of Fig.5d. The swarm size is 50. For the parameters used in the swarm model of collective following experiments see Supplementary Table 1.
