# Supplementary material for: Perception of motion salience shapes the emergence of collective motions
Source: Nat Commun. 2024 Jun 5;15:4779. doi: 10.1038/s41467-024-49151-x (PMC11153630; doi:10.1038/s41467-024-49151-x)
Supplement: Supplementary file 15 — Reporting Summary [file 41467_2024_49151_MOESM15_ESM.pdf]

nature portfolio

Corresponding author(s): Yandong Xiao

Last updated by author(s): May 6, 2024

Reporting Summary

Nature Portfolio wishes to improve the reproducibility of the work that we publish. This form provides structure for consistency and transparency in reporting. For further information on Nature Portfolio policies, see our Editorial Policies and the Editorial Policy Checklist.

Statistics

For all statistical analyses, confirm that the following items are present in the figure legend, table legend, main text, or Methods section.

n/a	Confirmed	
☐	☒	The exact sample size (*n*) for each experimental group/condition, given as a discrete number and unit of measurement
☐	☒	A statement on whether measurements were taken from distinct samples or whether the same sample was measured repeatedly
☒	☐	The statistical test(s) used AND whether they are one- or two-sided<br>*Only common tests should be described solely by name; describe more complex techniques in the Methods section.*
☒	☐	A description of all covariates tested
☒	☐	A description of any assumptions or corrections, such as tests of normality and adjustment for multiple comparisons
☐	☒	A full description of the statistical parameters including central tendency (e.g. means) or other basic estimates (e.g. regression coefficient) AND variation (e.g. standard deviation) or associated estimates of uncertainty (e.g. confidence intervals)
☐	☒	For null hypothesis testing, the test statistic (e.g. *F*, *t*, *r*) with confidence intervals, effect sizes, degrees of freedom and *P* value noted<br>*Give P values as exact values whenever suitable.*
☒	☐	For Bayesian analysis, information on the choice of priors and Markov chain Monte Carlo settings
☒	☐	For hierarchical and complex designs, identification of the appropriate level for tests and full reporting of outcomes
☒	☐	Estimates of effect sizes (e.g. Cohen's *d*, Pearson's *r*), indicating how they were calculated

Our web collection on statistics for biologists contains articles on many of the points above.

Software and code

Policy information about availability of computer code

Data collection	The mobbing and transit datasets have been deposited in https://figshare.com/s/472d354cc9e823a8f48f. The circling dataset is available at http://doi.org/10.5061/dryad.p68f8.
Data analysis	The computer codes used in this work are available at https://github.com/xiaoyandong08/Perception_of_Motion_Salience. The code are running in Matlab R2023b.

For manuscripts utilizing custom algorithms or software that are central to the research but not yet described in published literature, software must be made available to editors and reviewers. We strongly encourage code deposition in a community repository (e.g. GitHub). See the Nature Portfolio guidelines for submitting code & software for further information.

Data

Policy information about availability of data

All manuscripts must include a data availability statement. This statement should provide the following information, where applicable:

- Accession codes, unique identifiers, or web links for publicly available datasets
- A description of any restrictions on data availability
- For clinical datasets or third party data, please ensure that the statement adheres to our policy

Data availability

The mobbing and transit datasets from reference15 have been deposited in https://figshare.com/s/472d354cc9e823a8f48f. The circling dataset from reference16 is available at http://doi.org/10.5061/dryad.p68f8.

Research involving human participants, their data, or biological material

Policy information about studies with human participants or human data. See also policy information about sex, gender (identity/presentation), and sexual orientation and race, ethnicity and racism.

Reporting on sex and gender	No Human involves our study.
Reporting on race, ethnicity, or other socially relevant groupings	No Human involves our study.
Population characteristics	No Human involves our study.
Recruitment	No Human involves our study.
Ethics oversight	No Human involves our study.

Note that full information on the approval of the study protocol must also be provided in the manuscript.

Field-specific reporting

Please select the one below that is the best fit for your research. If you are not sure, read the appropriate sections before making your selection.

☐ Life sciences ☒ Behavioural & social sciences ☐ Ecological, evolutionary & environmental sciences

For a reference copy of the document with all sections, see nature.com/documents/nr-reporting-summary-flat.pdf

Behavioural & social sciences study design

All studies must disclose on these points even when the disclosure is negative.

Study description	We leveraged the high-resolution movement data of bird flocks with different motion patterns, i.e., mobbing, circling and transit to study the decentralized self-organization in animal groups which can help us design swarm robotics.
Research sample	The mobbing and transit datasets were obtained from the video recording of 3D movements of all individuals within flocks of wild jackdaws (Corvus monedul) in Cornwall, UK.<br><br>The circling dataset contained 3D tracks reconstructed from video recordings of a flock of ?1800 chimney swifts (Chaetura pelagica) in the field when the swifts entered an overnight roost in Raleigh, USA.
Sampling strategy	The original mobbing, circling, or transit bird-flocking datasets consisted of 12, 6 or 3 video recordings of 3D flocking trajectories. The resolution of video recordings of mobbing and transit datasets is 60 frame/s, and the circling dataset is 30 frame/s. See 'Supplementary Note 1: Workflow of data processing for the original flocking datasets' for details.
Data collection	The mobbing and transit datasets have been deposited in https://figshare.com/s/472d354cc9e823a8f48f. The circling dataset is available at http://doi.org/10.5061/dryad.p68f8.
Timing	We didn't involve Timing because we just use the public data from previous publications.
Data exclusions	No. See 'Supplementary Note 1: Workflow of data processing for the original flocking datasets' for details.
Non-participation	We didn't involve Non-participation because we just use the public data from previous publications.
Randomization	We didn't involve randomization because we just use the public data from previous publications.

Reporting for specific materials, systems and methods

We require information from authors about some types of materials, experimental systems and methods used in many studies. Here, indicate whether each material, system or method listed is relevant to your study. If you are not sure if a list item applies to your research, read the appropriate section before selecting a response.

Materials & experimental systems

n/a	Involved in the study
☒	☐ Antibodies
☒	☐ Eukaryotic cell lines
☒	☐ Palaeontology and archaeology
☒	☐ Animals and other organisms
☒	☐ Clinical data
☒	☐ Dual use research of concern
☒	☐ Plants

Methods

n/a	Involved in the study
☒	☐ ChIP-seq
☒	☐ Flow cytometry
☒	☐ MRI-based neuroimaging

Plants

Seed stocks	n/a
Novel plant genotypes	n/a
Authentication	n/a
